# Reductive Cyclization of *N*-Alkyl- and *N*-Aryl-*N*-(2-Nitrophenyl) Amides to 1,2-Disubstituted Benzimidazoles

**DOI:** 10.3390/molecules31122150

**Published:** 2026-06-18

**Authors:** Nash E. Nevels, Matthew E. Germond, Richard A. Bunce

**Affiliations:** Department of Chemistry, Oklahoma State University, Stillwater, OK 74078-3071, USA; nnevels@okstate.edu (N.E.N.); matthew.germond@okstate.edu (M.E.G.)

**Keywords:** 1*H*-benzo[*d*]imidazole, reductive cyclization, reduction-addition-aromatization, nitrogen heterocycle synthesis, drug precursors

## Abstract

A new three-step strategy for the preparation of 1,2-disubstituted 1*H*-benzo[*d*]imidazoles has been developed. The approach involved (1) S_N_Ar addition-elimination of alkyl- or arylamines to 1-fluoro-2-nitrobenzene to give *N*-alkyl- or *N*-aryl-2-nitroanilines, (2) acylation of these adducts with acid chlorides to afford *N*-alkyl- or *N*-aryl-*N*-(2-nitrophenyl) amides, and (3) reduction of the aromatic nitro with iron in acetic acid at 95–100 °C, followed by closure of the resulting amine on the amide carbonyl to produce 1,2-disubstituted benzimidazoles in high yields. The sequence gave the benzimidazole products in NMR-pure form with only one purification after Step 2. Thirty-seven derivatives were prepared, providing a broad selection of 1,2-disubstituted benzimidazoles. Interestingly, the reaction of the aniline nitrogen derived from the reduction of the nitro group afforded the final product by addition to the acyl carbonyl, followed by dehydrative aromatization with no competing rearrangement, acyl transfer, or side products.

## 1. Introduction

New methods to produce heterocyclic structures efficiently with high yields and minimal purification steps are a continuing goal in our laboratory. Most recently, we have reported expeditious routes to benzo[*d*]oxazoles [1] and 1*H*-indoles [2]. In the current study, we have applied a straightforward three-step reaction scheme to produce 1,2-disubstituted 1*H*-benzo[*d*]imidazoles, a scaffold that is highly coveted in drug chemistry. Our method features a reduction-cyclization-aromatization of *N*-alkyl- (or aryl)-*N*-(2-nitrophenyl) amides and delivers structures with predictable N1 and C2 substitution in good to excellent yields with only one purification.

**Figure 1:**
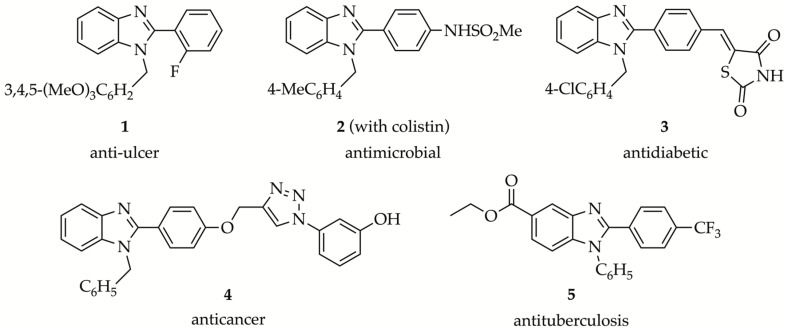
Representative 1,2-disubstituted 1*H*-benzo[*d*]imidazoles with potential drug activity.

Benzimidazoles have demonstrated diverse biological activities [3,4,5,6], and several with high potential as drugs are shown in Figure 1. Trimethoxybenzylbenzimidazole **1** has been shown to inhibit the growth of *H. pylori* and prevent its adhesion to and invasion of gastric epithelial cells [7], which may reduce the occurrence of peptic ulcers and gastric malignancies. Benzimidazole **2** has exhibited activity against Gram-negative bacteria when used synergistically with colistin. Minimum inhibitory concentration (MIC) ranges of 8–16 μg/mL against *E. coli*, *K. pneumoniae*, *A. baumannii*, and *P. aeruginosa* with no cytotoxicity have been observed in two mammalian cell lines [8]. Compound **3**, containing a 2,4-thiazolidinedione substituent at the para position of the C2 phenyl ring on the benzimidazole, expressed significant α-amylase and α-glucosidase inhibition with an IC_50_ of 0.54 ± 0.01 μM, and thus, may show promise as an antidiabetic agent [9]. Benzimidazole-triazole hybrid **4** demonstrated excellent growth inhibition against A-549 (IC_50_ = 0.63 ± 0.21 μM) and NCI-H460 (IC_50_ = 0.99 ± 0.01 μM) lung cancer cells, as well as MCF-7 (IC_50_ = 1.3 ± 0.18 μM) and MDA-MB-231 (IC_50_ = 0.94 ± 0.02 μM) breast cancer cells [10]. Finally, compound **5** has shown promising activity (IC_50_ = 8.96 μM and IC_90_ = 12.96 μM) against MTB-H_37_Rv, a drug-resistant strain of tuberculosis [11]. Each of these structures could easily be prepared using our approach to build structures with appropriate substitution for further elaboration.

There have been numerous syntheses of 1,2-disubstituted benzimidazoles, both with and without metals. The closest approach to the current work was offered by Shen and co-workers, who utilized an intermolecular iron-based reductive cyclization of 2-nitroanilines with orthoesters [12]. A second procedure, reported by Brain and Steer [13], utilized an intramolecular palladium-catalyzed arylation of (*Z*)-*N*′-2-haloarylamidines in base under microwave conditions. Several related amidine arylations were advanced to generate benzimidazoles using KOH in DMSO at 120 °C [14], CuO nanoparticles with base at 80–110 °C [15], I_2_/K_2_CO_3_ in CH_2_Cl_2_ at 25 °C [16], and PhI(OAc)_2_ in CH_3_CN at 120 °C [17]. Work by the Punniyamurthy group further detailed a Cu(OTf)_2_-mediated conversion of *N*-benzyl bisarylhydrazones to 2-aryl-1-benzylbenzimidazoles via a C–H functionalization C–N bond formation sequence in PhCH_3_ at 110 °C [18]. Additional strategies to generate the title heterocycles involved reaction of primary amines with phenylenediamine using an *ortho*-quinone catalyst [19] or with 1-alkylamino-2-nitroaromatics with FeCl_3_·6H_2_O [20]. Other redox-based methods condensed primary amines/alcohols with anilines in the presence of catalytic Cu(OAc)_2_, NaN_3_ and TBHP in DMSO at 80 °C [21], and with 1,2-diaminoaromatics using Et_3_SiH/KO-*t*-Bu in 1:1 dioxane/hexafluoroisopropyl alcohol at 140 °C [22], Na_2_S_2_O_4_ in DMSO/H_2_O at 90 °C [23] or a manganese(I) catalyst with KO-*t*-Bu neat at 140 °C [24]. Condensative approaches from 1,2-phenylenediamines and aldehydes are plentiful [25,26], with two recent examples being promoted by oxone [27] and (CH_3_)_3_SiCl [28]. Finally, a novel synthesis reported by Kundu et al. employed 9-mesityl-10-methylacridinium perchlorate as a photoactivation catalyst with HCl as a proton source to produce benzimidazoles from 1,2-diaminoaromatics and primary aliphatic alcohols [29].

While the above disclosures outline very elegant contributions, some require special reagents (e.g., transition metal complexes, *ortho*-quinones, 9-mesityl-10-methylacridinium perchlorate) that are not always available in organic chemistry laboratories. Furthermore, some syntheses require elaborate protocols with multiple additives. Therefore, a need exists for routes to access these ring systems efficiently with minimal expense. The current method provides a facile entry to *N*-alkyl-substituted benzimidazoles in only three steps from 2-fluoro-1-nitrobenzene, a primary amine, and an acid chloride, with iron powder/acetic acid to promote the reduction-cyclization-aromatization. The synthesis requires no special catalysts and thick-walled reaction vessels for reactions at temperatures 50–60 °C above the boiling point of the solvent are available and inexpensive. Thus, we present herein a straightforward strategy to generate a wide range of benzimidazoles for physical, synthetic, and biological studies.

## 2. Results and Discussion

**Scheme 1:**
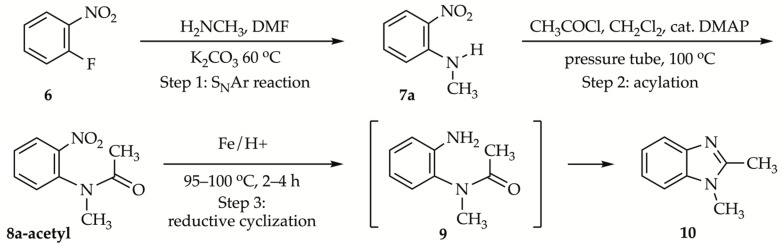
Synthetic plan to prepare 1,2-disubstituted benzimidazoles outlined for the conversion of **6** to **10**.

A three-step synthetic plan to prepare 1,2-disubstituted benzimidazoles was envisioned involving (1) S_N_Ar addition of a primary amine or aniline to 2-fluoro-1-nitrobenzene (**6**) to give an *N*-alkyl- or *N*-aryl-2-nitroaniline; (2) acylation of the aniline nitrogen with an acid chloride to afford an *N*-alkyl- or *N*-aryl-*N*-(2-nitrophenyl) amide; and (3) ring formation initiated by iron-promoted reduction of the aromatic nitro, followed by closure of the resulting amine on the acyl carbonyl to deliver the 1,2-disubstituted benzimidazole. Scheme 1 illustrates this sequence for the conversion of **6** to 1,2-dimethylbenzimidazole (**10**). As a general route, it was thought that Step 1 would present few problems. The acylation in Step 2, however, might prove difficult due to steric and electronic factors that could hinder access to the amine nitrogen and decrease the nucleophilicity of this group. This product would also consist of amide rotamers that would make the compound difficult to characterize by NMR at 25 °C. Finally, although the reduction-cyclization sequence seemed promising, the reaction course could terminate after simple reduction to **9** or undergo competitive acyl transfer (intramolecular transamidation) in addition to the anticipated aromatization. Nevertheless, since the reaction sequence was short, we decided to pursue the possibility of preparing and optimizing a synthesis of benzimidazoles using this route.

In Step 1, S_N_Ar addition of various primary alkylamines or anilines (1.2 equiv) to 2-fluoro-1-nitrobenzene (**6**, 1 equiv) in the presence of K_2_CO_3_ (1 equiv) at 60 °C for 12 h gave *N*-alkyl- (or aryl)-2-nitroanilines **7a**–**j**. Except for **7a** (R^1^ = CH_3_), prepared from methanolic methylamine, and **7i**–**k**, derived from aniline, 4-methoxyaniline, and 4-(trifluoromethyl)aniline, respectively, this reaction proceeded in 70–80% yield and did not require purification. The formation of **7a** from 40% methanolic methylamine (enough to contain 2 equiv of the amine) yielded 42% of **7a** along with *ca* 25% of 2-methoxy-1-nitrobenzene arising from deprotonation of the solvent and S_N_Ar by methoxide. The lower yields of **7i** (60%) from aniline and **7j** (55%) from 4-methoxyaniline likely derive from steric factors and reduced reactivity of the aniline nitrogen; highly electron-deficient 4-(trifluoromethoxy)aniline failed to give product **7k**. The R group of the amine (or aniline) in this step introduced the carbon group that became R^1^ in the final product. The results of the S_N_Ar replacement of fluoride in **6** by a series of amines to provide substrates **7a**–**j** are summarized in Table 1.

Treatment of **7a**–**7j** with acid chlorides (Step 2) was carried out in a 15-mL pressure tube using the amine (1.0 equiv), the acid chloride (1.3 equiv), and catalytic 4-(dimethylamino)pyridine (DMAP, 2–3 mg) in CH_2_Cl_2_ at 100 °C for 24 h to give the *N*-acylated products **8**. The use of a higher boiling solvent would preclude the use of a pressure vessel, but we opted to continue using CH_2_Cl_2_. Acylations of **7a**–**7h** proceeded normally, but the synthesis of 1-arylamino-2-nitrobenzenes **7i** and **7j** required 5–10 equiv of the acid chloride and heating at 100 °C for up to 1 week to be successful. Amides **8** isolated from this reaction could not be cleanly characterized as they consisted of a mixture of C–N rotamers that were contaminated with small quantities of the carboxylic acid derived from the acid chloride. ^1^H NMR spectra of these amides did not coalesce, even at 100 °C. Thus, we chromatographed these materials on silica gel (5–40% EtOAc in hexane) to remove traces of unreacted starting materials as well as the acid contaminant and subjected them directly to the ring closure conditions. Step 2 generally gave the lowest yields (ca. 60–80%) due to steric hindrance and reduced nucleophilicity of the aniline nitrogen conjugated with the nitro at C2 on the appended ring. The alkyl or aryl group from the acid chloride became R^2^ in the product benzimidazole. [Note: R^2^ in the product possessed one carbon less in its chain after ring closure occurred since the carbonyl carbon of the acid chloride became C2 of the benzimidazole].

The final cyclization (Step 3) was attempted on *N*-methyl-*N*-(2-nitrophenyl)acetamide (**8a-acetyl**, R^1^ = R^2^ = CH_3_) under two sets of conditions: one using powdered iron (3 equiv) and NH_4_Cl (1 equiv) in aqueous ethanol [30] and the other using powdered iron (3 equiv) in glacial acetic acid, each refluxed for 1 h. The reduction using NH_4_Cl in aqueous ethanol gave reduction of the nitro function but little cyclization (generating mostly **9**). The same reaction in glacial acetic acid, however, afforded benzimidazole **10** in 46% yield, along with recovered starting material and uncyclized reduction product. From here, we optimized the amount of iron to 6–8 equivalents with heating in acetic acid at 95–100 °C for 2 h to afford **10** in >98% yield (88% overall for Steps 2 and 3). The cyclizations typically proceeded under these conditions within 2–4 h as assessed by thin-layer chromatography (15–20% EtOAc in hexane). Excess iron did not decrease the yield. This reaction was worked up with 1 M aqueous NaOH and saturated aqueous NaCl solution to give the final products in NMR-pure form. The results of the acylation-ring closure sequence are summarized in Table 2.

The structures of the *N*-alkyl and *N*-aryl-2-nitroanilines (**7**) were readily compared via melting point to four known derivatives and NMR data previously reported for seven of these compounds. The *N*-alkyl- and *N*-aryl-*N*-(2-nitrophenyl) amides (**8**) from Step 2 proved impossible to characterize by ^1^H and ^13^C NMR. Due to restricted rotation about the amide C–N bond, their spectra showed broad or doubled absorptions for most of the signals, and a few still retained traces of the carboxylic acid derived from the acid chloride acylating agents, which co-eluted from the column chromatography. At elevated temperatures up to 100 °C, the ^1^H NMR remained complex. Following reduction and ring closure, however, the spectra were greatly simplified (see the Supplemental Information). Twelve of the benzimidazoles prepared matched the literature spectra of previously prepared derivatives. Due to the lack of substitution on the fused benzene of the benzimidazole, the signals for these four protons were all multiplets. Para-disubstituted aromatic substituents on N1 or at C2 displayed a distinctive AA’BB’ pattern in the aromatic region of the spectrum. For the N1 benzyl-substituted benzimidazoles, the methylene group appeared as a singlet at *ca* δ 5.4, while the phenethylamine-derived structures displayed two triplets at *ca* δ 4.4 and δ 3.1 for the coupled methylene protons; these same methylenes presented as multiplets at *ca* δ 3.2 and δ 3.1 when the phenethyl group was positioned at C2 of the heterocycle. Products derived from aliphatic precursors exhibited predictable multiplicities and chemical shifts for hydrogens on substituents at N1 and C2. Despite the use of iron in the final reductive cyclization, the benzimidazole products furnished clean spectra with no signal broadening due to residual iron and acceptable elemental analyses for previously unreported structures. Although analytical samples were accessible by column chromatography, this process was tedious, and elution required 40–60% EtOAc in hexane. Thus, benzimidazole NMRs were recorded without a final purification. ^13^C {^1^H} and ^19^F {^1^H} NMR were somewhat less useful for structure elucidation but did provide insight into the numbers and types of carbons and fluorines (when present), respectively.

**Scheme 2:**
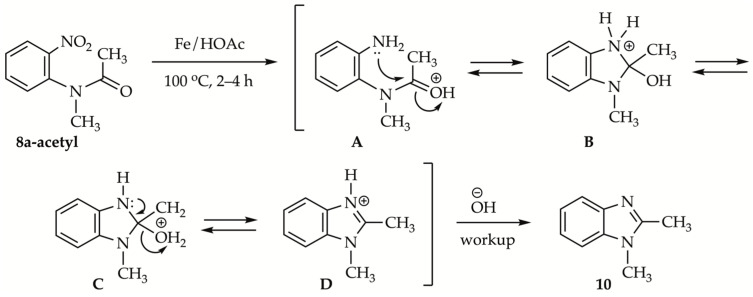
Mechanism for the reductive ring closure of **8a-acetyl** to **10**.

The mechanism for the final reductive cyclization of compound **8a-acetyl** to give 1,2-dimethyl-1*H*-benzo[*d*]imidazole (**10**) is shown in Scheme 2. The cyclization involved reduction of the nitro to an amino group [31] to give intermediate **A**, cyclization to the acetyl carbonyl to yield **B**, proton exchange to afford **C**, and dehydrative aromatization to give **D**. Basic workup then furnished product **10**. Interestingly, only ring closure to produce the benzimidazole was observed. None of the products from acyl-transfer [32] to the amine function of the aniline were detected.

## 3. Materials and Methods

### 3.1. General Methods

Unless otherwise indicated, all reactions were performed under N_2_ in dry glassware. All commercial reagents and solvents were used as received. Reactions were followed by TLC on Analtech (now Miles Scientific) No 21521 silica gel GF plates (Newark, DE, USA). Preparative separations were performed on Davisil^®^ grade 62, 70–200 mesh silica gel (Avantor/VWR, Allentown, PA, USA) containing 0.5% of UV-05 indicator (Sorbent Technologies, Norcross, GA, USA) slurry packed into quartz columns. Band elution for all chromatographic separations was detected using a hand-held UV lamp (Fisher Scientific, Pittsburgh, PA, USA). Melting points (uncorrected) were obtained using a MEL–TEMP apparatus (Laboratory Devices, Cambridge, MA, USA). FT-IR spectra were run using a Nicolet Summit X FTIR spectrometer with an Everest ATR accessory (Thermo Fisher Scientific, Waltham, MA, USA). ^1^H and ^13^C {^1^H} NMR spectra were measured at 298 K (25 °C) using a Bruker Avance 400 system (Billerica, MA, USA) at 400 MHz and 101 MHz, respectively, in CDCl_3_ containing 0.05% *v*/*v* tetramethylsilane (TMS) as the internal standard (Cambridge Isotope, Andover, MA, USA). Chemical shifts are given in δ (ppm) units relative to TMS and coupling constants (*J*) are given in Hz. ^19^F {^1^H} NMR spectra (376 MHz, CDCl_3_), obtained on the same instrument, are referenced to internal C_6_H_5_F at δ −113.15. Low-resolution mass spectra were acquired using a Hewlett-Packard Model 1800A GCD GC–MS instrument (Palo Alto, CA, USA). Elemental analyses (±0.3%) on all new compounds were determined by Atlantic Microlabs, Inc. (Norcross, GA, USA).

### 3.2. Synthesis of N-alkyl-2-nitroanilines

A 100 mL round-bottomed flask equipped with a magnetic stir bar was charged with anhydrous DMF (10 mL), 1-fluoro-2-nitrobenzene (1.41 g, 10 mmol), and the amine (10.2 mmol). To this mixture was added anhydrous K_2_CO_3_ (1.38 g, 10 mmol), and the reaction was heated to 60 °C with stirring for 24 h. The reaction was cooled and added to 1 M aqueous NH_4_Cl (100 mL) and washed with EtOAc (2 × 25 mL). The combined ether extracts were washed with H_2_O (2 × 50 mL) and saturated aqueous NaCl (1 × 50 mL) and then dried (MgSO_4_). Filtration and concentration gave the crude product as an orange oil. This product was generally used without further purification. Analytical samples were obtained by silica gel column chromatography eluted with 5% EtOAc in hexanes, which gave the *N*-alkyl-2-nitroanilines as orange solids or oils.

#### 3.2.1. N-Methyl-2-nitroaniline (**7a**)

Yield: 0.64 g (4.2 mmol, 42%) as an orange solid, m.p. 33–35 °C [lit. [33] m.p. 36 °C]; IR: 3400, 1618, 1583, 1515, 1448, 1362, 1272, 1238, 1182 cm^−1^; ^1^H NMR (400 MHz, CDCl_3_): δ 8.17 (dd, *J* = 8.6, 1.6 Hz, 1H), 8.04 (br s, 1H), 7.46 (ddd, *J* = 8.6, 7.0, 1.6 Hz, 1H), 6.84 (dd, *J* = 8.6, 1.3 Hz, 1H), 6.65 (ddd, *J* = 8.4, 7.0, 1.3 Hz, 1H), 3.02 (s, 3H); ^13^C {^1^H} NMR (101 MHz, CDCl_3_): δ 146.4, 136.3, 131.9, 126.8, 115.2, 113.4, 29.7; MS (*m*/*z*): 152. These spectral data matched those previously reported [33]. Note: In this preparation, a 40% solution of methylamine in CH_3_OH was used in sufficient quantity to provide 2 equivalents of CH_3_NH_2_. This resulted in a lower yield of the aniline with the formation of *ca* 25% of 2-methoxy-1-nitrobenzene from S_N_Ar reaction of deprotonated CH_3_OH as a byproduct. This contaminant was removed by chromatographic purification.

#### 3.2.2. N-Isobutyl-2-nitroaniline (**7b**)

Yield: 1.40 g (7.2 mmol, 72%) as an orange oil; IR: 3391, 1610, 1508, 1392, 1360, 1268, 1228 cm^−1^; ^1^H NMR (400 MHz, CDCl_3_): δ 8.16 (overlapping dd, *J* = 8.6, 1.6 Hz, 1H and br s, 1H), 7.41 (apparent t, *J* = 8.6 Hz, 1H), 6.83 (dd, *J* = 8.6, 1.2 Hz, 1H), 6.61 (m, 1H), 3.12 (t, *J* = 6.7 Hz, 2H), 2.01 (nonet, *J* = 6.6 Hz, 1H), 1.05 (d, *J* = 6.7 Hz, 6H); ^13^C {^1^H} NMR (101 MHz, CDCl_3_): δ 145.8, 136.2, 131.7, 126.9, 115.0, 113.8, 50.7, 28.0, 20.4; MS (*m*/*z*): 194. The NMR spectral data matched those previously reported [34].

#### 3.2.3. N-Hexyl-2-nitroaniline (**7c**)

Yield: 1.64 g (7.4 mmol, 74%) as an orange oil; IR: 3392, 1611, 1577, 1516, 1419, 1351, 1278, 1230, 1163 cm^−1^; ^1^H NMR (400 MHz, CDCl_3_): δ 8.15 (dd, *J* = 8.7, 1.7 Hz, 1H), 8.05 (br s, 1H), 7.42 (ddd, *J* = 8.7, 6.9, 1.7 Hz, 1H), 6.84 (dd, *J* = 8.7, 1.3 Hz, 1H), 6.61 (ddd, *J* = 8.4, 6.9, 1.3 Hz, 1H), 3.29 (m, 2H), 1.72 (pentet, *J* = 7.1 Hz, 2H), 1.45 (m, 2H), 1.33 (m, 4H), 0.91 (distorted t, *J* = 7.1 Hz, 3H); ^13^C {^1^H} NMR (101 MHz, CDCl_3_): δ 144.6, 136.7, 131.7, 126.9, 115.0, 113.8, 43.1, 31.5, 28.9, 26.8, 22.6, 14.0; MS (*m*/*z*): 222. The NMR spectral data matched those previously reported [35].

#### 3.2.4. N-(3-Isopropoxypropyl)-2-nitroaniline (**7d**)

Yield: 1.95 g (8.2 mmol, 82%) as an orange oil; IR: 3400, 1512, 1339, 1179, 1075 cm^−1^; ^1^H NMR (400 MHz, CDCl_3_): δ 8.24 (br s, 1H), 8.14 (dd, *J* = 8.6, 1.6 Hz, 1H), 7.41 (ddd, *J* = 8.6, 6.9, 1.7 Hz, 1H), 6.87 (dd, *J* = 8.8, 1.2 Hz, 1H), 6.60 (ddd, *J* = 8.3, 6.9, 1.2 Hz, 1H), 3.57 (septet, *J* = 6.1 Hz, 1H), 3.56 (t, *J* = 5.8 Hz, 2H), 3.42 (br q, *J* = 5.2 Hz, 2H), 1.96 (pentet, *J* = 5.8 Hz, 2H), 1.18 (d, *J* = 6.2 Hz, 6H); ^13^C {^1^H} NMR (101 MHz, CDCl_3_): δ 145.6, 136.1, 131.7, 126.8, 114.9, 113.8, 71.9, 65.7, 40.9, 29.4, 22.0; MS (*m*/*z*): 238; Anal. Calcd for C_12_H_18_N_2_O_3_: C, 60.49; H, 7.61; N, 11.76. Found: C, 60.34; H, 7.54; N, 11.73.

#### 3.2.5. N-Benzyl-2-nitroaniline (**7e**)

Yield: 1.85 g (8.1 mmol, 81%) as an orange solid, m.p. 75–77 °C (lit. [36] m.p. 76–78 °C); IR: 3398, 1615, 1591, 1529, 1467, 1414, 1352, 1280, 1246, 1168 cm^−1^; ^1^H NMR (400 MHz, CDCl_3_): δ 8.43 (br s, 1H), 8.20 (dd, *J* = 8.7, 1.7 Hz, 1H), 7.41–7.28 (complex 6H), 6.82 (dd, *J* = 8.7, 1.2 Hz, 1H), 6.67 (ddd, *J* = 8.4, 7.0, 1.2 Hz, 1H), 4.56 (d, *J* = 5.6 Hz, 2H); ^13^C {^1^H} NMR (101 MHz, CDCl_3_): δ 145.3, 137.4, 136.2, 132.3, 128.9, 127.7, 127.0, 126.9, 115.7, 114.2, 47.1; MS (*m*/*z*): 228. The NMR spectral data matched those previously reported [36].

#### 3.2.6. 2-Nitro-N-phenethylaniline (**7f**)

Yield: 1.94 g (8.0 mmol, 80%) as an orange solid, m.p. 70–72 °C (lit. [37] m.p. 71.5–72.5 °C); IR: 3384, 1608, 1578, 1510, 1414, 1362, 1270, 1222, 1153 cm^−1^; ^1^H NMR (400 MHz, CDCl_3_): δ 8.16 (dd, *J* = 8.6, 1.6 Hz, 1H), 8.09 (br s, 1H) 7.42 (ddd, *J* = 8.6, 6.9, 1.6 Hz, 1H), 7.37–7.31 (complex, 2H), 7.29–7.23 (complex, 3H), 6.85 (dd, *J* = 8.7, 1.2 Hz, 1H), 6.64 (ddd, *J* = 8.4, 7.0, 1.2 Hz, 1H), 3.57 (m, 2H), 3.02 (t, *J* = 7.1 Hz, 2H); ^13^C {^1^H} NMR (101 MHz, CDCl_3_): δ 145.3, 138.3, 136.2, 131.9, 128.8, 128.7, 126.9, 126.8, 115.3, 113.7, 44.5, 35.3; MS (*m*/*z*): 242. The ^1^H NMR spectral data matched those previously reported [38].

#### 3.2.7. N-(4-Methoxybenzyl)-2-nitroaniline (**7g**)

Yield: 2.01 g (7.8 mmol, 78%) as an orange solid, m.p. 92–93 °C (lit. [39] m.p. 94–95 °C); IR: 3393, 2838, 1607, 1578, 1500, 1410, 1358, 1252, 1230, 1155, 1018 cm^−1^; ^1^H NMR (400 MHz, CDCl_3_): δ 8.35 (br s, 1H), 8.19 (dd, *J* = 8.6, 1.6 Hz, 1H), 7.39 (ddd, *J* = 8.7, 7.0, 1.6 Hz, 1H), 7.27 (d, *J* = 8.7 Hz, 2H), 6.89 (d, *J* = 8.7 Hz, 2H), 6.83 (dd, *J* = 8.7, 1.2 Hz, 1H), 6.66 (ddd, *J* = 8.4, 7.0, 1.2 Hz, 1H), 4.48 (d, *J* = 5.4 Hz, 2H), 3.81 (s, 3H); ^13^C {^1^H} NMR (101 MHz, CDCl_3_): δ 159.2, 145.2, 136.2, 132.2, 129.3, 128.4, 126.9, 115.6, 114.3, 114.2, 55.3, 46.6; MS (*m*/*z*): 258. The ^1^H NMR spectral data matched those previously reported [39].

#### 3.2.8. 2-Nitro-N-(4-(trifluoromethyl)benzyl)aniline (**7h**)

Yield: 2.10 g (7.1 mmol, 71%) as an orange solid, m.p. 90–92 °C; IR: 3396, 1604, 1581, 1515, 1443, 1420, 1343, 1318, 1248, 1125, 1076 cm^−1^; ^1^H NMR (400 MHz, CDCl_3_): δ 8.48 (br s, 1H), 8.22 (dd, *J* = 8.6, 1.4 Hz, 1H), 7.63 (d, *J* = 8.1 Hz, 2H), 7.47 (d, *J* = 8.1 Hz, 2H), 7.39 (ddd, *J* = 8.6, 6.9, 1.6 Hz, 1H), 6.71 (overlapping dd, *J* = 8.4, 1.7 Hz, 1H and ddd, *J* = 8.3, 7.0, 1.2 Hz, 1H), 4.64 (d, *J* = 5.9 Hz, 2H); ^13^C {^1^H} NMR (101 MHz, CDCl_3_): δ 144.9, 141.6, 136.3, 132.6, 130.1 (q, *J* = 32.6 Hz), 127.1, 127.0, 125.9 (q, *J* = 3.7 Hz), 124.0 (q, *J* = 272.2 Hz), 116.2, 114.0, 46.6; ^19^F {^1^H} NMR (376 MHz, CDCl_3_): δ −62.6; MS (*m*/*z*): 296; Anal. Calcd for C_14_H_11_F_3_N_2_O_2_: C, 56.76; H, 5.30; N, 12.27. Found: C, 56.62; H, 5.21; N, 12.35.

#### 3.2.9. 2-Nitro-N-phenylaniline (**7i**)

Yield (5.0 mmol scale): 0.64 g (3.0 mmol, 60%) as an orange oil; IR: 3355, 1608, 1598, 1578, 1499, 1350, 1265, 1237, 1132 cm^−1^; ^1^H NMR (400 MHz, CDCl_3_): δ 9.49 (s, 1H), 8.20 (dd, *J* = 8.6, 1.6 Hz, 2H), 7.41 (t, *J* = 8.4 Hz, 2H), 7.35 (ddd, *J* = 8.5, 6.8, 1.6 Hz, 1H), 7.28 (d, *J* = 8.5 Hz, 2H), 7.23 (t, *J* = 8.5 Hz, 2H); ^13^C {^1^H} NMR (101 MHz, CDCl_3_): δ 143.1, 138.7, 135.7, 133.2, 129.7, 126.7, 125.7, 124.4, 117.5, 116.0; MS (*m*/*z*): 214. The NMR spectral data matched those previously reported [40].

#### 3.2.10. N-(4-Methoxyphenyl)-2-nitroaniline (**7j**)

Yield (5.0 mmol scale): 0.67 g (2.8 mmol, 55%) as an orange solid, m.p. 86–88 °C; IR: 3324, 2838, 1618, 1568, 1502, 1349, 1242, 1220 cm^−1^; ^1^H NMR (400 MHz, CDCl_3_): δ 9.40 (s, 1H), 8.19 (dd, *J* = 8.7, 1.6 Hz, 1H), 7.32 (ddd, *J* = 8.7, 6.9, 1.6 Hz, 1H), 7.20 (d, *J* = 8.7 Hz, 2H), 7.00 (dd, *J* = 8.7, 1.3 Hz, 1H), 6.96 (d, *J* = 8.7 Hz, 2H), 6.71 (ddd, *J* = 8.4, 6.9, 1.3 Hz, 1H), 3.84 (s, 3H); ^13^C {^1^H} NMR (101 MHz, CDCl_3_): δ 158.0, 144.5, 135.7, 132.5, 131.2, 127.1, 126.6, 116.8, 115.8, 115.0, 55.5; MS (*m*/*z*): 244. The NMR spectral data matched those previously reported [40].

### 3.3. Synthesis of 1,2-disubstituted 1H-benzo[d]imidazoles

A 15 mL thick-walled pressure tube (ChemGlass, Vineland, NJ, USA), equipped with a magnetic stir bar, was charged with the *N*-alkyl (or aryl)-2-nitroaniline (250 mg) in dichloromethane (DCM, 6 mL), and 2–3 mg of DMAP was added. To this solution was added the acid chloride (1.3 equiv), the Teflon^®^ (PTFE) cap (with a perfluoro O-ring) was screwed on tightly, and the reaction was heated at 100 °C for 24 h. At this time, the reaction was cooled and poured into 1 M NaOH (25 mL). For reactions requiring 5–10 equiv of the acid chloride, the reaction was cooled and washed with 1 M NaOH (2 × 25 mL). The two phases were separated, and the aqueous layer was extracted a second time with DCM. The combined organic extracts were washed with H_2_O (1 × 25 mL) and saturated NaCl (2 × 25 mL), and dried (MgSO_4_). Filtration and concentration then gave the crude *N*-alkyl (or aryl)-*N*-(2-nitrophenyl) amide. As some of these products contained small amounts of starting material and the carboxylic acid derived from the acid chloride, a short silica gel column chromatography (25 cm × 2.5 cm) eluted with 5–40% EtOAc in hexane was used to purify these materials.

A 25 mL round-bottomed flask equipped with magnetic stirring was charged with a solution of the *N*-alkyl or *N*-aryl-*N*-(2-nitrophenyl) amide (150 mg) in glacial acetic acid (6 mL). The solution was heated to 90 °C, 100 mesh iron powder (6–8 equiv) was added with vigorous stirring, a condenser was inserted in the flask, and the reaction was heated at 95–100 °C for 2–4 h. When complete, TLC indicated the starting amide was completely consumed. The reaction was cooled, the stir bar and unreacted iron were removed with a polypropylene-coated magnetic stir bar retriever (Grainger, Oklahoma City, OK, USA), and the mixture was concentrated under vacuum. The crude material was transferred to a separatory funnel with EtOAc and washed with H_2_O (1 × 25 mL), saturated aqueous 1 M NaOH (1 × 25 mL), and saturated aqueous NaCl (1 × 25 mL). The solution was dried (MgSO_4_) and concentrated under vacuum to give the benzimidazole product as a tan or yellow solid or oil. All samples proved to be pure by NMR, but analytical samples could be obtained via silica gel column chromatography using 40–60% EtOAc in hexane. Yields reported are for the two-step sequence.

#### 3.3.1. 1,2-Dimethyl-1H-benzo[*d*]imidazole (**10**)

From **7a** and acetyl chloride; Yield: 85 mg (0.58 mmol, 88%) as a tan solid, m.p. 107–109 °C (lit. [13] m.p. 106–109 °C); IR: 1651, 1572, 1512, 1398, 1325, 1281, 1234 cm^−1^; ^1^H NMR (400 MHz, CDCl_3_): δ 7.69 (m, 1H), 7.32–7.22 (complex, 3H), 3.73 (s, 3H), 2.61 (s, 3H); ^13^C {^1^H} NMR (101 MHz, CDCl_3_): δ 151.7, 142.0, 135.6, 122.2, 122.1, 118.8, 108.9, 29.9, 13.7; MS (*m*/*z*): 146. The NMR spectral data matched those previously reported [13].

#### 3.3.2. 1-Methyl-2-phenyl-1H-benzo[*d*]imidazole (**11**)

From **7a** and benzoyl chloride; Yield: 117 mg (0.56 mmol, 85%) as a tan solid, m.p. 92–93 °C (lit [13] m.p. 92–95 °C); IR: 1652, 1576, 1490, 1398, 1332, 1259 cm^−1^; ^1^H NMR (400 MHz, CDCl_3_): δ 7.83 (m, 1H), 7.76 (m, 2H), 7.56–7.48 (complex, 3H), 7.39 (m, 1H), 7.35–7.28 (complex, 2H), 3.85 (s, 3H); ^13^C {^1^H} NMR (101 MHz, CDCl_3_): δ 153.8, 143.0, 136.6, 130.2, 129.8, 129.5, 128.7, 122.8, 122.8, 119.9, 109.6, 31.7; MS (*m*/*z*): 208. The NMR spectral data matched those previously reported [13].

#### 3.3.3. 1-Methyl-2-phenethyl-1H-benzo[*d*]imidazole (**12**)

From **7a** and 3-phenylpropionyl chloride; Yield: 132 mg (0.56 mmol, 85%) as a tan oil; IR: 1605, 1518, 1477, 1450, 1400, 1328, 1236 cm^−1^; ^1^H NMR (400 MHz, CDCl_3_): δ 7.74 (m, 1H), 7.28–7.11 (complex, 8H), 3.31 (s, 3H), 3.15 (m, 2H), 3.04 (m, 2H); ^13^C {^1^H} NMR (101 MHz, CDCl_3_): δ 154.4, 142.3, 140.8, 135.5, 128.6, 128.4, 126.5, 122.2, 122.0, 119.0, 109.1, 34.1, 29.5, 29.4; MS (*m*/*z*): 236. The NMR spectral data matched those previously reported [41].

#### 3.3.4. 2-(4-Methoxyphenyl)-1-methyl-1H-benzo[*d*]imidazole (**13**)

From **7a** and 4-methoxybenzoyl chloride; Yield: 124 g (0.52 mol, 79%) as a tan solid, m.p. 75–77 °C; IR: 2838, 1601, 1452, 1430, 1375, 1126, 1020, 840 cm^−1^; ^1^H NMR (400 MHz, CDCl_3_): δ 7.83 (m, 1H), 7.72 (d, *J* = 8.8 Hz, 2H), 7.38 (m, 1H), 7.33–7.27 (complex, 2H), 7.05 (d, *J* = 8.8 Hz, 2H), 3.89 (s, 3H), 3.85 (s, 3H); ^13^C {^1^H} NMR (101 MHz, CDCl_3_): δ 160.8, 153.7, 142.7, 136.5, 130.9, 122.6, 122.44, 122.35, 119.6, 114.2, 109.5, 55.4, 31.1; MS (*m*/*z*): 238; Anal. Calcd for C_15_H_14_N_2_O: C, 75.61; H, 5.92; N, 11.76. Found: C, 75.36; H, 5.87; N, 11.48.

#### 3.3.5. 2-(3-Chlorophenyl)-1-methyl-1H-benzo[*d*]imidazole (**14**)

From **7a** and 3-chlorobenzoyl chloride; Yield: 125 mg (0.51 mmol, 78%) as a tan oil; IR: 1610, 1583, 1478, 1445, 1390, 1338 cm^−1^; ^1^H NMR (400 MHz, CDCl_3_): δ 7.83 (m, 1H), 7.79 (t, *J* = 1.9 Hz, 1H), 7.65 (dt, *J* = 6.8, 1.8 Hz, 1H), 7.51–7.46 (complex, 2H), 7.40 (m, 1H), 7.37–7.29 (complex, 2H), 3.87 (s, 3H); ^13^C {^1^H} NMR (101 MHz, CDCl_3_): δ 152.2, 142.8, 136.6, 134.9, 132.0, 130.0, 129.9, 129.5, 127.5, 123.2, 122.7, 120.0, 109.7, 31.7; MS (*m*/*z*): 242. The NMR spectral data matched those previously reported [42].

#### 3.3.6. 1-Isobutyl-2-methyl-1H-benzo[*d*]imidazole (**15**)

From **7b** and acetyl chloride; Yield: 70 mg (0.37 mmol, 72%) as a tan solid, m.p. 60–62 °C; IR: 1608, 1509, 1468, 1398, 1321, 1276, 1130 cm^−1^; ^1^H NMR (400 MHz, CDCl_3_): δ 7.68 (m, 1H), 7.26 (m, 1H), 7.21 (m, 2H), 3.87 (m, 2H), 2.59 (s, 3H), 2.22 (nonet, *J* = 6.7 Hz, 1H), 0.95 (d, *J* = 6.7 Hz, 6H); ^13^C {^1^H} NMR (101 MHz, CDCl_3_): δ 151.7, 142.6, 135.5, 121.8, 121.7, 119.0, 109.5, 51.3, 29.1, 20.2, 14.2; MS (*m*/*z*): 188; Anal. Calcd for C_12_H_16_N_2_: C, 76.55; H, 8.57; N, 14.88. Found: C, 76.45; H, 8.49; N, 14.91.

#### 3.3.7. 1-Isobutyl-2-phenyl-1H-benzo[*d*]imidazole (**16**)

From **7b** and benzoyl chloride; Yield: 80 mg (0.41 mmol, 70%) as a tan oil; IR: 1605, 1522, 1475, 1448, 1395, 1326 cm^−1^; ^1^H NMR (400 MHz, CDCl_3_): δ 7.83 (m, 1H), 7.67 (m, 2H), 7.52–7.46 (complex, 3H), 7.40 (m, 1H), 7.32–7.26 (complex, 2H), 4.07 (br d, *J* = 7.6 Hz, 2H), 2.11 (m, 1H), 0.72 (br d, *J* = 6.7 Hz, 6H); ^13^C {^1^H} NMR (101 MHz, CDCl_3_): δ 154.2, 143.1, 135.8, 131.1, 129.6, 129.5, 128.7, 122.6, 122.3, 120.0, 110.5, 51.9, 28.8, 20.0; MS (*m*/*z*): 250; Anal. Calcd for C_17_H_18_N_2_: C, 81.56; H, 7.25; N, 11.19. Found: C, 81.51; H, 7.23; N: 11.25.

#### 3.3.8. 1-Isobutyl-2-(4-methoxyphenyl)-1H-benzo[*d*]imidazole (**17**)

From **7b** and 4-methoxybenzoyl chloride; Yield: 111 mg (0.40 mmol, 77%) as a tan oil; IR: 2839, 1611, 1494, 1467, 1395, 1260, 842 cm^−1^; ^1^H NMR (400 MHz, CDCl_3_): δ 7.80 (m, 1H), 7.63 (d, *J* = 8.7 Hz, 2H), 7.39 (m, 1H), 7.29–7.26 (complex, 2H), 7.02 (d, *J* = 8.7 Hz, 2H), 4.07 (d, *J* = 7.6 Hz, 2H), 3.87 (s, 3H), 2.14 (nonet, *J* = 6.9 Hz, 1H), 0.75 (d, *J* = 6.7 Hz, 6H); ^13^C {^1^H} NMR (101 MHz, CDCl_3_): δ 160.6, 154.1, 143.0, 135.8, 130.9, 123.3, 122.4, 122.2, 119.7, 114.1, 110.4, 55.4, 51.9, 28.8, 20.1; MS (*m*/*z*): 280; Anal. Calcd for C_18_H_20_N_2_O: C, 77.11; H, 7.19; N, 9.99. Found: C, 77.02; H, 7.23; N, 9.87.

#### 3.3.9. 1-Isobutyl-2-(4-(trifluoromethyl)phenyl)-1H-benzo[*d*]imidazole (**18**)

From **7b** and 4-(trifluoromethyl)benzoyl chloride; Yield: 102 mg (0.32 mmol, 62%) as a yellow solid, m.p. 93–94 °C; IR: 1616, 1532, 1462, 1418, 1327, 1174, 1128, 851 cm^−1^; ^1^H NMR (400 MHz, CDCl_3_): δ 7.84 (m, 1H), 7.83 (d, *J* = 8.2 Hz, 2H), 7.78 (d, *J* = 8.2 Hz, 2H), 7.43 (m, 1H), 7.36–7.29 (complex, 2H), 4.09 (d, *J* = 7.6 Hz, 2H), 2.12 (m, 1H), 0.74 (d, *J* = 6.7 Hz, 6H); ^13^C {^1^H} NMR (101 MHz, CDCl_3_): δ 152.4, 143.0, 135.9, 134.7 (q, *J* = 1.6 Hz), 131.5 (q, *J* = 32.8 Hz), 128.0, 125.7 (q, *J* = 3.8 Hz), 123.9 (q, *J* = 272.3 Hz), 123.2, 122.7, 120.2, 110.7, 52.1, 28.9, 20.0; ^19^F {^1^H} NMR (376 MHz, CDCl_3_): δ −62.8; MS (*m*/*z*): 318; Anal. Calcd for C_18_H_17_FN_2_: C, 67.91; H, 5.38; N, 8.80. Found: C, 67.99; H, 5.33; N: 8.73.

#### 3.3.10. 1-Hexyl-2-isopropyl-1H-benzo[*d*]imidazole (**19**)

From **7c** and isobutyryl chloride; Yield: 82 mg (0.33 mmol, 73%) as a light yellow oil; IR: 1609, 1514, 1479, 1418, 1314, 1288, 1091 cm^−1^; ^1^H NMR (400 MHz, CDCl_3_): δ 7.75 (m, 1H), 7.29 (m, 1H), 7.22 (m, 2H), 4.10 (t, *J* = 7.6 Hz, 2H), 3.18 (septet, *J* = 6.8 Hz, 1H), 1.80 (pentet, *J* = 7.3 Hz, 2H), 1.45 (d, *J* = 6.9 Hz, 6H), 1.41–1.25 (complex, 6H), 1.22 (distorted t, *J* = 6.8 Hz, 3H); ^13^C {^1^H} NMR (101 MHz, CDCl_3_): δ 159.7, 142.7, 134.9, 121.8, 121.6, 119.3, 109.3, 43.5, 31.4, 30.1, 26.7, 26.4, 22.5, 21.9, 14.0; MS (*m*/*z*): 244; Anal. Calcd for C_16_H_24_N_2_: C, 78.64; H, 9.90; N, 11.46. Found: C, 78.47; H, 9.85; N, 11.29.

#### 3.3.11. 1-Hexyl-2-phenyl-1H-benzo[*d*]imidazole (**20**)

From **7c** and benzoyl chloride; Yield: 89 mg (0.32 mmol, 71%) as a light yellow oil; IR: 1605, 1518, 1476, 1440, 1395, 1318, 1288, 1248 cm^−1^; ^1^H NMR (400 MHz, CDCl_3_): δ 7.83 (m, 1H), 7.71 (m, 2H), 7.55–7.47 (complex, 3H), 7.41 (m, 1H), 7.33–7.27 (complex, 2H), 4.22 (t, *J* = 7.7 Hz, 2H), 7.22 (pentet, *J* = 7.7 Hz, 2H), 1.30–1.15 (complex, 6H), 0.83 (distorted t, *J* = 6.9 Hz, 3H); ^13^C {^1^H} NMR (101 MHz, CDCl_3_): δ 153.7, 143.1, 135.6, 130.7, 129.7, 129.3, 128.7, 122.6, 122.3, 120.0, 110.1, 44.7, 31.1, 29.7, 26.3, 22.4, 13.9; MS (*m*/*z*): 278. The NMR spectral data matched those previously reported [43].

#### 3.3.12. 1-Hexyl-2-phenethyl-1H-benzo[*d*]imidazole (**21**)

From **7c** and 3-phenylpropionyl chloride; Yield: 98 mg (0.32 mmol, 72%) as a tan oil; IR: 1610, 1511, 1462, 1404, 1328, 1290, 1262 cm^−1^; ^1^H NMR (400 MHz, CDCl_3_): δ 7.77 (m, 2H), 7.30–7.15 (complex, 8H), 3.89 (t, *J* = 7.4 Hz, 2H), 3.25 (ABm, 2H), 3.11 (ABm, 2H), 1.63 (pentet, *J* = 7.1 Hz, 2H), 1.31–1.18 (complex, 6H), 0.85 (distorted t, *J* = 6.6 Hz, 3H); ^13^C {^1^H} NMR (101 MHz, CDCl_3_): δ 154.1, 142.7, 141.1, 135.0, 128.7, 128.5, 126.4, 122.0, 121.8, 119.2, 109.4, 43.5, 34.1, 31.4, 29.8, 29.7, 26.6, 22.5, 14.0; MS (*m*/*z*): 306; Anal. Calcd for C_21_H_26_N_2_: C, 82.31; H, 8.55; N, 9.14. Found: C, 82.15; H, 8.41; N, 8.98.

#### 3.3.13. 2-(3-Chlorophenyl)-1-hexyl-1H-benzo[*d*]imidazole (**22**)

From **7c** and 3-chlorobenzoyl chloride; Yield: 95 mg (31 mol, 68%) as a tan oil; IR: 1645, 1597, 1578, 1448, 1332, 1288 cm^−1^; ^1^H NMR (400 MHz, CDCl_3_): δ 7.82 (m, 1H), 7.74 (t, *J* = 1.8 Hz, 1H), 7.60 (dt, *J* = 7.1, 1.8 Hz, 1H), 7.51–7.45 (complex, 2H), 7.42 (m, 1H), 7.35–7.28 (complex, 2H), 4.22 (t, *J* = 7.7 Hz, 2H), 1.81 (pentet, *J* = 7.2 Hz, 2H), 1.31–1.18 (complex, 6H), 0.84 (distorted t, *J* = 6.7 Hz, 3H); ^13^C {^1^H} NMR (101 MHz, CDCl_3_): δ 152.1, 143.0, 135.6, 134.8, 132.5, 130.0, 129.8, 129.5, 127.4, 123.0, 122.6, 120.1, 110.2, 44.8, 31.1, 29.7, 26.3, 22.5, 13.9; MS (*m*/*z*): 312; Anal. Calcd for C_19_H_21_ClN_2_: C, 81.05; H, 6.35; N, 8.95. Found: C, 80.94; H, 6.28; N, 8.79.

#### 3.3.14. 1-(3-Isopropoxypropyl)-2-methyl-1H-benzo[*d*]imidazole (**23**)

From **7d** and acetyl chloride; Yield: 70 mg (0.30 mmol, 72%) as a tan oil; IR: 1612, 1505, 1488, 1396, 1355, 1320, 1273, 1121, 1081 cm^−1^; ^1^H NMR (400 MHz, CDCl_3_): δ 7.68 (m, 1H), 7.34 (m, 1H), 7.21 (m, 2H), 4.21 (m, 2H), 3.55 (m, 1H), 3.32 (t, *J* = 7.1 Hz, 2H), 2.61 (s, 3H), 2.03 (pentet, *J* = 6.1 Hz, 2H), 1.16 (d, *J* = 6.2 Hz, 6H); ^13^C {^1^H} NMR (101 MHz, CDCl_3_): δ 151.8, 142.6, 135.2, 121.8, 121.7, 118.9, 109.3, 71.7, 63.8, 40.5, 30.0, 22.1, 13.7; MS (*m*/*z*): 232; Anal. Calcd for C_14_H_20_N_2_O: C, 72.38; H, 8.68; N, 12.06. Found: C, 72.08; H, 8.53; N: 11.96.

#### 3.3.15. 1-(3-Isopropoxypropyl)-2-phenyl-1H-benzo[*d*]imidazole (**24**)

From **7d** and benzoyl chloride; Yield: 85 mg (0.29 mmol, 70%) as a tan oil; IR: 1616, 1518, 1478, 1389, 1250, 1150, 1082 cm^−1^; ^1^H NMR (400 MHz, CDCl_3_): δ 7.83 (m, 1H), 7.76 (m, 2H), 7.53–7.45 (complex, 4H), 7.30 (m, 2H), 4.39 (m, 2H), 3.37 (septet, *J* = 6.1 Hz, 1H), 3.29 (t, *J* = 5.7 Hz, 2H), 2.02 (m, 2H), 1.06 (d, *J* = 6.1 Hz, 6H); ^13^C {^1^H} NMR (101 MHz, CDCl_3_): δ 153.7, 143.0, 135.8, 130.5, 129.7, 129.4, 128.7, 122.7, 122.4, 119.8, 110.3, 71.7, 64.3, 41.9, 30.4, 22.0; MS (*m*/*z*): 294; Anal. Calcd for C_19_H_22_N_2_O: C, 77.52; H, 7.53; N, 9.52. Found: C, 77.39; H, 7.37; N, 9.46.

#### 3.3.16. 2-(2-Fluorophenyl)-1-(3-isopropoxypropyl)-1H-benzo[*d*]imidazole (**25**)

From **7d** and 2-fluorobenzoyl chloride; Yield: 94 mg (0.30 mmol, 72%) as a tan oil; IR: 1617, 1585, 1448, 1391, 1250, 1152, 1086 cm^−1^; ^1^H NMR (400 MHz, CDCl_3_): δ 7.84 (m, 1H), 7.65 (td, *J* = 7.4, 1.9 Hz, 1H), 7.52–7.46 (complex, 2H), 7.34–7.26 (complex, 3H), 7.22 (ddd, *J* = 9.9, 2.8, 1.4 Hz, 1H), 4.26 (t, *J* = 7.0 Hz, 2H), 3.32 (septet, *J* = 6.1 Hz, 1H), 3.21 (t, *J* = 5.8 Hz, 2H), 1.94 (m, 2H), 1.01 (d, *J* = 6.1 Hz, 6H); ^13^C {^1^H} NMR (101 MHz, CDCl_3_): δ 160.1 (d, *J* = 249.4 Hz), 148.9, 143.3, 135.4, 132.4 (d, *J* = 2.6 Hz), 132.0 (d, *J* = 8.2 Hz), 124.7 (d, *J* = 3.6 Hz), 122.9, 121.3, 120.1, 119.0 (d, *J* = 15.0 Hz), 116.1 (d, *J* = 21.6 Hz), 110.4, 71.5, 64.2, 41.7 (d, *J* = 3.6 Hz), 30.0 (d, *J* = 1.2 Hz), 21.9; ^19^F {^1^H} NMR (376 MHz, CDCl_3_): δ −112.9; MS (*m*/*z*): 312; Anal. Calcd for C_19_H_21_FN_2_O: C, 73.05; H, 6.78; N, 8.97. Found: C, 72.91; H, 6.67; N: 8.86.

#### 3.3.17. 2-(4-Fluoro-3-methylphenyl)-1-(3-isopropoxypropyl)-1H-benzo[*d*]imidazole (**26**)

From **7d** and 4-fluoro-3-methylbenzoyl chloride; Yield: 101 mg (0.31 mmol, 74%) as a tan oil; IR: 1609, 1594, 1520, 1452, 1394, 1240, 1155, 1084 cm^−1^; ^1^H NMR (400 MHz, CDCl_3_): δ 7.81 (m, 1H), 7.48 (m, 1H), 7.36 (dd, *J* = 8.4, 5.7 Hz, 1H), 7.35–7.26 (complex, 2H), 7.05 (dd, *J* = 9.7, 2.7 Hz, 1H), 7.00 (td, *J* = 8.3, 2.7 Hz, 1H), 4.12 (t, *J* = 7.2 Hz, 2H), 3.37 (septet, *J* = 6.1 Hz, 1H), 3.25 (t, *J* = 5.8 Hz, 2H), 2.24 (s, 3H), 1.88 (pentet, *J* = 6.1 Hz, 2H), 1.05 (d, *J* = 6.1 Hz, 6H); ^13^C {^1^H} NMR (101 MHz, CDCl_3_): δ 163.4 (d, *J* = 249.1 Hz), 152.3, 143.0, 140.9 (d, *J* = 8.4 Hz), 134.9, 131.9 (d, *J* = 8.8 Hz), 126.4 (d, *J* = 3.2 Hz), 122.6, 122.2, 119.9, 117.3 (d, *J* = 21.4 Hz), 112.9 (d, *J* = 21.6 Hz), 110.2, 71.5, 64.2, 41.3, 30.3, 22.0, 19.9 (d, *J* = 1.7 Hz); ^19^F {^1^H} NMR (376 MHz, CDCl_3_): δ −111.7; MS (*m*/*z*): 326; Anal. Calcd for C_20_H_23_FN_2_O: C, 73.59; H, 7.10; N, 8.58. Found: C, 73.66; H, 7.13; N: 8.49.

#### 3.3.18. 1-Benzyl-2-methyl-1H-benzo[*d*]imidazole (**27**)

From **7e** and acetyl chloride; Yield: 76 mg (0.34 mmol, 78%) as a tan oil; IR: 1660, 1598, 1512, 1448, 1397, 1345, 1316, 1281 cm^−1^; ^1^H NMR (400 MHz, CDCl_3_): δ 7.73 (dt, *J* = 7.6, 0.9 Hz, 1H), 7.31–7.15 (complex, 6H), 7.02 (br d, *J* = 7.3 Hz, 2H), 5.25 (s, 2H), 2.53 (s, 3H); ^13^C {^1^H} NMR (101 MHz, CDCl_3_): δ 151.9, 142.7, 135.9, 135.5, 129.0, 127.9, 126.2, 122.3, 122.0, 119.2, 109.4, 40.1, 14.0; MS (*m*/*z*): 222. The NMR spectral data matched those previously reported [44].

#### 3.3.19. 1-Benzyl-2-phenyl-1H-benzo[*d*]imidazole (**28**)

From **7e** and benzoyl chloride; Yield: 90 mg (0.32 mmol, 72%) as a tan solid, m.p. 131–133 °C; IR: 1605, 1570, 1503, 1446, 1402, 1378, 1335, 1177 cm^−1^; ^1^H NMR (400 MHz, CDCl_3_): δ 7.87 (d, *J* = 8.1 Hz, 1H), 7.69 (m, 2H), 7.50–7.41 (complex, 3H), 7.35–7.28 (complex, 4H), 7.24–7.19 (complex, 2H), 7.10 (m, 2H), 5.45 (s, 2H); ^13^C {^1^H} NMR (101 MHz, CDCl_3_): δ 154.2, 143.2, 136.4, 136.1, 130.1, 129.9, 129.3, 129.1, 128.8, 127.8, 126.0, 123.1, 122.7, 120.0, 110.6, 48.4; MS (*m*/*z*): 284. The NMR spectral data matched those previously reported [19].

#### 3.3.20. 1-Benzyl-2-(4-methoxyphenyl)-1H-benzo[*d*]imidazole (**29**)

From **7e** and 4-methoxybenzoyl chloride; Yield: 99 mg (0.32 mmol, 72%) as a tan solid, m.p. 133–135 °C; IR: 2842, 1606, 1587, 1530, 1467, 1302, 1262, 1186, 848 cm^−1^; ^1^H NMR (400 MHz, CDCl_3_): δ 7.85 (dt, *J* = 8.0, 1.0 Hz, 1H), 7.63 (d, *J* = 8.8 Hz, 2H), 7.36–7.27 (complex, 4H), 7.24–7.17 (complex, 2H), 7.11 (d, *J* = 8.8 Hz, 2H), 6.96 (d, *J* = 8.8 Hz, 2H), 5.44 (s, 2H), 3.84 (s, 3H); ^13^C {^1^H} NMR (101 MHz, CDCl_3_): δ 160.9, 154.2, 143.2, 136.6, 136.1, 130.7, 129.1, 127.8, 126.0, 122.8, 122.6, 122.4, 119.8, 114.2, 110.4, 55.4, 48.4; MS (*m*/*z*): 314. The NMR spectral data matched those previously reported [19].

#### 3.3.21. 1-Benzyl-2-(4-(trifluoromethyl)phenyl)-1H-benzo[*d*]imidazole (**30**)

From **7e** and 4-(trifluoromethyl)benzoyl chloride; Yield: 108 mg (0.31 mmol, 70%) as an off-white solid, m.p. 118–120 °C (lit [43] m.p. 107–109 °C); IR: 1620, 1592, 1497, 1450, 1324, 1175, 1112, 845 cm^−1^; ^1^H NMR (400 MHz, CDCl_3_): δ 7.89 (d, *J* = 7.9 Hz, 1H), 7.82 (d, *J* = 8.2 Hz, 2H), 7.71 (d, *J* = 8.2 Hz, 2H), 7.38–7.30 (complex, 4H), 7.30–7.24 (complex, 2H), 7.10 (br d, *J* = 7.2 Hz, 2H), 5.47 (s, 2H); ^13^C {^1^H} NMR (101 MHz, CDCl_3_): δ 152.5, 143.1, 136.2, 136.0, 133.6, 131.8 (q, *J* = 32.7 Hz), 129.6, 129.2, 128.0, 125.8, 125.7 (q, *J* = 3.8 Hz), 123.8 (q, *J* = 272.3 Hz), 123.7, 123.1, 120.2, 110.6, 48.4.; ^19^F {^1^H} NMR (376 MHz, CDCl_3_): δ −62.6; MS (*m*/*z*): 352. The NMR spectral data matched those previously reported [45].

#### 3.3.22. 2-Methyl-1-phenethyl-1H-benzo[*d*]imidazole (**31**)

From **7f** and acetyl chloride; Yield: 76 mg (0.32 mmol, 77%) as an off-white solid, m.p. 43–44 °C; IR: 1655, 1598, 1524, 1465, 1398, 1365, 1340, 1291 cm^−1^; ^1^H NMR (400 MHz, CDCl_3_): δ 7.69 (m, 1H), 7.28–7.17 (complex, 6H), 6.92 (m, 2H), 4.23 (m, 2H), 3.01 (m, 2H), 2.13 (d, *J* = 1.9 Hz, 3H); ^13^C {^1^H} NMR (101 MHz, CDCl_3_): δ 151.8, 142.6, 137.7, 134.6, 128.8, 128.7, 127.0, 122.0, 121.8, 119.1, 109.1, 45.5, 35.7, 13.3; MS (*m*/*z*): 236; Anal. Calcd for C_16_H_16_N_2:_ C, 81.32; H, 6.82; N, 11.85. Found: C, 81.02; H, 7.01; N, 11.86.

#### 3.3.23. 2-Pentyl-1-phenethyl-1H-benzo[*d*]imidazole (**32**)

From **7f** and hexanoyl chloride; Yield: 88 mg (0.30 mmol, 73%) as a tan solid, m.p. 43–45 °C; IR: 1662, 1599, 1524, 1463, 1397 cm^−1^; ^1^H NMR (400 MHz, CDCl_3_): δ 7.74 (m, 1H), 7.32–7.17 (complex, 6H), 6.99 (m, 2H), 4.30 (t, *J* = 7.1 Hz, 2H), 3.06 (t, *J* = 7.1 Hz, 2H), 2.45 (t, *J* = 7.8 Hz, 2H), 1.73 (m, 2H), 1.35–1.29 (complex, 4H), 0.89 (distorted t, *J* = 6.8 Hz, 3H); ^13^C {^1^H} NMR (101 MHz, CDCl_3_): δ 155.4, 142.8, 137.8, 134.7, 128.84, 128.78, 127.0, 122.0, 121.8, 119.3, 109.1, 45.3, 36.0, 31.8, 27.2, 27.1, 22.4, 14.0; MS (*m*/*z*): 292; Anal. Calcd for C_20_H_24_N_2_: C, 82.15; H, 8.27; N, 9.58. Found: C, 82.04; H, 8.23; N, 9.46.

#### 3.3.24. 2-Isopropyl-1-phenethyl-1H-benzo[*d*]imidazole (**33**)

From **7f** and isobutyryl chloride; Yield: 82 mg (0.31 mmol, 75%) as a tan oil; IR: 1620, 1498, 1460, 1412 cm^−1^; ^1^H NMR (400 MHz, CDCl_3_): δ 7.77 (m 1H), 7.34 (m, 1H), 7.29–7.20 (complex, 5H), 6.99 (m, 2H), 4.34 (t, *J* = 7.1 Hz, 2H), 3.08 (t, *J* = 7.1 Hz, 2H), 2.14 (septet, *J* = 6.8 Hz, 1H), 1.26 (d, *J* = 6.8 Hz, 6H); ^13^C {^1^H} NMR (101 MHz, CDCl_3_): δ 160.0, 142.7, 137.7, 134.5, 128.9, 128.8, 127.0, 122.0, 121.8, 119.5, 109.3, 45.0, 36.0, 26.3, 21.6; MS (*m*/*z*): 264; Anal. Calcd for C_18_H_20_N_2_: C, 81.78; H, 7.63; N, 10.60. Found: C, 81.63; H, 7.61; N, 10.46.

#### 3.3.25. 2-(4-Chlorophenyl)-1-phenethyl-1H-benzo[*d*]imidazole (**34**)

From **7f** and 4-chlorobenzoyl chloride; Yield: 94 mg (0.28 mmol, 68%) as a tan solid, m.p. 89–91 °C; IR: 1620, 1592, 1483, 1409, 1172, 1122, 1096, 838 cm^−1^; ^1^H NMR (400 MHz, CDCl_3_): δ 7.83 (m, 1H), 7.47 (m, 1H), 7.39 (d, *J* = 8.8 Hz, 2H), 7.36–7.30 (m, 2H), 7.32 (d, *J* = 8.8 Hz, 2H), 7.21–7.16 (complex, 3H), 6.87–6.83 (complex, 2H), 4.45 (t, *J* = 7.2 Hz, 2H), 3.08 (t, *J* = 7.2 Hz, 2H); ^13^C {^1^H} NMR (101 MHz, CDCl_3_): δ 152.8, 143.1, 137.2, 135.8, 135.2, 130.5, 128.80, 128.78, 128.6, 127.0, 123.1, 122.7, 120.2, 110.2, 46.2, 35.7 (1 aromatic C unresolved); MS (*m*/*z*): 332; Anal. Calcd for C_21_H_17_ClN_2_: C, 75.78; H, 5.15; N, 8.42. Found: C, 75.52; H, 5.14; N, 8.35.

#### 3.3.26. 2-Isopropyl-1-(4-methoxybenzyl)-1H-benzo[*d*]imidazole (**35**)

From **7g** and isobutyryl chloride; Yield: 78 mg (0.28 mmol, 72%) as a tan oil; IR: 2838, 1605, 1511, 1468, 1410, 1294, 1247, 1188, 1090, 1022 cm^−1^; ^1^H NMR (400 MHz, CDCl_3_): δ 7.78 (dt, *J* = 7.9, 1.0 Hz, 1H), 7.25–7.14 (complex, 3H), 6.96 (d, *J* = 8.8 Hz, 2H), 6.81 (d, *J* = 8.8 Hz, 2H), 5.29 (s, 2H), 3.74 (s, 3H), 3.15 (septet, *J* = 6.8 Hz, 1H), 1.39 (d, *J* = 6.8 Hz, 6H); ^13^C {^1^H} NMR (101 MHz, CDCl_3_): δ 160.1, 159.2, 142.7, 135.3, 128.2, 127.3, 122.2, 121.9, 119.4, 114.3, 109.6, 55.3, 46.2, 26.6, 21.7; MS (*m*/*z*): 280; Anal. Calcd for C_18_H_20_N_2_O: C, 77.11; H, 7.19; N, 9.99. Found: C, 76.95; H, 7.11; N, 9.92.

#### 3.3.27. 1-(4-Methoxybenzyl)-2-phenyl-1H-benzo[*d*]imidazole (**36**)

From **7g** and benzoyl chloride; Yield: 84 mg (0.27 mmol, 70%) as an off-white solid, m.p. 138–140 °C; IR: 2842, 1609, 1508, 1468, 1447, 1398, 1259, 1187, 1024 cm^−1^; ^1^H NMR (400 MHz, CDCl_3_): δ 7.86 (d, *J* = 8.0 Hz, 1H), 7.69 (m, 2H), 7.49–7.40 (complex, 3H), 7.29 (m, 1H), 7.22 (m, 2H), 7.00 (d, *J* = 8.5 Hz, 2H), 6.83 (d, *J* = 8.5 Hz, 2H), 5.37 (s, 2H), 3.76 (s, 3H); ^13^C {^1^H} NMR (101 MHz, CDCl_3_): δ 159.2, 154.1, 143.2, 136.1, 130.2, 129.9, 129.3, 128.8, 128.4, 127.3, 123.0, 122.7, 120.0, 114.4, 110.6, 55.3, 47.9; MS (*m*/*z*): 314; Anal. Calcd for C_21_H_18_N_2_O: C, 80.23; H, 5.77; N, 8.91. Found: C, 79.93; H, 5.95; N, 8.75.

#### 3.3.28. 2-(2-Fluorophenyl)-1-(4-methoxyphenyl)-1H-benzo[*d*]imidazole (**37**)

From **7g** and 2-fluorobenzoyl chloride; Yield: 93 mg (0.28 mmol, 73%) as a tan oil; IR: 2842, 1614, 1512, 1466, 1256, 1190, 1122, 1032 cm^−1^; ^1^H NMR (400 MHz, CDCl_3_): δ 7.85 (dt, *J* = 8.0, 1.1 Hz, 1H), 7.60 (td, *J* = 7.4, 1.8 Hz, 1H), 7.46 (m, 1H), 7.32–7.17 (complex, 5H), 6.93 (d, *J* = 8.7 Hz, 2H), 6.75 (d, *J* = 8.7 Hz, 2H), 5.26 (s, 2H), 3.72 (s, 3H); ^13^C {^1^H} NMR (101 MHz, CDCl_3_): δ 160.2 (d, *J* = 247.7 Hz), 159.1, 149.2, 143.4, 135.3, 132.5 (d, *J* = 2.4 Hz), 132.1 (d, *J* = 8.2 Hz), 127.9, 124.7 (d, *J* = 3.6 Hz), 123.1, 122.5, 120.1, 118.7 (d, *J* = 14.9 Hz), 116.2 (d, *J* = 21.4 Hz), 114.1, 110.9, 55.2, 48.0 (d, *J* = 3.5 Hz) (1 aromatic C unresolved); ^19^F {^1^H} NMR (376 MHz, CDCl_3_): δ −112.8; MS (*m*/*z*): 332; Anal. Calcd for C_21_H_17_FN_2_O: C, 75.89; H, 5.16; N, 8.43. Found: C, 75.73; H, 5.09; N, 8.26.

#### 3.3.29. 2-(4-Fluoro-3-methylphenyl)-1-(4-methoxybenzyl)-1H-benzo[*d*]imidazole (**38**)

From **7g** and 4-fluoro-3-methylbenzoyl chloride; Yield: 100 mg (0.29 mmol, 75%) as a light yellow solid, m.p. 100–101 °C; IR: 2843, 1617, 1509, 1477, 1423, 1328, 1185, 1119, 1080, 833 cm^−1^; ^1^H NMR (400 MHz, CDCl_3_): δ 7.85 (dt, *J* = 8.0, 1.1 Hz, 1H), 7.60 (dd, *J* = 7.5, 2.3 Hz, 1H), 7.42 (ddd, *J* = 7.9, 5.1, 2.3 Hz, 1H), 7.32 (m, 1H), 7.24 (m, 2H), 7.06 (t, *J* = 8.9 Hz, 1H), 7.02 (d, *J* = 8.6 Hz, 2H), 6.85 (d, *J* = 8.6 Hz, 2H), 5.37 (s, 2H), 3.78 (s, 3H), 2.30 (d, *J* = 2.0 Hz, 3H); ^13^C {^1^H} NMR (101 MHz, CDCl_3_): δ 162.3 (d, *J* = 249.2 Hz), 159.2, 153.4, 143.1, 136.1, 132.9 (d, *J* = 5.7 Hz), 128.4, 128.2 (d, *J* = 8.6 Hz), 127.2, 126.0 (d, *J* = 3.7 Hz), 125.7 (d, *J* = 17.8 Hz), 123.0, 122.7, 119.9, 115.3 (d, *J* = 23.0 Hz), 114.5, 110.5, 55.3, 47.9, 14.5 (d, *J* = 3.5 Hz); ^19^F {^1^H} NMR (376 MHz, CDCl_3_): δ −114.9; MS (*m*/*z*): 346; Anal. Calcd for C_22_H_19_FN_2_O: C, 76.28; H, 5.53; N, 8.09. Found: C, 76.23; H, 5.49; N, 7.97.

#### 3.3.30. 2-Isopropyl-1-(4-(trifluoromethyl)benzyl)-1H-benzo[*d*]imidazole (**39**)

From **7h** and isobutyryl chloride; Yield: 74 mg (0.23 mmol, 69%) as a tan oil; IR: 1613, 1512, 1476, 1422, 1330, 1181, 1127, 1072, 838 cm^−1^; ^1^H NMR (400 MHz, CDCl_3_): δ 7.82 (dt, *J* = 8.0, 1.0 Hz, 1H), 7.56 (d, *J* = 8.2 Hz, 2H), 7.26 (td, *J* = 8.4, 7.3, 1.3 Hz, 1H), 7.16 (ddd, *J* = 8.2, 7.3, 1.2 Hz, 1H), 7.12 (br d, *J* = 8.1 Hz, 3H), 5.42 (s, 2H), 3.12 (septet, *J* = 6.8 Hz, 1H), 1.40 (d, *J* = 6.8 Hz, 6H); ^13^C {^1^H} NMR (101 MHz, CDCl_3_): δ 160.0, 142.7, 140.3 (q, *J* = 1.5 Hz), 135.0, 130.3 (q, *J* = 32.6 Hz), 126.3, 126.0 (q, *J* = 3.8 Hz), 123.9 (q, *J* = 272.2 Hz), 122.6, 122.3, 119.6, 109.3, 46.2, 26.6, 21.7; ^19^F {^1^H} NMR (376 MHz, CDCl_3_): δ −62.7; MS (*m*/*z*): 318; Anal. Calcd for C_18_H_17_F_3_N_2_: C, 67.91; H, 5.38; N, 8.80. Found: C, 67.81; H, 5.40; N: 8.71.

#### 3.3.31. 2-(4-Methoxyphenyl)-1-(4-trifluoromethyl)benzyl)-1H-benzo[*d*]imidazole (**40**)

From **7h** and 4-methoxybenzoyl chloride; Yield: 93 mg (0.24 mmol, 72%) as a tan oil; IR: 2834, 1610, 1498, 1470, 1321, 1252, 1174, 1119, 1077 cm^−1^; ^1^H NMR (400 MHz, CDCl_3_): δ 7.87 (dt, *J* = 7.9, 1.0 Hz, 1H), 7.61 (d, *J* = 8.5 Hz, 2H and d, *J* = 8.8 Hz, 2H), 7.32 (ddd, *J* = 8.2, 7.1, 1.2 Hz, 1H), 7.24 (overlapping d, *J* = 8.5 Hz, 2H and m, 1H), 7.14 (d, *J* = 7.8 Hz, 1H), 6.97 (d, *J* = 8.8 Hz, 2H), 5.49 (s, 2H), 3.85 (s, 3H); ^13^C {^1^H} NMR (101 MHz, CDCl_3_): δ 161.1, 154.1, 143.2, 140.6 (q, *J* = 1.1 Hz), 135.9, 130.6, 130.2 (q, *J* = 32.7 Hz), 126.3, 126.1 (q, *J* = 3.8 Hz), 123.9 (q, *J* = 272.2 Hz), 123.1, 122.9, 122.1, 120.0, 114.4, 110.1, 55.4, 48.0; ^19^F {^1^H} NMR (376 MHz, CDCl_3_): δ −62.7; MS (*m*/*z*): 382; Anal. Calcd for C_22_H_17_F_3_N_2_O: C, 69.10; H, 4.48; N, 7.33. Found: C, 68.94; H, 4.33; N, 7.25.

#### 3.3.32. 2-(3-Chlorophenyl)-1-(4-(trifluoromethyl)benzyl)-1H-benzo[*d*]imidazole (**41**)

From **7h** and 3-chlorobenzoyl chloride; Yield: 91 mg (0.24 mmol, 70%) as a tan solid, m.p. 95–97 °C; IR: 1608, 1574, 1476, 1323, 1227, 1170, 1128, 1079 cm^−1^; ^1^H NMR (400 MHz, CDCl_3_): δ 7.89 (d, *J* = 8.0 Hz, 1H), 7.71 (t, *J* = 1.9 Hz, 1H), 7.61 (d, *J* = 8.1 Hz, 2H), 7.47 (m, 2H), 7.39–7.36 (complex, 2H), 7.26 (td, *J* = 8.1, 1,2 Hz, 1H), 7.22–7.17 (complex, 3H), 5.50 (s, 2H); ^13^C {^1^H} NMR (101 MHz, CDCl_3_): δ 152.5, 143.1, 140.1, 135.9, 135.1, 131.6, 130.4 (q, *J* = 32.7 Hz), 130.2, 130.1, 129.5, 127.0, 126.3, 126.2 (q, *J* = 3.7 Hz), 123.8 (q, *J* = 272.3 Hz), 123.7, 123.2, 120.4, 110.3, 48.0; ^19^F {^1^H} NMR (376 MHz, CDCl_3_): δ −62.7; MS (*m*/*z*): 386; Anal. Calcd for C_21_H_14_ F_3_ClN_2_: C, 65.21; H, 3.65; N, 7.24. Found: C, 65.06; H, 3.52; N, 7.18.

#### 3.3.33. 2-(2-Fluorophenyl)-1-(4-(trifluoromethyl)benzyl)-1H-benzo[*d*]imidazole (**42**)

From **7h** and 2-fluorobenzoyl chloride; Yield: 93 mg (0.25 mmol, 74%) as a tan oil; IR: 1606, 1480, 1326, 1228, 1180, 1132, 1089 cm^−1^; ^1^H NMR (400 MHz, CDCl_3_): δ 7.88 (d, *J* = 8.0 Hz, 1H), 7.62 (td, *J* = 7.4, 1.8 Hz, 1H), 7.51 (overlapping d, *J* = 8.0 Hz, 2H and m, 1H), 7.32 (t, *J* = 7.4 Hz, 1H), 7.30–7.12 (complex, 4H), 7.12 (d, *J* = 8.2 Hz, 2H), 5.38 (s, 2H); ^13^C {^1^H} NMR (101 MHz, CDCl_3_): δ 160.2 (d, *J* = 249.6 Hz), 149.3, 143.5, 139.9, 135.2, 132.5 (d, *J* = 2.4 Hz), 132.4 (d, *J* = 8.2 Hz), 130.1 (q, *J* = 32.6 Hz), 127.5, 125.8 (q, *J* = 3.8 Hz), 124.9 (d, *J* = 3.5 Hz), 123.9 (q, *J* = 272.1 Hz), 123.5, 122.8, 120.4, 118.3 (d, *J* = 14.8 Hz), 116.2 (d, *J* = 21.5 Hz), 110.5, 48.0 (d, *J* = 3.7 Hz); ^19^F {^1^H} NMR (376 MHz, CDCl_3_): δ −62.7, −112.8; MS (*m*/*z*): 370; Anal. Calcd for C_21_H_14_ F_4_N_2_: C, 68.11; H, 3.81; N, 7.56. Found: C, 67.97; H, 3.78; N, 7.42.

#### 3.3.34. 2-Methyl-1-phenyl-1H-benzo[*d*]imidazole (**43**)

From **7i**; Ten equiv of acetyl chloride was used and the reaction was heated at 95–100 °C for 1 week; Yield: 86 mg (0.40 mmol, 86%) as a tan solid, m.p. 48–50 °C (lit. [13] m.p. 49–51 °C); IR: 1618, 1598, 1497, 1464, 1395, 1370, 1324, 1283, 1244, 1018 cm^−1^; ^1^H NMR (400 MHz, CDCl_3_): δ 7.75 (dd, *J* = 8.0, 1.1 Hz, 1H), 7.61–7.48 (complex, 3H), 7.36 (m, 2H), 7.26 (m, 1H), 7.19 (tt, *J* = 8.2, 1.5 Hz, 1H), 7.12 (d, *J* = 8.2 Hz, 1H), 2.51 (br s, 3H); ^13^C {^1^H} NMR (101 MHz, CDCl_3_): δ 151.5, 142.6, 136.5, 136.1, 129.9, 128.8, 127.1, 122.6, 122.4, 119.0, 109.9, 14.1; MS (*m*/*z*): 208. These spectral data matched those previously reported [13].

#### 3.3.35. 1-Phenyl-2-(4-(trifluoromethyl)phenyl)-1H-benzo[*d*]imidazole (**44**)

From **7i**; Five equiv of 4-(trifluoromethyl)benzoyl chloride was used and the reaction was heated at 95–100 °C for 1 week; Yield: 81 mg (0.29 mmol, 51%) as a yellow solid, m.p. 127–129 °C; IR: 1612, 1597, 1497, 1330, 1175, 1110, 1078 cm^−1^; ^1^H NMR (400 MHz, CDCl_3_): δ 7.91 (d, *J* = 8.0 Hz, 1H), 7.70 (d, *J* = 8.2 Hz, 2H), 7.57–7.50 (complex, 5H), 7.39–7.25 (complex, 5H); ^13^C {^1^H} NMR (101 MHz, CDCl_3_): δ 150.7, 142.9, 137.4, 136.6, 133.5, 131.2 (q, *J* = 32.6 Hz), 130.1, 129.7, 129.0, 127.4, 125.3 (q, *J* = 3.8 Hz), 124.0, 123.9 (q, *J* = 272.3 Hz), 123.4, 120.2, 110.7; ^19^F {^1^H} NMR (376 MHz, CDCl_3_): δ −62.9; MS (*m*/*z*): 338. The NMR spectral data matched those previously reported [46].

#### 3.3.36. 1-(4-Methoxyphenyl)-2-methyl-1H-benzo[*d*]imidazole (**45**)

From **7j**; Ten equiv of acetyl chloride was used and the reaction was heated at 95–100 °C for 1 week; Yield: 76 mg (0.32 mmol, 78%) as a tan solid, m.p. 98–100 °C; IR: 2838, 1606, 1581, 1512, 1458, 1395, 1322, 1250, 1082, 1004, 827 cm^−1^; ^1^H NMR (400 MHz, CDCl_3_): δ 7.73 (d, *J* = 7.9 Hz, 1H), 7.28 (overlapping d, *J* = 8.9 Hz, 2H and m, 1H), 7.18 (td, *J* = 8.1, 1.2 Hz, 1H), 7.08 (overlapping m, 1H and d, *J* = 8.9 Hz, 2H), 3.90 (s, 3H), 2.48 (s, 3H); ^13^C {^1^H} NMR (101 MHz, CDCl_3_): δ 159.7, 151.9, 142.5, 136.9, 128.7, 128.3, 122.4, 122.2, 118.9, 115.0, 109.9, 55.6, 14.3; MS (*m*/*z*): 238; Anal. Calcd for C_15_H_14_N_2_O: C, 75.61; H, 5.92; N, 11.76. Found: C, 75.64; H, 5.86; N, 11.66.

#### 3.3.37. 1-(4-Methoxyphenyl)-2-phenyl-1H-benzo[*d*]imidazole (**46**)

From **7j**; Five equiv of benzoyl chloride was used, and the reaction was heated at 95–100 °C for 1 week; Yield: 68 mg (0.23 mmol, 55%) as a tan solid, m.p. 118–120 °C [lit. [39] m.p. 119–120 °C]; IR: 2840, 1609, 1575, 1508, 1439, 1375, 1251 cm^−1^; ^1^H NMR (400 MHz, CDCl_3_): δ 7.88 (dt, *J* = 7.9, 1.0 Hz, 1H), 7.60 (m, 2H), 7.37–7.27 (complex, 4H), 7.23 (overlapping m, 2H and d, *J* = 8.9 Hz, 2H), 7.00 (d, *J* = 8.9 Hz, 2H), 3.87 (s, 3H); ^13^C {^1^H} NMR (101 MHz, CDCl_3_): δ 159.5, 157.5, 142.9, 137.6, 130.1, 129.7, 129.43, 129.38, 128.6, 128.3, 123.2, 122.9, 119.8, 115.0, 110.5, 55.6; MS (*m*/*z*): 300. The NMR spectral data matched those previously reported [47].

## 4. Conclusions

The current study describes a convenient approach to the synthesis of 1,2-disubstituted benzimidazoles. The sequence consists of three steps: (1) S_N_Ar addition of an amine to 2-fluoro-1-nitrobenzene to give an *N*-alkyl- or *N*-aryl-2-nitroaniline (70–80% yields); (2) acylation of the aniline nitrogen to generate an *N*-alkyl- or *N*-aryl-*N*-(2-nitrophenyl) amide; and (3) a one-pot reduction of nitro using iron in acetic acid, followed by attack of the resulting amine at the amide carbonyl and dehydration to give the target heterocycles. Steps 2 and 3 proceeded in good to excellent overall yields (62–88%) for *N*-alkyl-2-nitroanilines. The acylation (Step 2) of the *N*-alkyl substrates was sluggish due to the conjugated nitro function at C2 of the adjacent aromatic ring and required forcing conditions (*ca* 100 °C, 24 h) for complete amide formation. This same step was particularly difficult for the *N*-aryl derivatives (*ca* 100 °C, 1 week), and yields were lower due to steric and electronic factors that impeded approach to the amine nitrogen and further decreased its nucleophilicity. This is a weakness of the current procedure. The acylated benzimidazole precursors were not characterized as they consisted of a mixture of C–N rotamers that resulted in broad and/or doubled peaks in the ^1^H and ^13^C NMR spectra. Attempts to simplify the ^1^H NMR spectra at temperatures up to 100 °C failed to coalesce the signals. Finally, reductive cyclization of the *N*-alkyl-*N*-(2-nitrophenyl) amides proceeded in nearly quantitative yields to the benzimidazoles, which gave clean NMRs and acceptable elemental analyses without further purification. Interestingly, the final cyclization produced only the benzimidazoles by closure of the nitro-derived aniline nitrogen on the amide carbonyl with no competing acyl transfer.

## Figures and Tables

**Table 1:** Synthesis of *N*-alkyl or *N*-aryl-2-nitroaniline substrates.

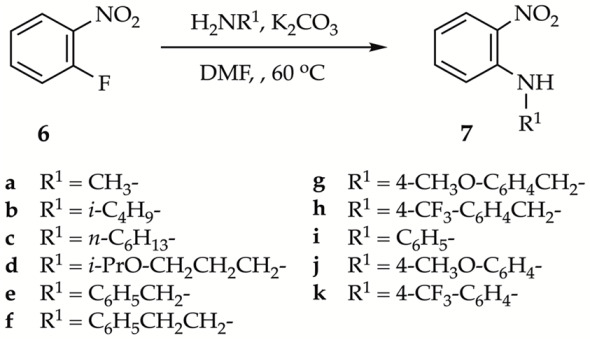		
Compound	R^1^	Yield (%)
**7a** ^a^	CH_3_-	42 ^b^
**7b** ^a^	*i*-C_4_H_9_-	72
**7c** ^a^	*n*-C_6_H_13_-	74
**7d**	*i*-PrO-CH_2_CH_2_CH_2_-	82
**7e** ^a^	C_6_H_5_CH_2_-	81
**7f** ^a^	C_6_H_5_CH_2_CH_2_-	80
**7g** ^a^	4-CH_3_O-C_6_H_4_CH_2_-	78
**7h**	4-CF_3_-C_6_H_4_CH_2_-	71
**7i** ^a^	C_6_H_5_-	60
**7j** ^a^	4-CH_3_O-C_6_H_4_-	55
**7k**	4-CF_3_-C_6_H_4_-	0

^a^ The m.p. and/or NMR are reported in the literature. ^b^ 2 equiv of 40% methanolic CH_3_NH_2_ was used, and *ca* 25% of 1-methoxy-2-nitrobenzene was also produced.

**Table 2:** Acylation and reductive ring closure of benzimidazoles.

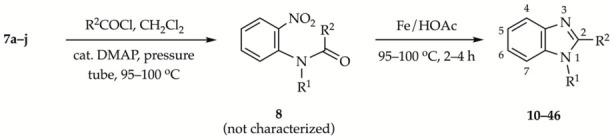				
Substrate	R^1^	R^2^	Compound Number	Benzimidazole Yield (%)
**7a**	CH_3_-	CH_3_-	**10** ^a^	88
	CH_3_-	C_6_H_5_-	**11** ^a^	85
	CH_3_-	C_6_H_5_CH_2_CH_2_-	**12** ^a^	85
	CH_3_-	4-CH_3_O-C_6_H_4_-	**13**	79
	CH_3_-	4-Cl-C_6_H_4_-	**14** ^a^	78
**7b**	*i*-C_4_H_9_-	CH_3_-	**15**	76
	*i*-C_4_H_9_-	C_6_H_5_-	**16**	72
	*i*-C_4_H_9_-	4-CH_3_O-C_6_H_4_-	**17**	77
	*i*-C_4_H_9_-	4-CF_3_-C_6_H_4_-	**18**	62
**7c**	*n*-C_6_H_13_-	CH_3_-	**19**	73
	*n*-C_6_H_13_-	C_6_H_5_-	**20** ^a^	71
	*n*-C_6_H_13_-	C_6_H_5_CH_2_CH_2_-	**21**	72
	*n*-C_6_H_13_-	3-Cl-C_6_H_4_-	**22**	68
**7d**	*i*-PrO-CH_2_CH_2_CH_2_-	CH_3_-	**23**	72
	*i*-PrO-CH_2_CH_2_CH_2_-	C_6_H_5_-	**24**	70
	*i*-PrO-CH_2_CH_2_CH_2_-	2-F-C_6_H_4_-	**25**	72
	*i*-PrO-CH_2_CH_2_CH_2_-	4-F-3-CH_3_-C_6_H_3_-	**26**	74
**7e**	C_6_H_5_CH_2_-	CH_3_-	**27** ^a^	78
	C_6_H_5_CH_2_-	C_6_H_5_-	**28** ^a^	72
	C_6_H_5_CH_2_-	4-CH_3_O-C_6_H_4_-	**29** ^a^	72
	C_6_H_5_CH_2_-	4-CF_3_-C_6_H_4_-	**30** ^a^	70
**7f**	C_6_H_5_CH_2_CH_2_-	CH_3_-	**31**	77
	C_6_H_5_CH_2_CH_2_-	*n*-C_5_H_11_-	**32**	73
	C_6_H_5_CH_2_CH_2_-	*i*-C_3_H_7_-	**33**	75
	C_6_H_5_CH_2_CH_2_-	4-Cl-C_6_H_4_-	**34**	68
**7g**	4-CH_3_O-C_6_H_4_CH_2_-	*i*-C_3_H_7_-	**35**	72
	4-CH_3_O-C_6_H_4_CH_2_-	C_6_H_5_-	**36**	70
	4-CH_3_O-C_6_H_4_CH_2_-	2-F-C_6_H_4_-	**37**	73
	4-CH_3_O-C_6_H_4_CH_2_-	4-F-3-CH_3_-C_6_H_3_-	**38**	75
**7h**	4-CF_3_-C_6_H_4_CH_2_-	*i*-C_3_H_7_-	**39**	69
	4-CF_3_-C_6_H_4_CH_2_-	4-CH_3_O-C_6_H_4_-	**40**	72
	4-CF_3_-C_6_H_4_CH_2_-	3-Cl-C_6_H_4_-	**41**	70
	4-CF_3_-C_6_H_4_CH_2_-	2-F-C_6_H_4_-	**42**	74
**7i**	C_6_H_5_-	CH_3_-	**43** ^a^	86 ^b^
	C_6_H_5_-	4-CF_3_-C_6_H_4_-	**44** ^a^	51 ^c^
**7j**	4-CH_3_O-C_6_H_4_-	CH_3_-	**45**	78 ^b^
	4-CH_3_O-C_6_H_4_-	C_6_H_5_-	**46** ^a^	55 ^c^

^a^ The m.p. and/or NMR are reported in the literature. ^b^ 10 equiv of the acid chloride was used, and the reaction was heated at 95–100 °C for 1 week. ^c^ 5 equiv of the acid chloride was used, and the reaction was heated at 95–100 °C for 1 week.

## Data Availability

The original contributions presented in this study are included in the article/Supplementary Material. Further inquiries can be directed to the corresponding author.

## Supplementary Materials

The following supporting information can be downloaded at: https://www.mdpi.com/article/10.3390/molecules31122150/s1. Copies of ^1^H NMR and ^13^C NMR spectra for all substrates and 1*H*-indole products are provided. ^19^F NMR spectra are provided for compounds that incorporate fluorine.
