# Supplementary material for: Reductive Cyclization of N-Alkyl- and N-Aryl-N-(2-Nitrophenyl) Amides to 1,2-Disubstituted Benzimidazoles
Source: Molecules. 2026 Jun 18;31(12):2150. doi: 10.3390/molecules31122150 (PMC13304832; doi:10.3390/molecules31122150)

Reductive Cyclization of *N*-Alkyl- and *N*-Aryl- *N*-(2-Nitrophenyl) Amides to 1,2-Disubstituted Benzimidazoles

Nash E. Nevels, Matthew E. Germond and Richard. A. Bunce *

Department of Chemistry, Oklahoma State University, Stillwater, OK 74078-3071, USA
nnevels@okstate.edu (N.E.N); matthew.germond@okstate.edu (M.E.G.)
* Correspondence: richard.a.bunce@okstate.edu; Tel.: +1-405-744-5952

Supplemental Information

Compound **Page**

^{1}H and ^{13}C spectra for *N*-Methyl-2-nitroaniline (**7a**) 4
^{1}H and ^{13}C spectra for 1,2-Dimethyl-1*H*-benzo[*d*]imidazole (**10**) 5
^{1}H and ^{13}C spectra for 1-Methyl-2-phenyl-1*H*-benzo[*d*]imidazole (**11**) 6
^{1}H and ^{13}C spectra for 1-Methyl-2-phenethyl-1*H*-benzo[*d*]imidazole (**12**) 7
^{1}H and ^{13}C spectra for 2-(4-Methoxyphenyl)-1-methyl-1*H*-benzo[*d*]imidazole (**13**) 8
^{1}H and ^{13}C spectra for 2-(3-Chlorophenyl)-1-methyl-1*H*-benzo[*d*]imidazole (**14**) 9

^{1}H and ^{13}C spectra for *N*-Isobutyl-2-nitroaniline (**7b**) 10
^{1}H and ^{13}C spectra for 1-Isobutyl-2-methyl-1*H*-benzo[*d*]imidazole (**15**) 11
^{1}H and ^{13}C spectra for 1-Isobutyl-2-phenyl-1*H*-benzo[*d*]imidazole (**16**) 12
^{1}H and ^{13}C spectra for 1-Isobutyl-2-(4-methoxyphenyl)-1*H*-benzo[*d*]imidazole (**17**) 13
^{1}H and ^{13}C spectra for 1-Isobutyl-2-(4-(trifluoromethyl)phenyl)-1*H*-benzo[*d*]imidazole (**18**) 14
^{19}F spectrum for 1-Isobutyl-2-(4-(trifluoromethyl)phenyl)-1*H*-benzo[*d*]imidazole (**18**) 15

^{1}H and ^{13}C spectra for *N*-Hexyl-2-nitroaniline (**7c**) 16
^{1}H and ^{13}C spectra for 1-Hexyl-2-isopropyl-1*H*-benzo[*d*]imidazole (**19**) 17
^{1}H and ^{13}C spectra for 1-Hexyl-2-phenyl-1*H*-benzo[*d*]imidazole (**20**) 18
^{1}H and ^{13}C spectra for 1-Hexyl-2-phenethyl-1*H*-benzo[*d*]imidazole (**21**) 19
^{1}H and ^{13}C spectra for 2-(3-Chlorophenyl)-1-hexyl-1*H*-benzo[*d*]imidazole (**22**) 20

^{1}H and ^{13}C spectra for *N*-(3-Isopropoxypropyl)-2-nitroaniline (**7d**) 21
^{1}H and ^{13}C spectra for 1-(3-Isopropoxypropyl)-2-methyl-1*H*-benzo[*d*]imidazole (**23**) 22
^{1}H and ^{13}C spectra for 1-(3-Isopropoxypropyl)-2-phenyl-1*H*-benzo[*d*]imidazole (**24**) 23
^{1}H and ^{13}C spectra for 1-(2-Fluorophenyl)-1-(3-isopropoxypropyl)-1*H*-benzo[*d*]imidazole (**25**) 24
^{19}F spectrum for 1-(2-Fluorophenyl)-1-(3-isopropoxypropyl)-1*H*-benzo[*d*]imidazole (**25**) 25

^{1}H and ^{13}C spectra for 2-(4-Fluoro-3-methylphenyl)-1-(3-isopropoxypropyl)-1*H*-benzo[*d*]-imidazole (**26**) 26
^{19}F spectrum for 2-(4-Fluoro-3-methylphenyl)-1-(3-isopropoxypropyl)-1*H*-benzo[*d*]imidazole (**26**) 27

^{1}H and ^{13}C spectra for *N*-Benzyl-2-nitroaniline (**7e**) 28
^{1}H and ^{13}C spectra for 1-Benzyl-2-methyl-1*H*-benzo[*d*]imidazole (**27**) 29
^{1}H and ^{13}C spectra for 1-Benzyl-2-phenyl-1*H*-benzo[*d*]imidazole (**28**) 30
^{1}H and ^{13}C spectra for 1-Benzyl-2-(4-methoxyphenyl)-1*H*-benzo[*d*]imidazole (**29**) 31
^{1}H and ^{13}C spectra for 1-Benzyl-2-(4-(trifluoromethyl)phenyl)-1*H*-benzo[*d*]imidazole (**30**) 32
^{19}F spectrum for 1-Benzyl-2-(4-(trifluoromethyl)phenyl)-1*H*-benzo[*d*]imidazole (**30**) 33

^{1}H and ^{13}C spectra for 2-Nitro-*N*-phenethylaniline (**7f**) 34
^{1}H and ^{13}C spectra for 2-Methyl-1-phenethyl-1H-benzo[d]imidazole (**31**) 35
^{1}H and ^{13}C spectra for 2-Pentyl-1-phenethyl-1*H*-benzo[*d*]imidazole (**32**) 36
^{1}H and ^{13}C spectra for 2-Isopropyl-1-phenethyl-1*H*-benzo[*d*]imidazole (**33**) 37
^{1}H and ^{13}C spectra for 2-(4-Chlorophenyl)-1-phenethyl-1*H*-benzo[*d*]imidazole (**34**) 38

^{1}H and ^{13}C spectra for *N*-(4-Methoxybenzyl)-2-nitroaniline (**7g**) 39
^{1}H and ^{13}C spectra for 2-Isopropyl-1-(4-methoxybenzyl)-1*H*-benzo[*d*]imidazole (**35**) 40
^{1}H and ^{13}C spectra for 1-(4-Methoxybenzyl)-2-phenyl-1*H*-benzo[*d*]imidazole (**36**) 41
^{1}H and ^{13}C spectra for 2-(2-Fluorophenyl)-1-(4-methoxyphenyl)-1*H*-benzo[*d*]imidazole (**37**) 42
^{19}F spectrum for 2-(2-Fluorophenyl)-1-(4-methoxyphenyl)-1*H*-benzo[*d*]imidazole (**37**) 43
^{1}H and ^{13}C spectra for 2-(4-Fluoro-3-methylphenyl)-1-(4-methoxybenzyl)-1*H*-benzo[*d*]-imidazole (**38**) 44
^{19}F spectrum for 2-(4-Fluoro-3-methylphenyl)-1-(4-methoxybenzyl)-1*H*-benzo[*d*]-imidazole (**38**) 45

^{1}H and ^{13}C spectra for 2-Nitro-*N*-(4-(trifluoromethyl)benzyl)aniline (**7h**) 46
^{19}F spectrum for 2-Nitro-*N*-(4-(trifluoromethyl)benzyl)aniline (**7h**)........ 47
^{1}H and ^{13}C spectra for 2-Isopropyl-1-(4-(trifluoromethyl)benzyl)-1*H*-benzo[*d*]imidazole (**39**) 48
^{19}F spectrum for 2-Isopropyl-1-(4-(trifluoromethyl)benzyl)-1*H*-benzo[*d*]imidazole (**39**) 49
^{1}H and ^{13}C spectra for 2-(4-Methoxyphenyl)-1-(4-trifluoromethyl)benzyl)-1*H*-benzo[*d*]-imidazole (**40**) 50
^{19}F spectrum for 2-(4-Methoxyphenyl)-1-(4-trifluoromethyl)benzyl)-1*H*-benzo[*d*]-imidazole (**40**) 51
^{1}H and ^{13}C spectra for 2-(3-Chlorophenyl)-1-(4-(trifluoromethyl)benzyl)-1*H*-benzo[*d*]-imidazole (**41**) 52
^{19}F spectrum for 2-(3-Chlorophenyl)-1-(4-(trifluoromethyl)benzyl)-1*H*-benzo[*d*]imidazole (**41**) 53
^{1}H and ^{13}C spectra for 2-(2-Fluorophenyl)-1-(4-(trifluoromethyl)benzyl)-1*H*-benzo[*d*]-imidazole (**42**) 54

^{19}F spectrum for 2-(2-Fluorophenyl)-1-(4-(trifluoromethyl)benzyl)-1*H*-benzo[*d*]imidazole (**42**) 55

^{1}H and ^{13}C spectra for 2-Nitro-*N*-phenylaniline (**7i**) 56
^{1}H and ^{13}C spectra for 2-Methyl-1-phenyl-1*H*-benzo[*d*]imidazole (**43**) 57
^{1}H and ^{13}C spectra for 1-Phenyl-2-(4-(trifluoromethyl)phenyl)-1*H*-benzo[*d*]imidazole (**44**)..... 58
^{19}F spectrum for 1-Phenyl-2-(4-(trifluoromethyl)phenyl)-1*H*-benzo[*d*]imidazole (**44**) 59

^{1}H and ^{13}C spectra for *N*-(4-Methoxyphenyl)-2-nitroaniline (**7j**) 60
^{1}H and ^{13}C spectra for 1-(4-Methoxyphenyl)-2-methyl-1*H*-benzo[*d*]imidazole (**45**) 61
^{1}H and ^{13}C spectra for 1-(4-Methoxyphenyl)-2-phenyl-1*H*-benzo[*d*]imidazole (**46**) 62

^{1}H and ^{13}C spectra for *N*-Methyl-2-nitroaniline (**7a**)

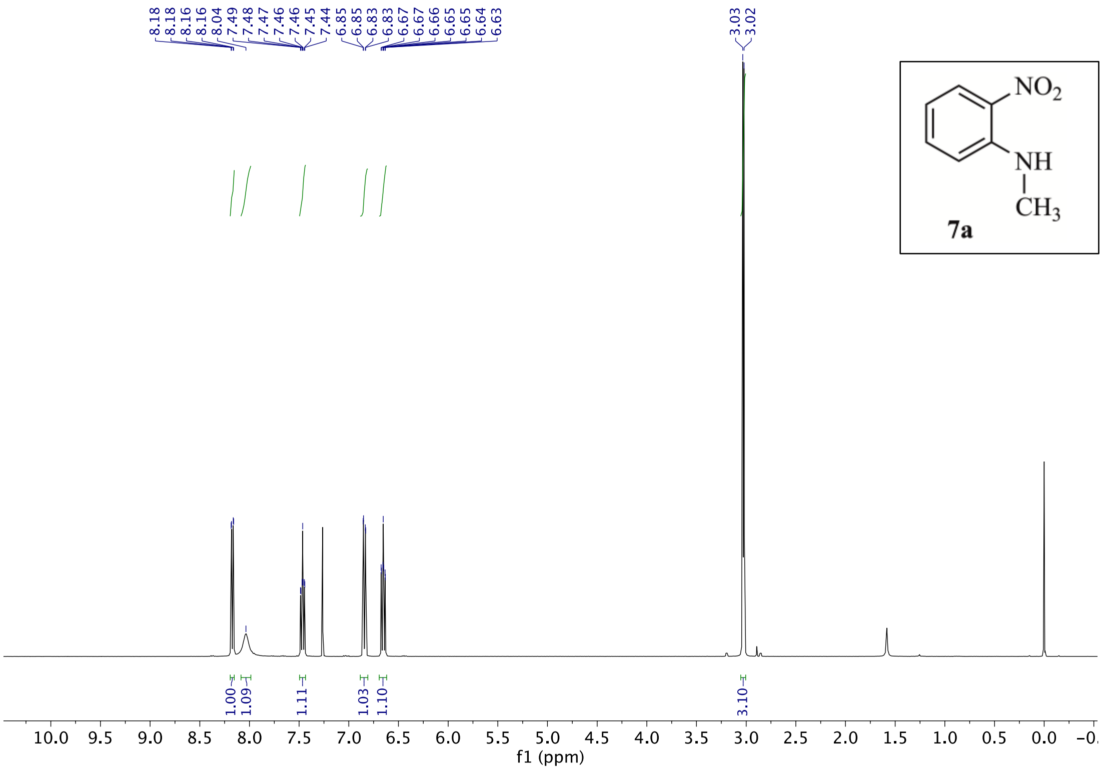

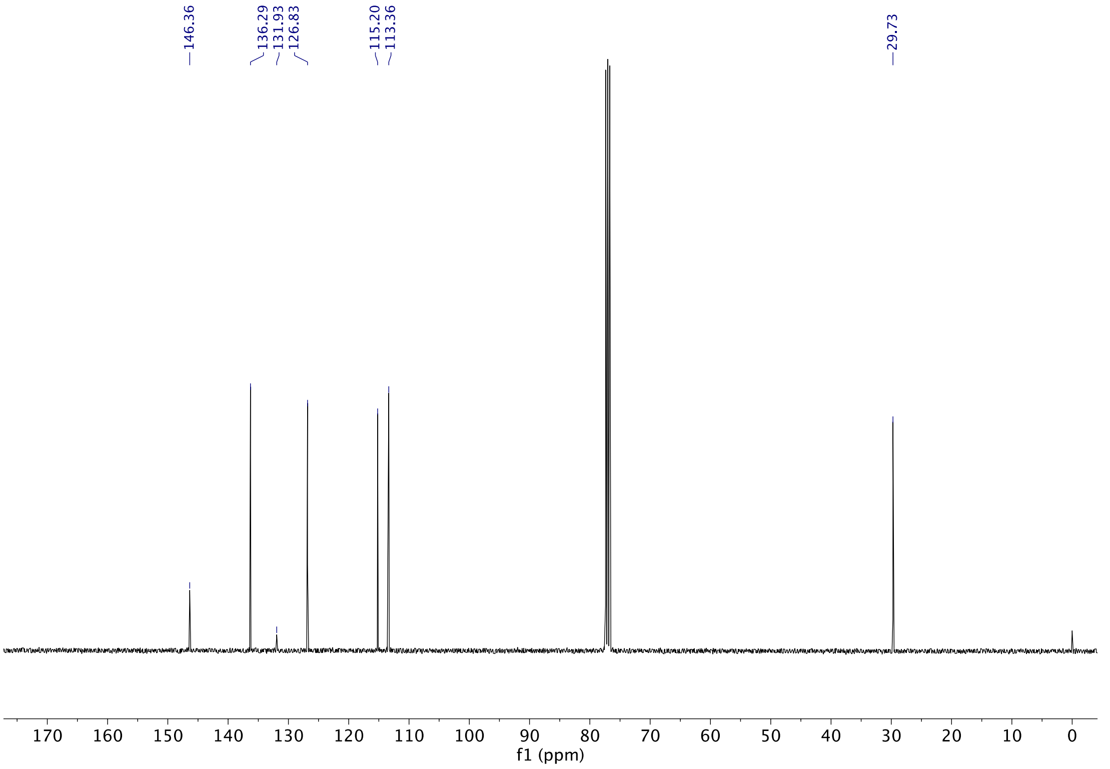

^{1}H and ^{13}C spectra for 1,2-Dimethyl-1*H*-benzo[*d*]imidazole (**10**)

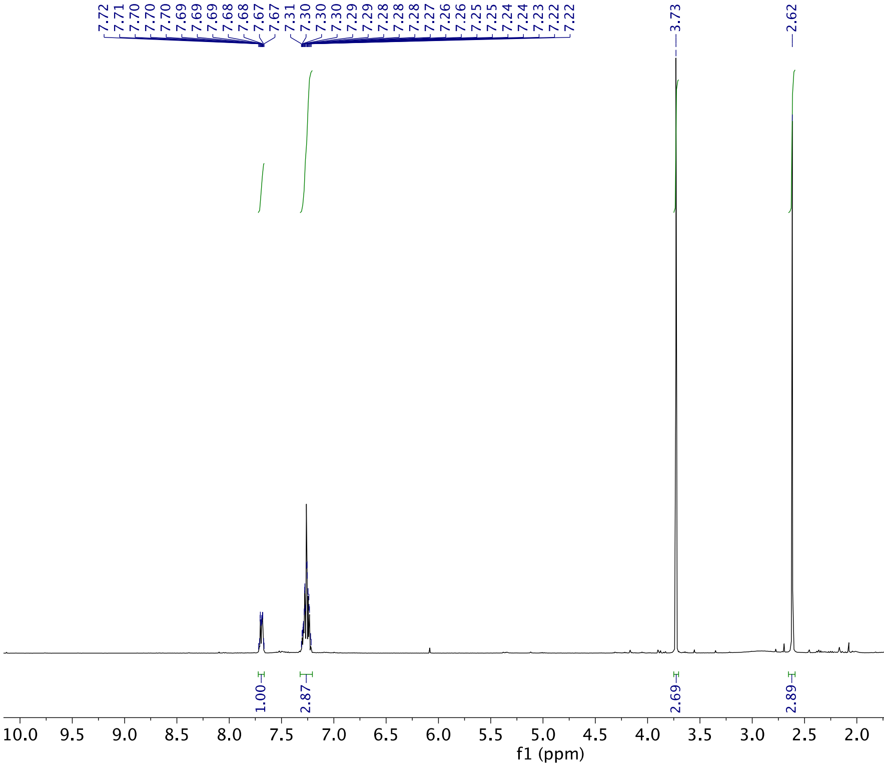

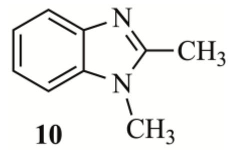

10

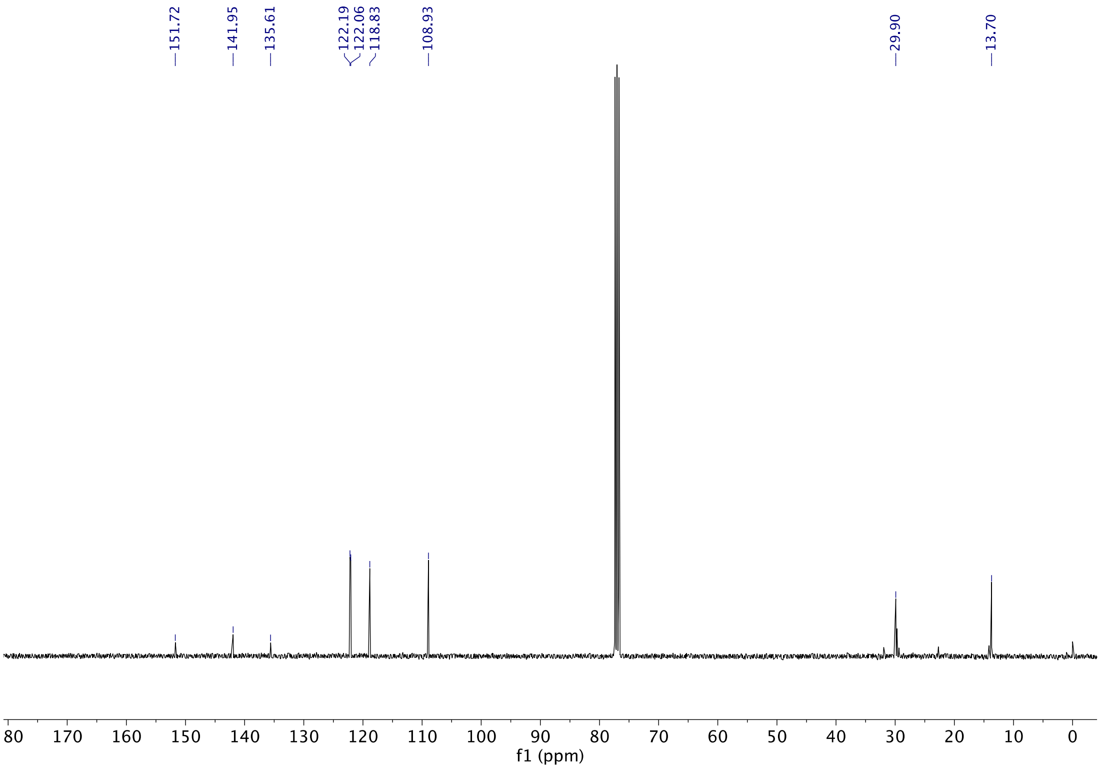

^{1}H and ^{13}C spectra for 1-Methyl-2-phenyl-1*H*-benzo[*d*]imidazole (**11**)

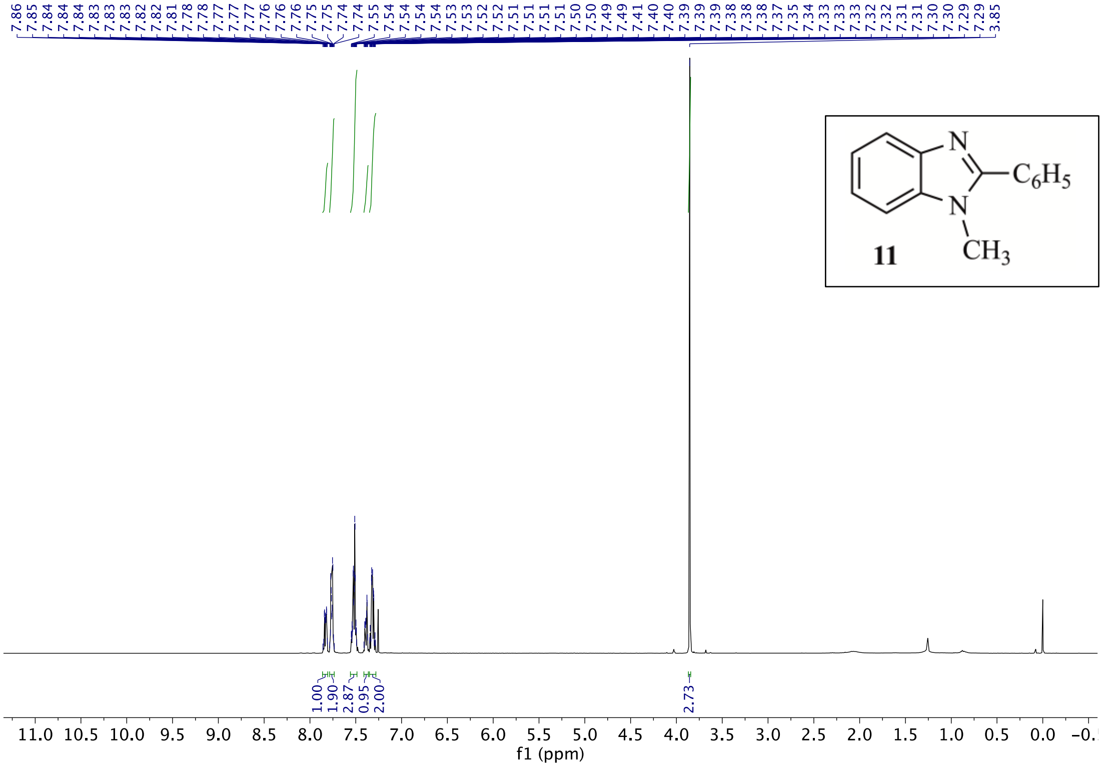

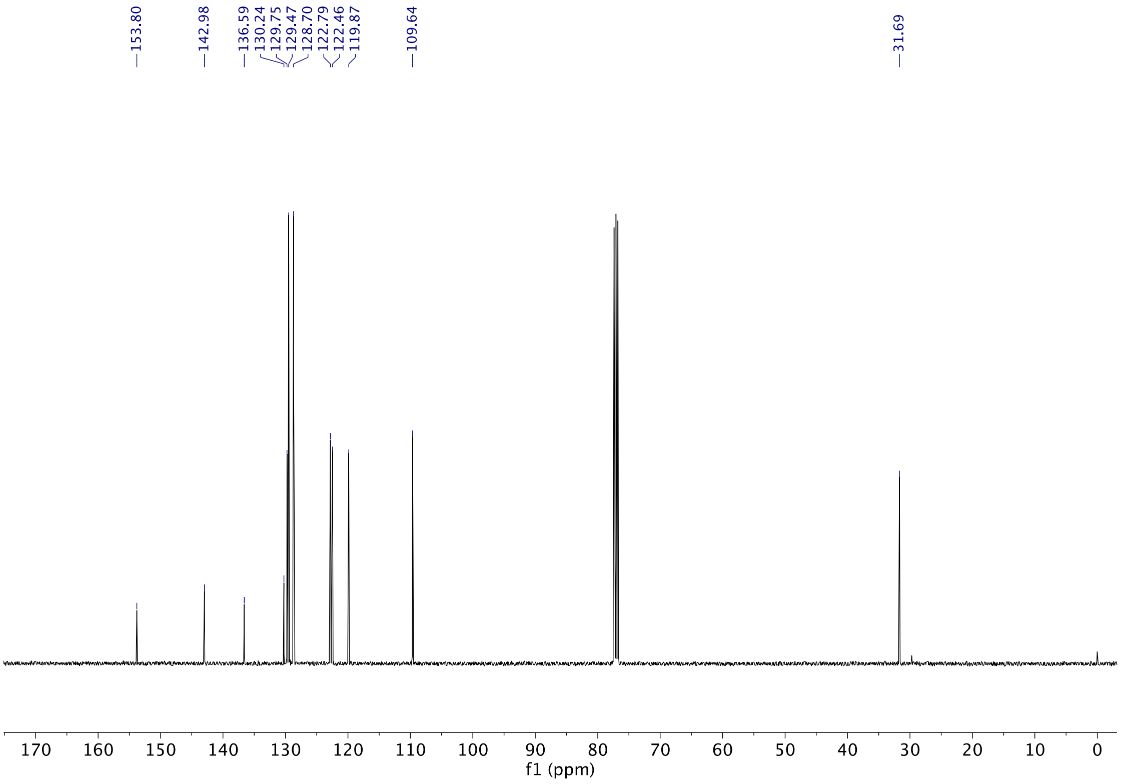

^{1}H and ^{13}C spectra for 1-Methyl-2-phenethyl-1*H*-benzo[*d*]imidazole (**12**)

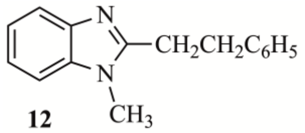

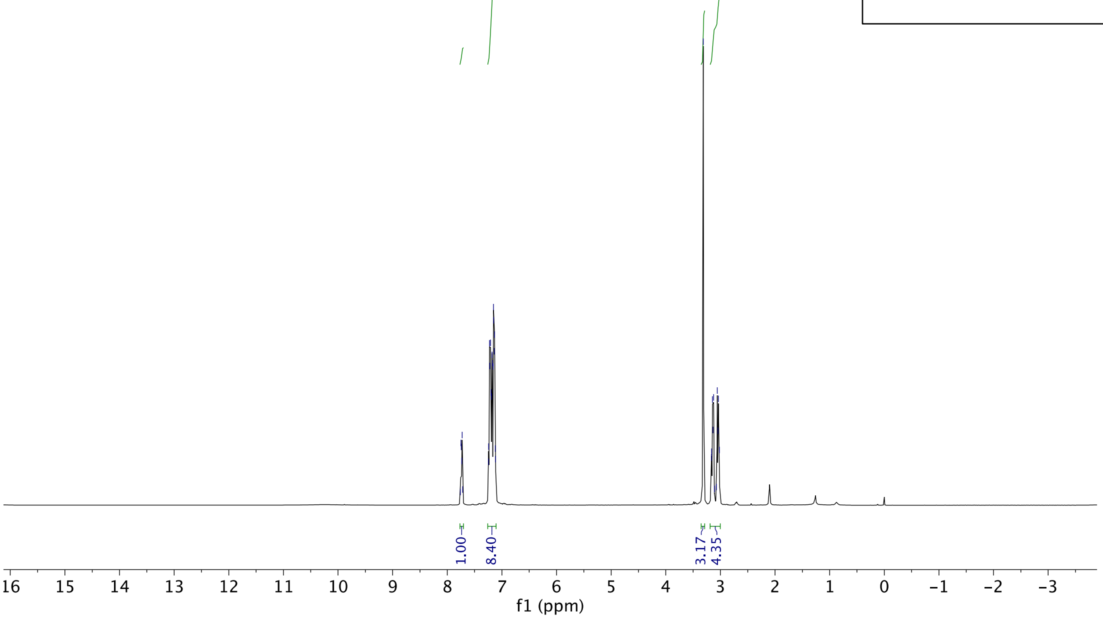

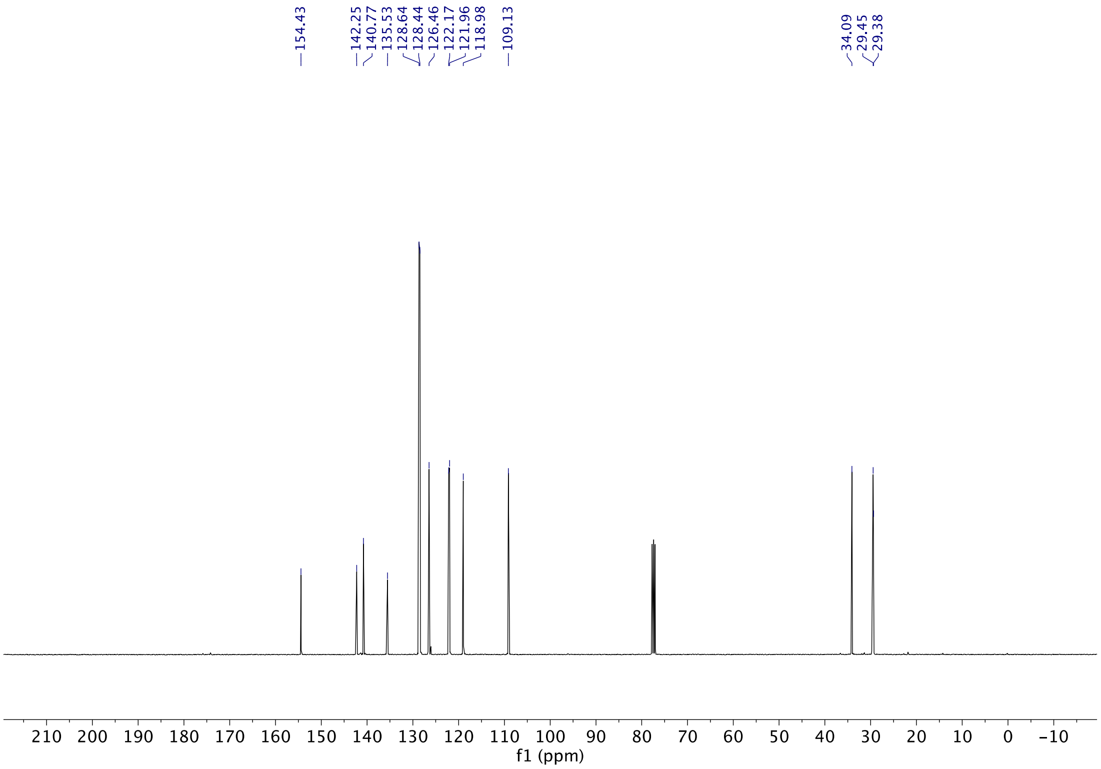

^{1}H and ^{13}C spectra for 2-(4-Methoxyphenyl)-1-methyl-1*H*-benzo[*d*]imidazole (**13**)

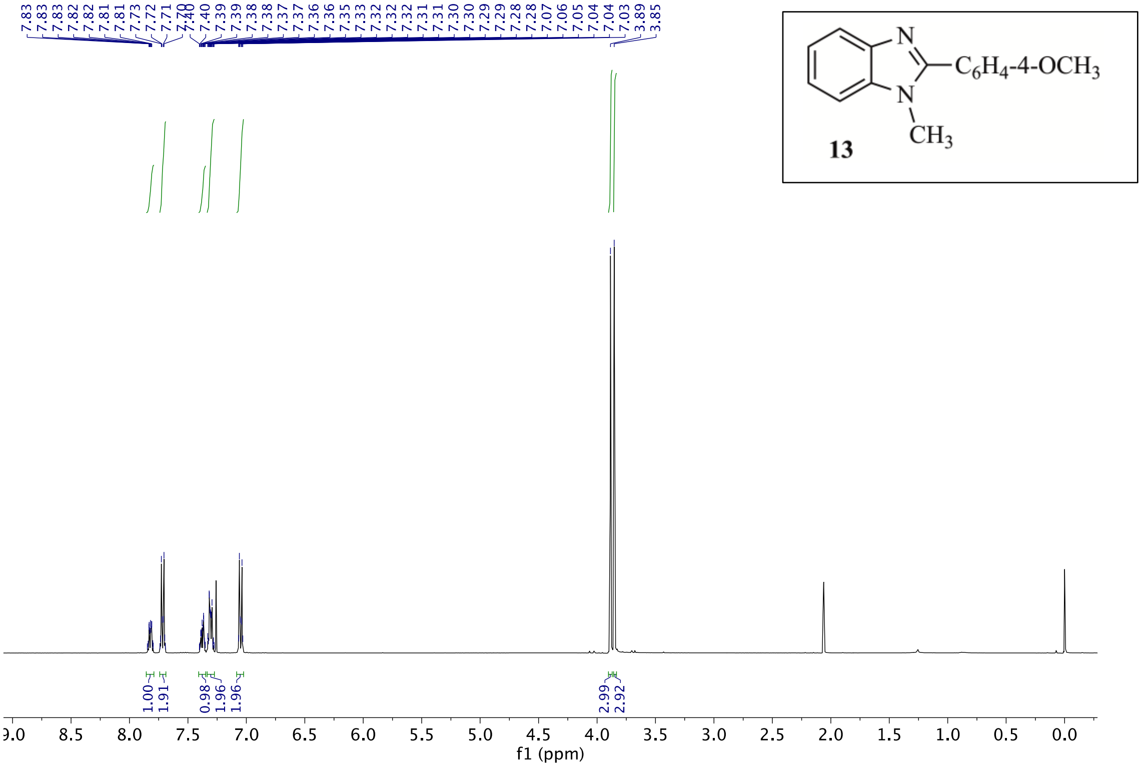

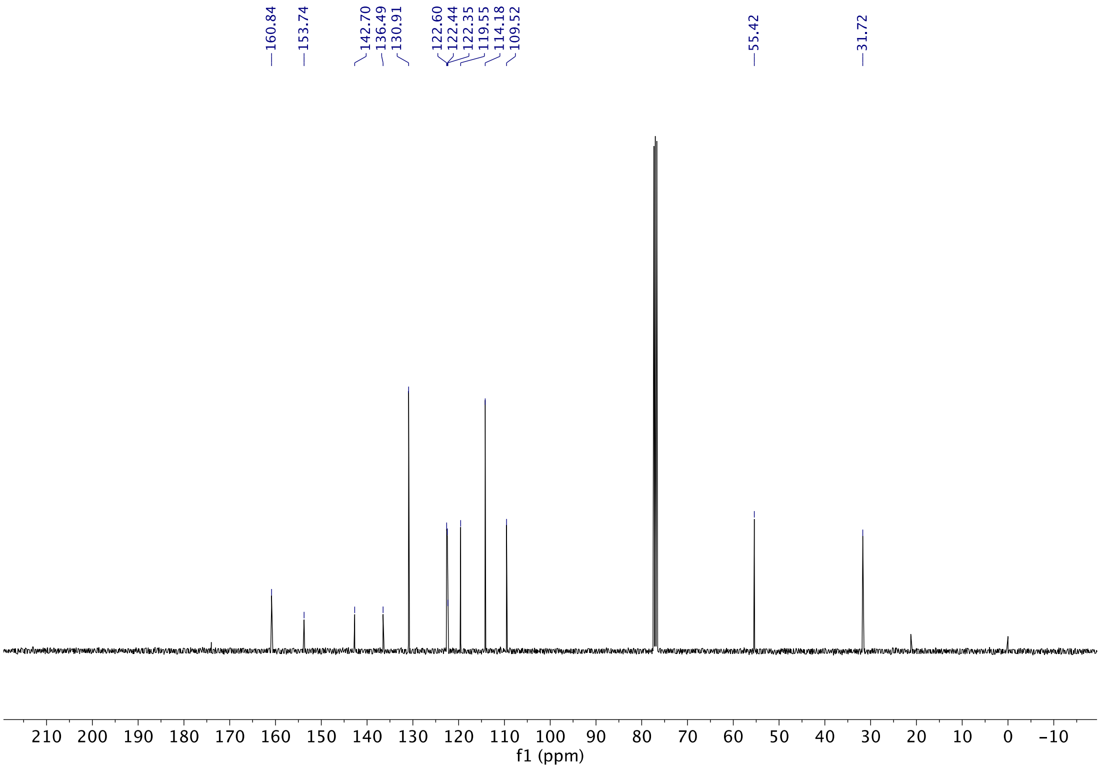

^{1}H and ^{13}C spectra for 2-(3-Chlorophenyl)-1-methyl-1*H*-benzo[*d*]imidazole (**14**)

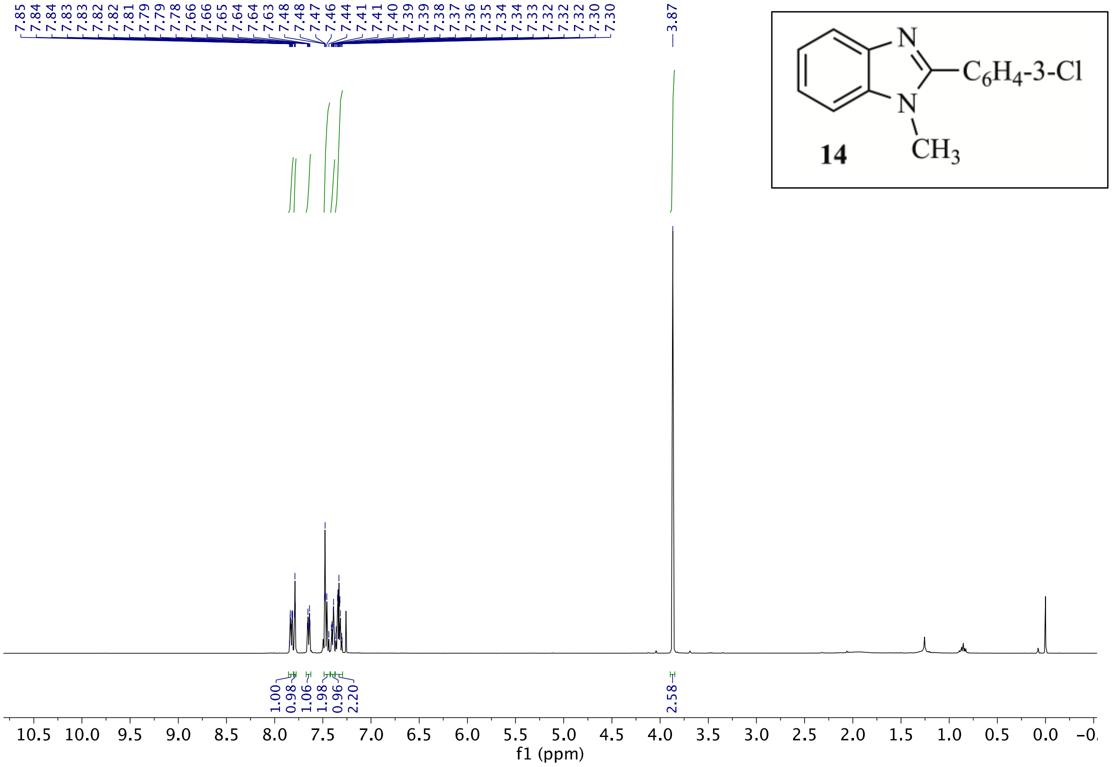

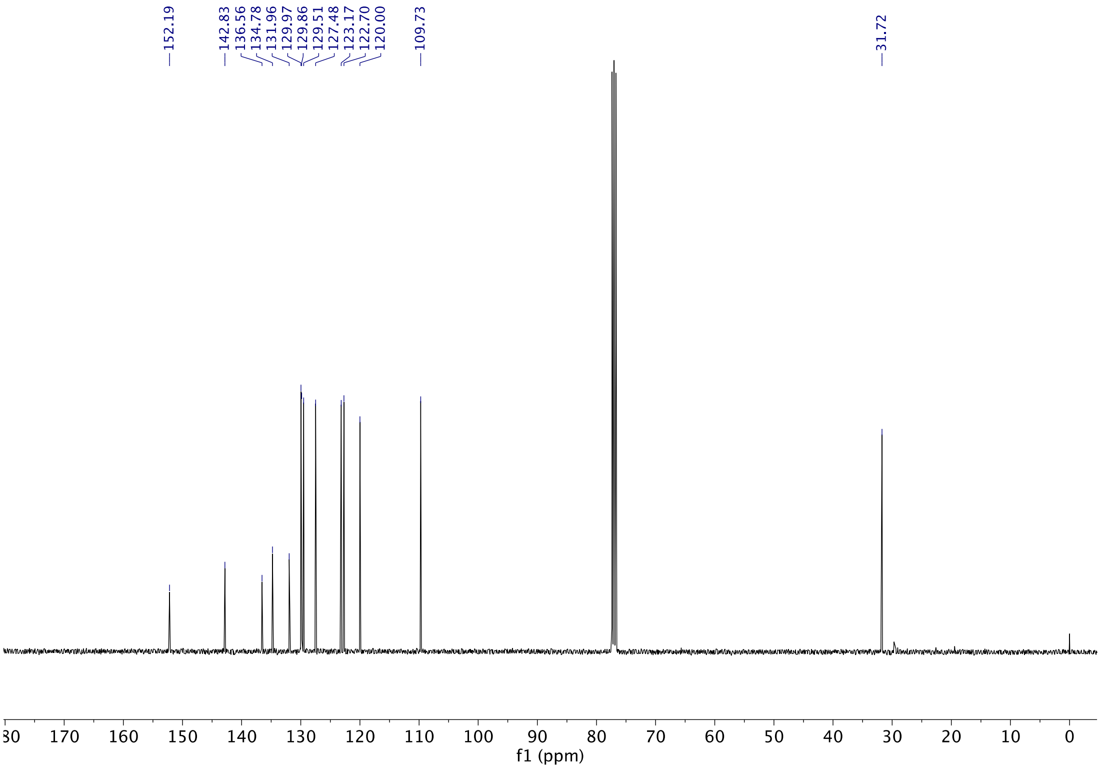

^{1}H and ^{13}C spectra for *N*-Isobutyl-2-nitroaniline (**7b**)

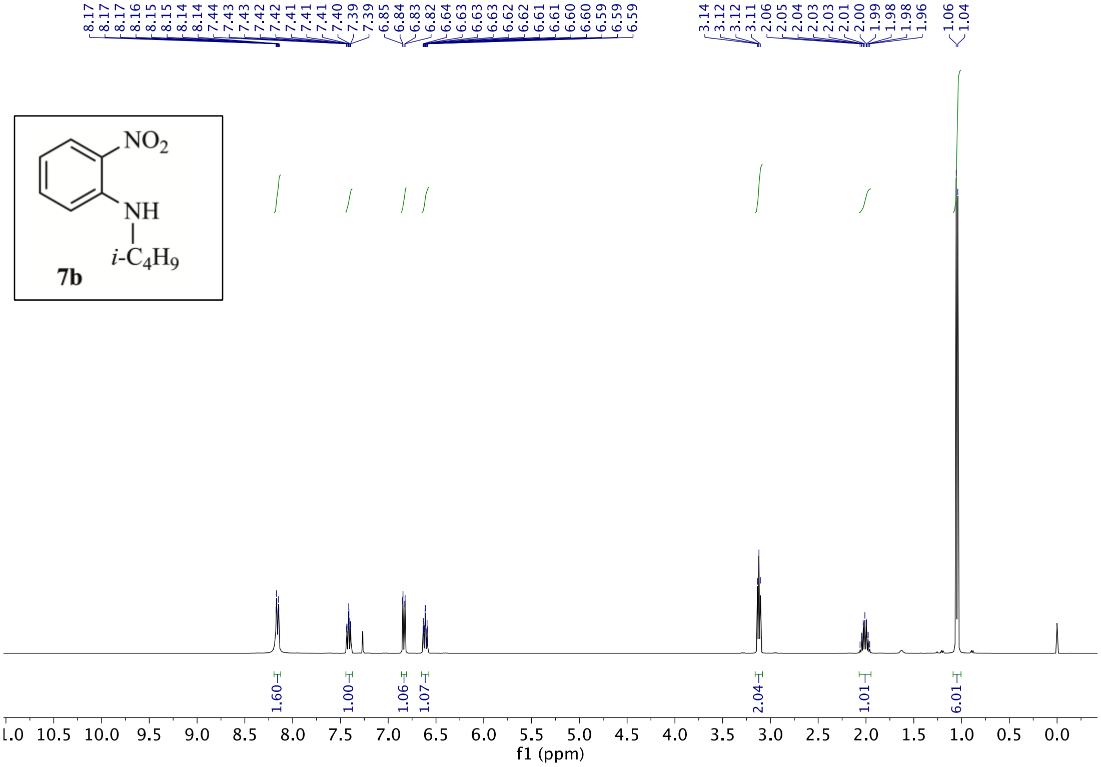

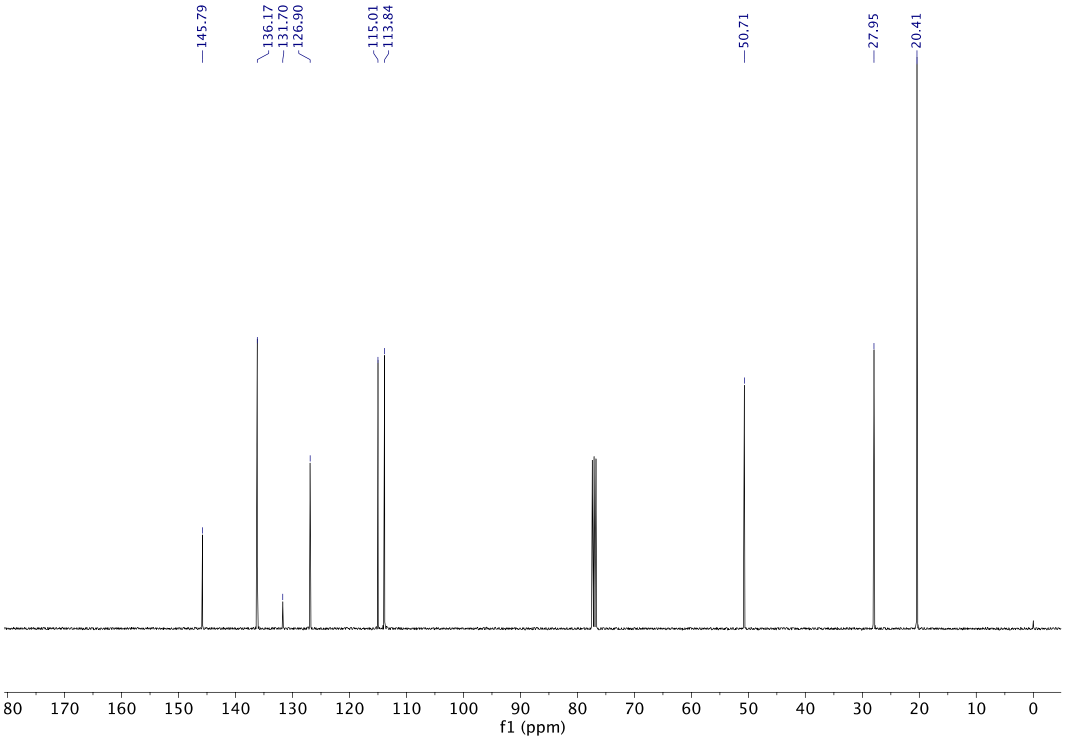

^{1}H and ^{13}C spectra for 1-Isobutyl-2-methyl-1*H*-benzo[*d*]imidazole (**15**)

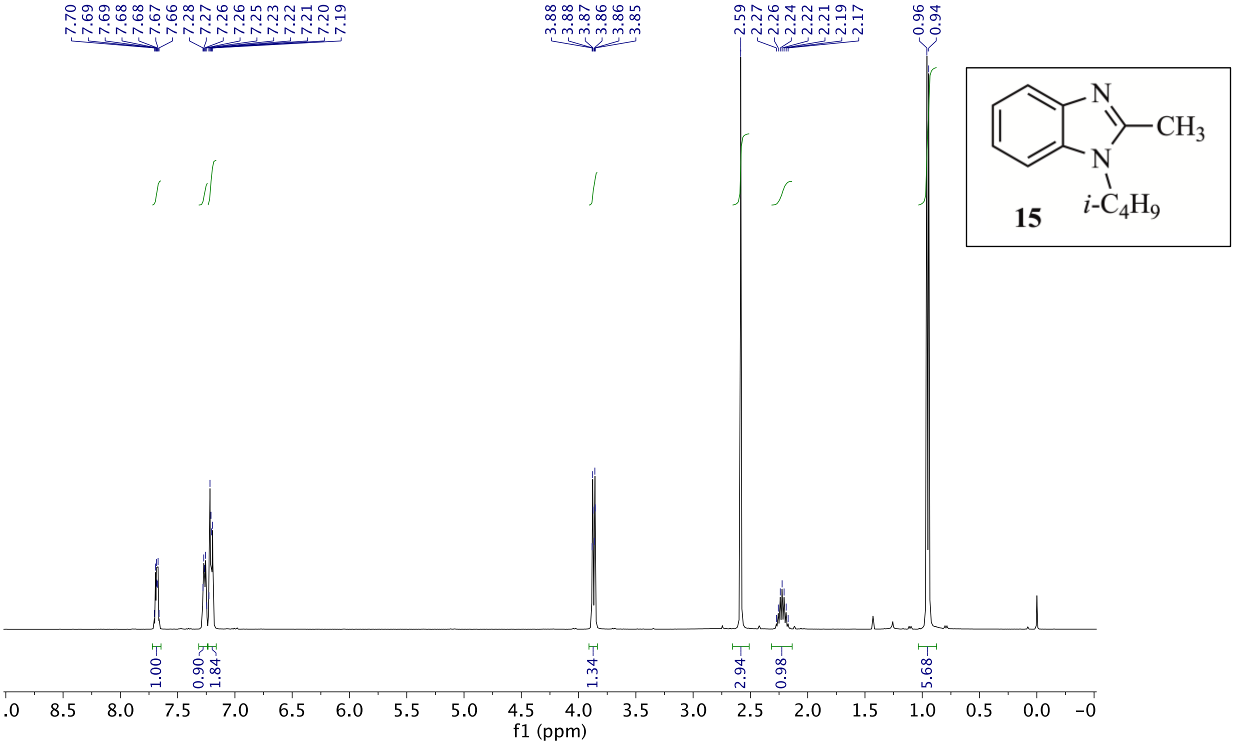

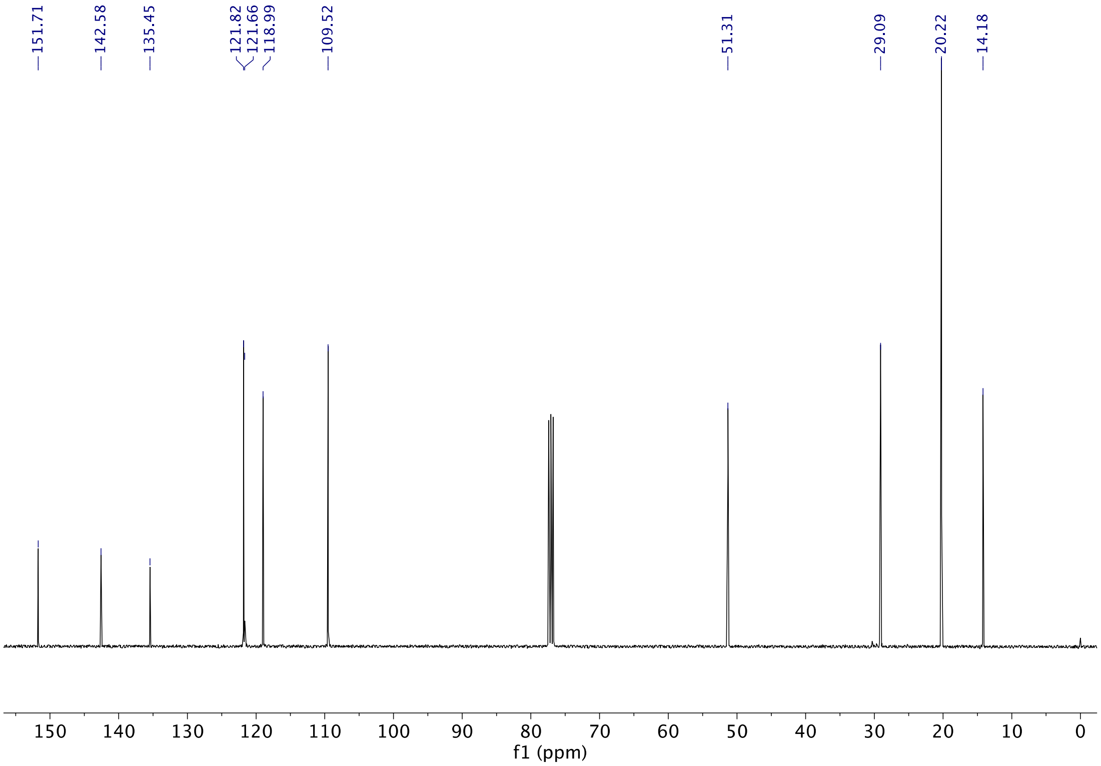

^{1}H and ^{13}C spectra for 1-Isobutyl-2-phenyl-1*H*-benzo[*d*]imidazole (**16**)

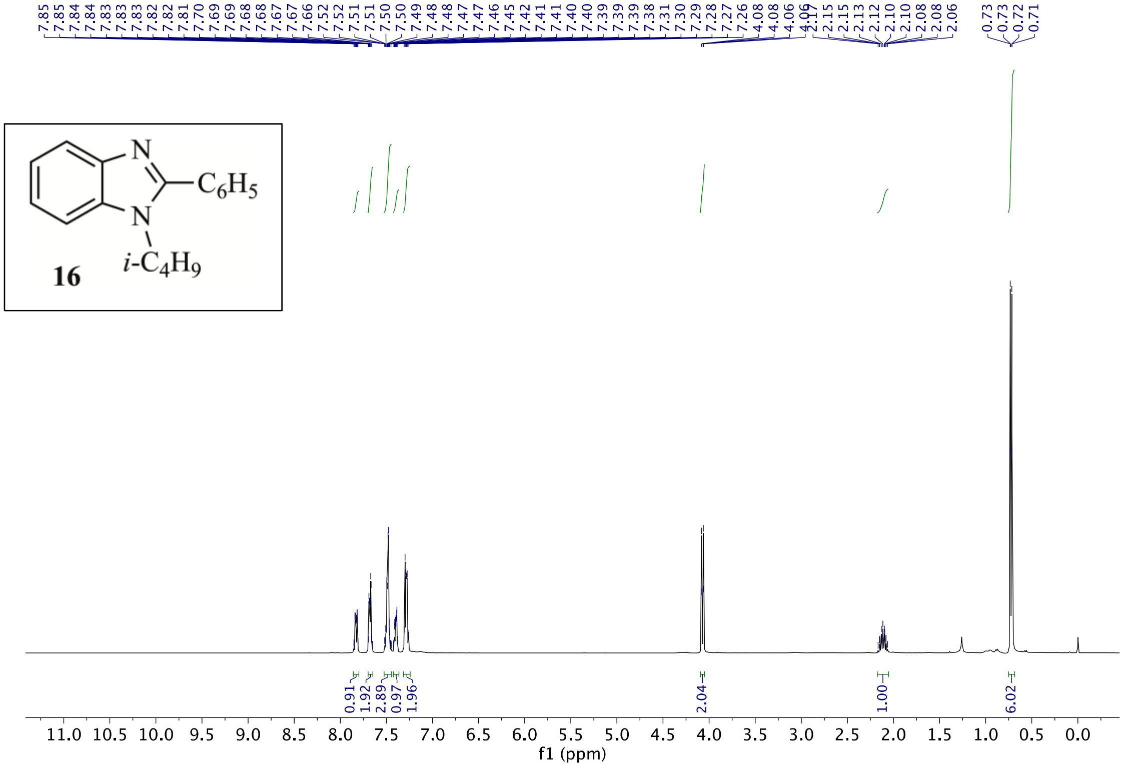

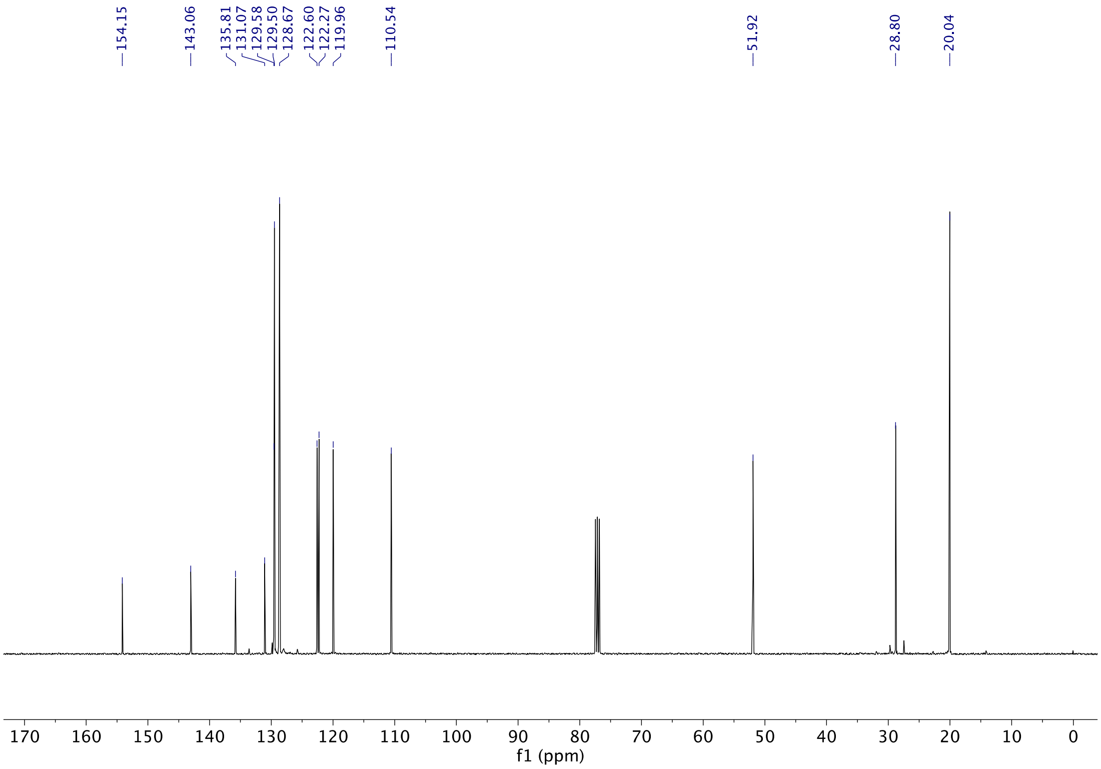

^{1}H and ^{13}C spectra for 1-Isobutyl-2-(4-methoxyphenyl)-1*H*-benzo[*d*]imidazole (**17**)

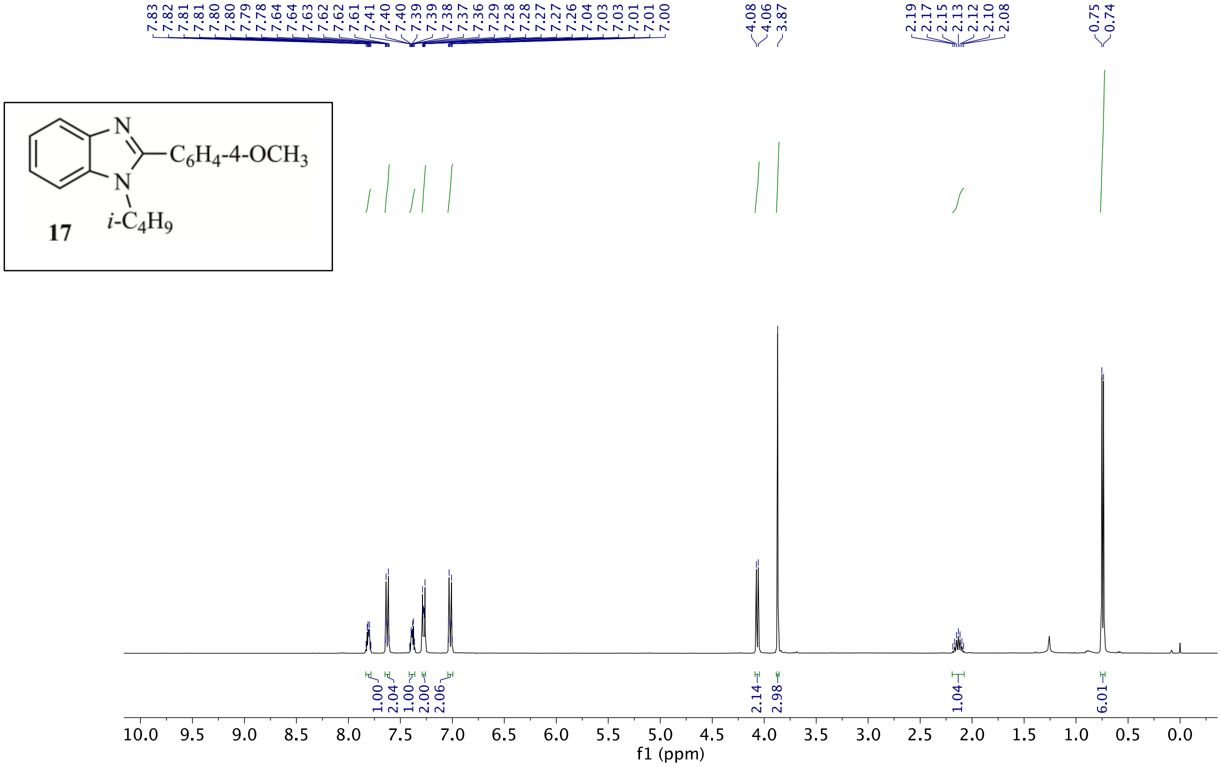

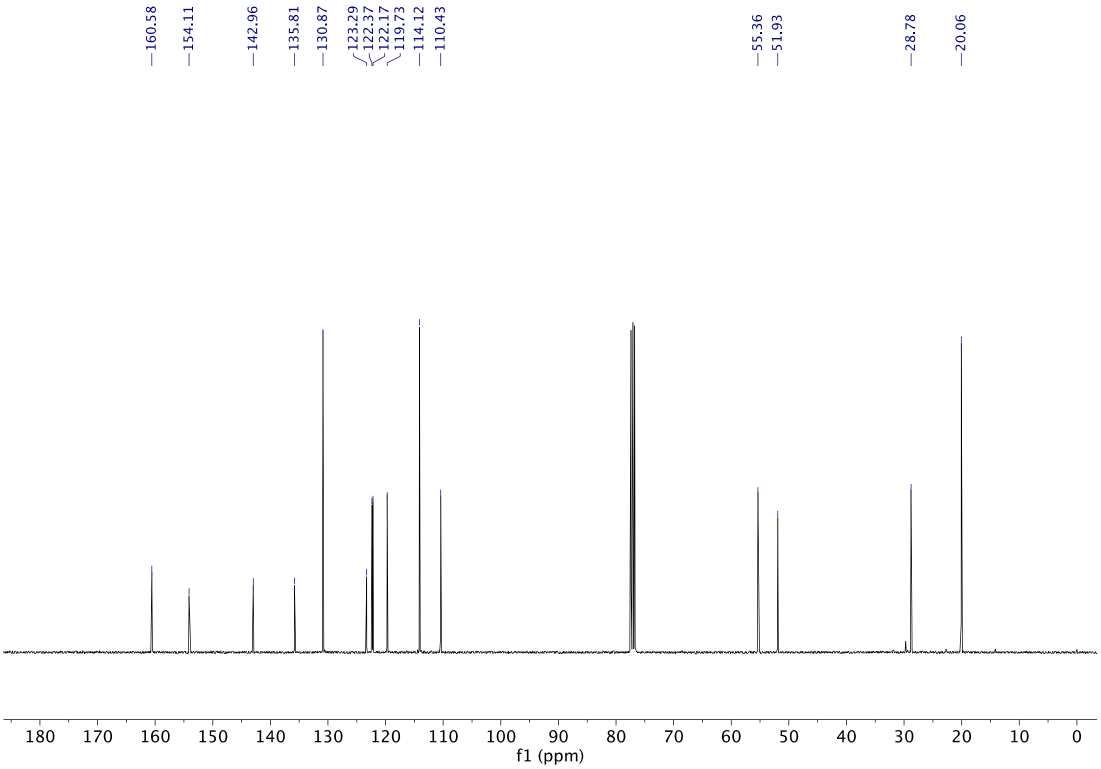

^{1}H and ^{13}C spectra for 1-Isobutyl-2-(4-(trifluoromethyl)phenyl)-1*H*-benzo[*d*]imidazole (**18**)

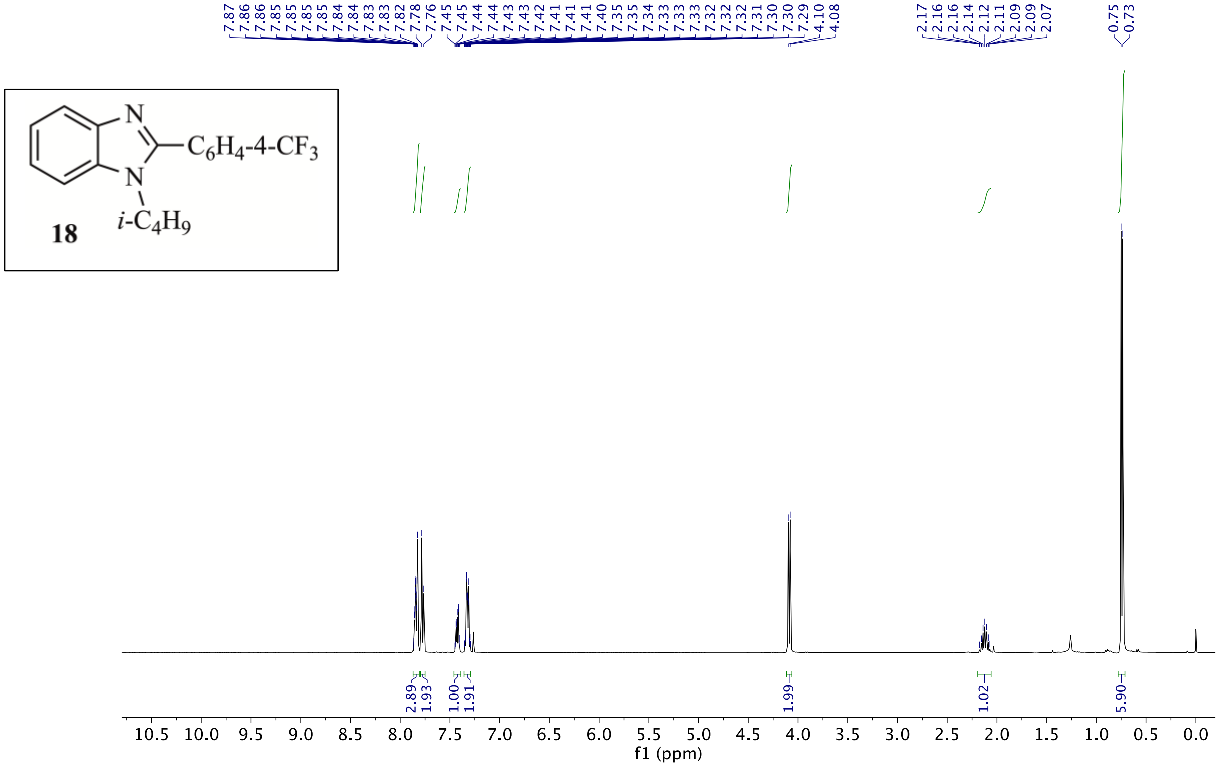

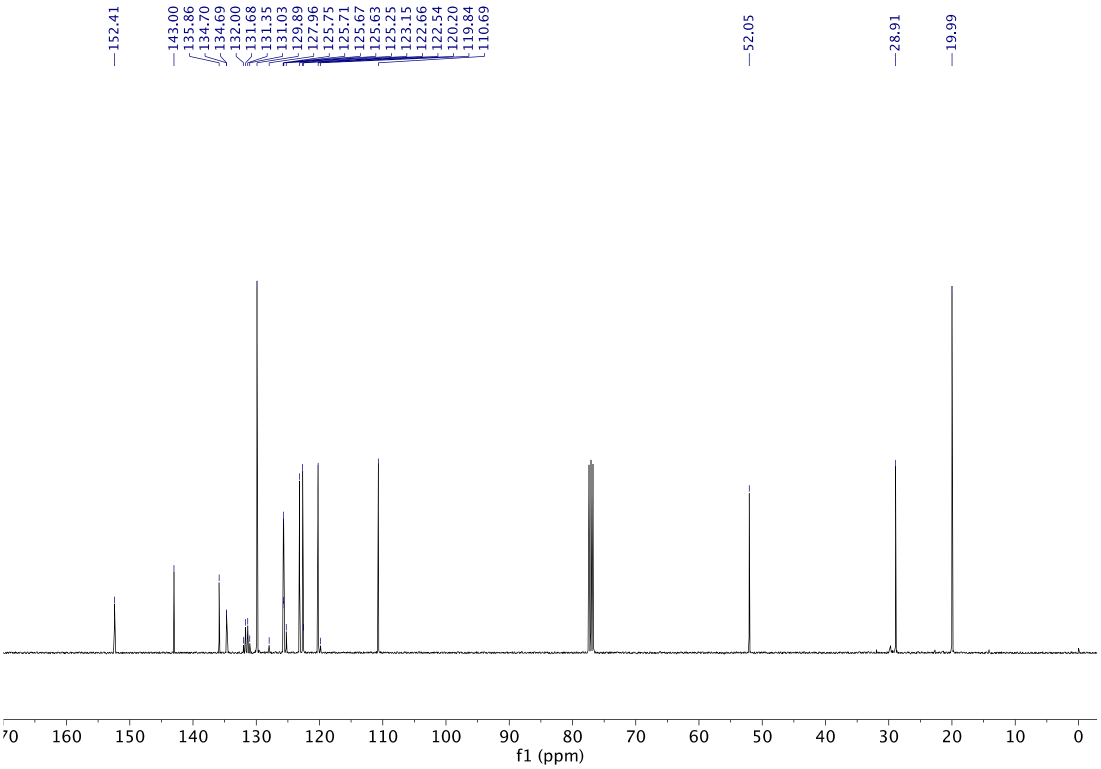

^{19}F spectrum for 1-Isobutyl-2-(4-(trifluoromethyl)phenyl)-1*H*-benzo[*d*]imidazole (**18**)

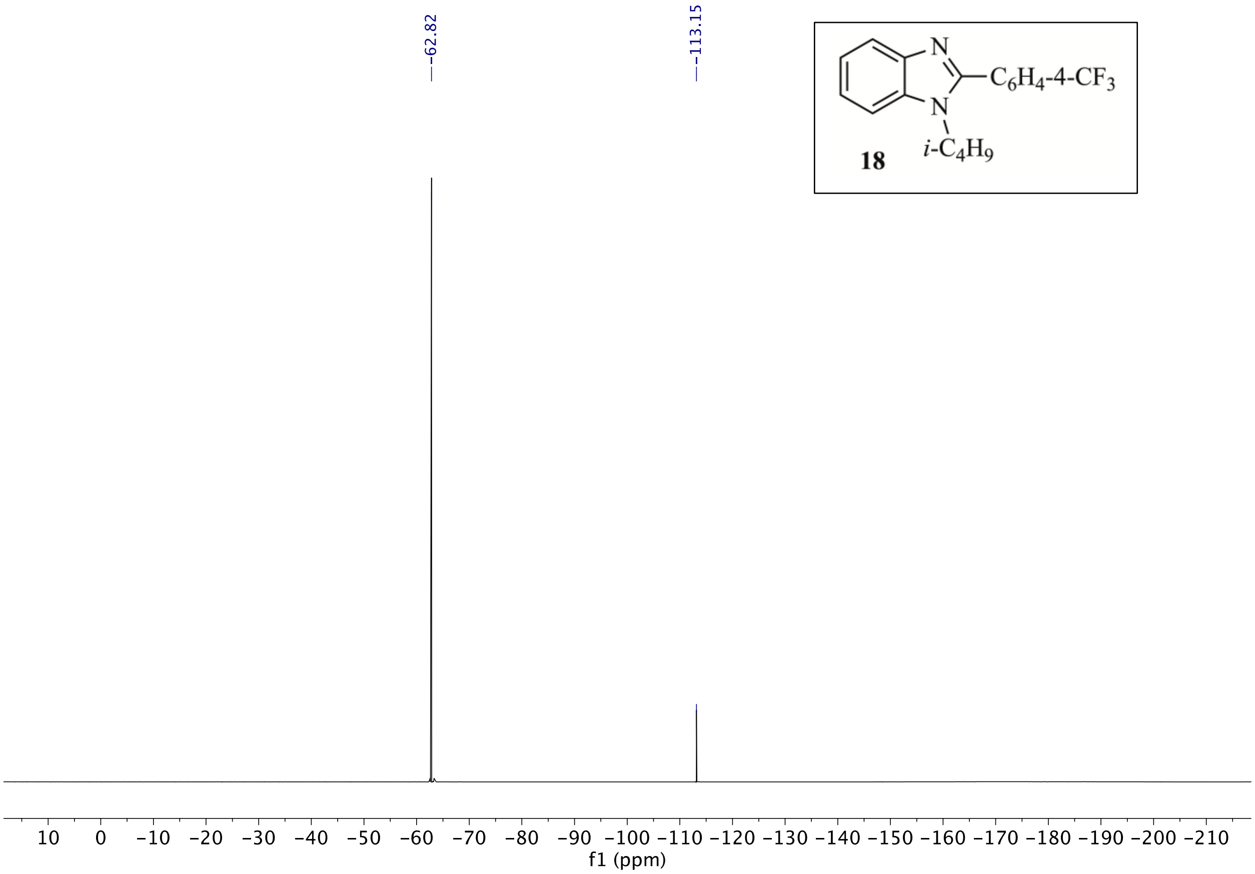

^{1}H and ^{13}C spectra for *N*-Hexyl-2-nitroaniline (**7c**)

7c

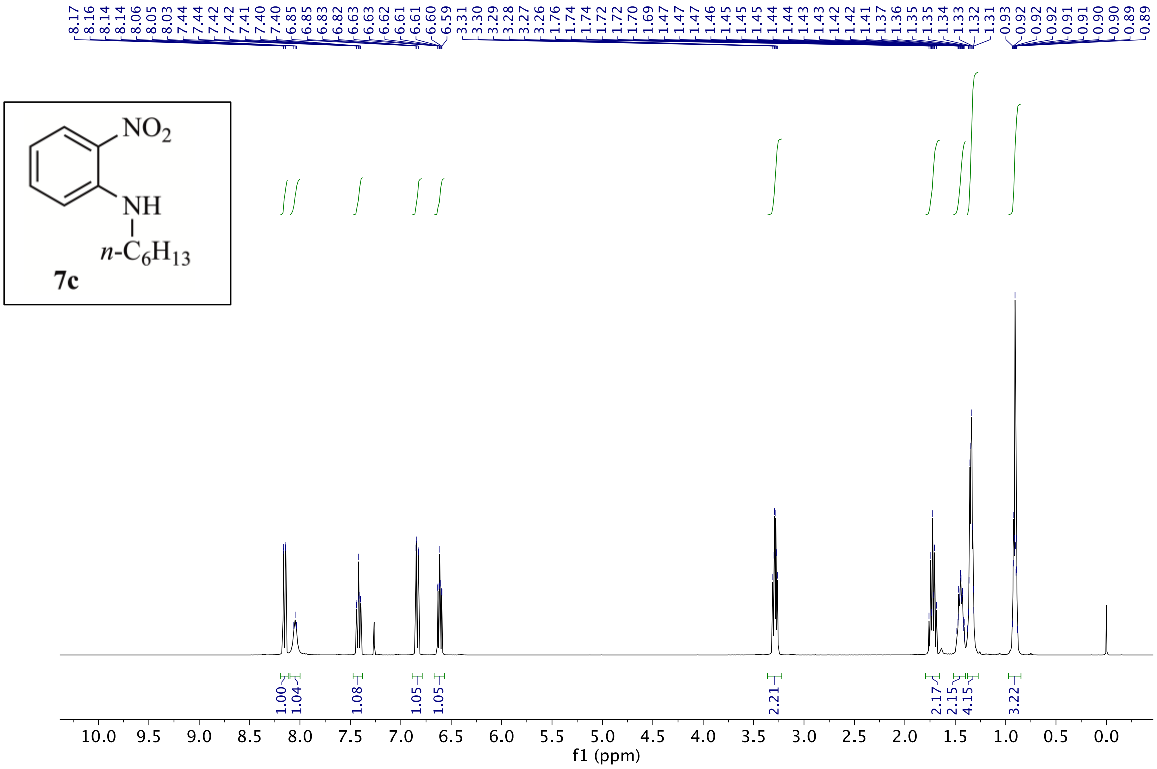

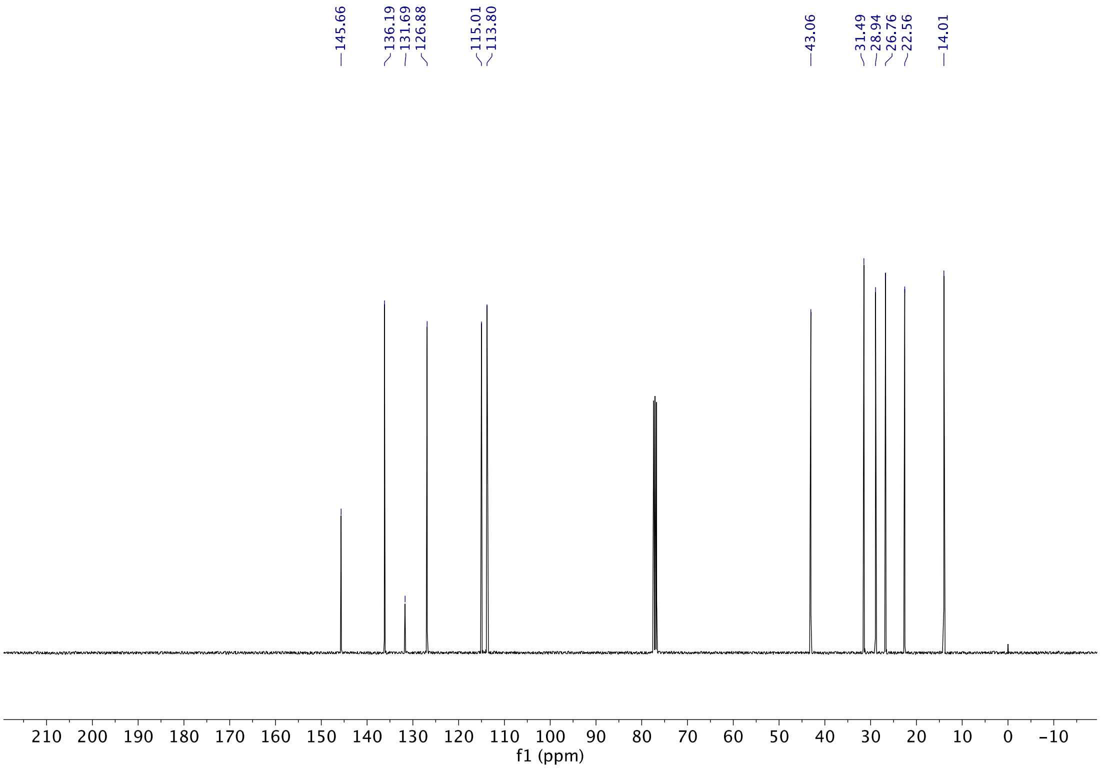

^{1}H and ^{13}C spectra for 1-Hexyl-2-isopropyl-1*H*-benzo[*d*]imidazole (**19**)

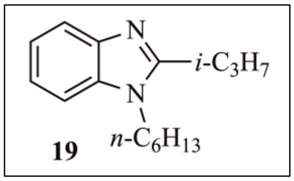

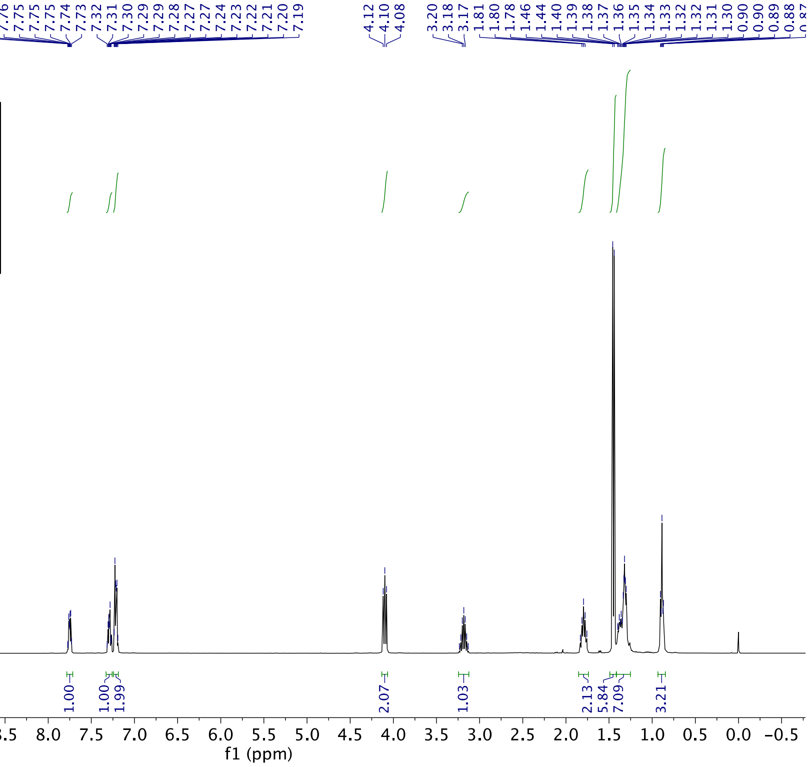

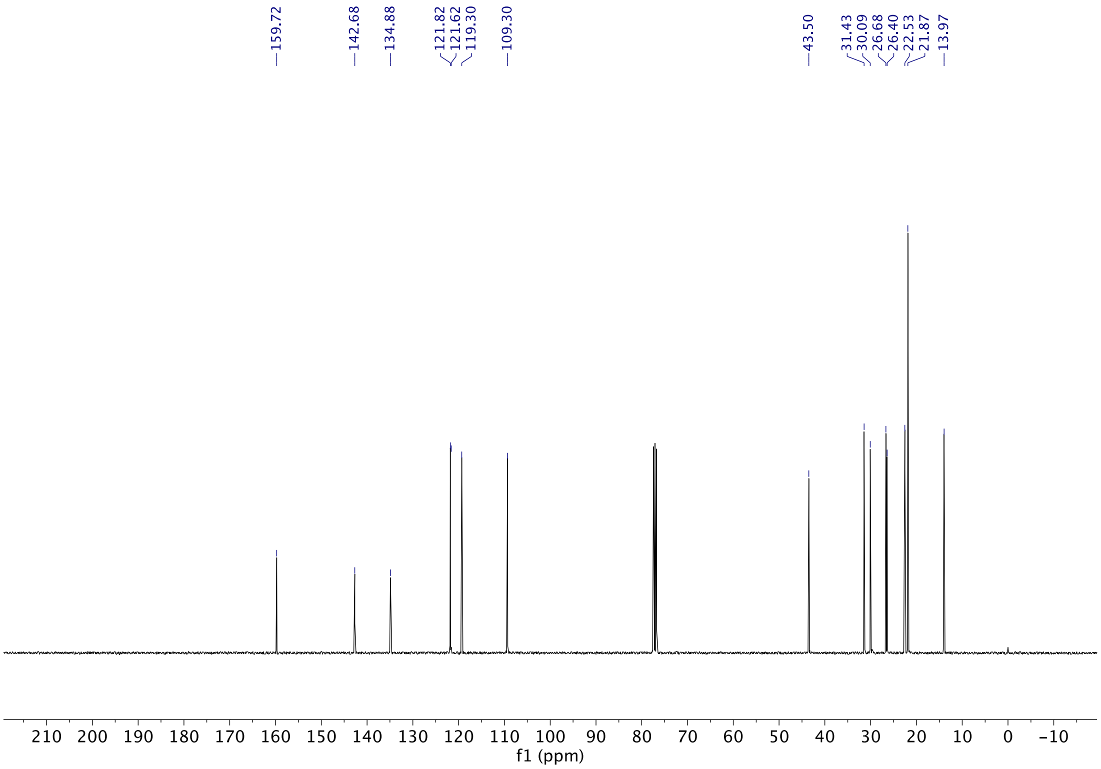

^{1}H and ^{13}C spectra for 1-Hexyl-2-phenyl-1*H*-benzo[*d*]imidazole (**20**)

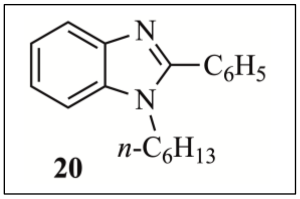

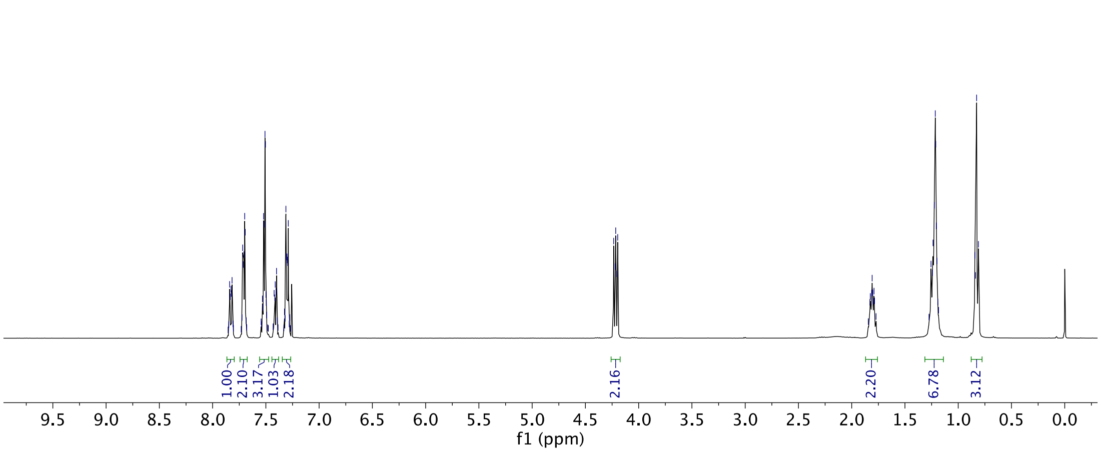

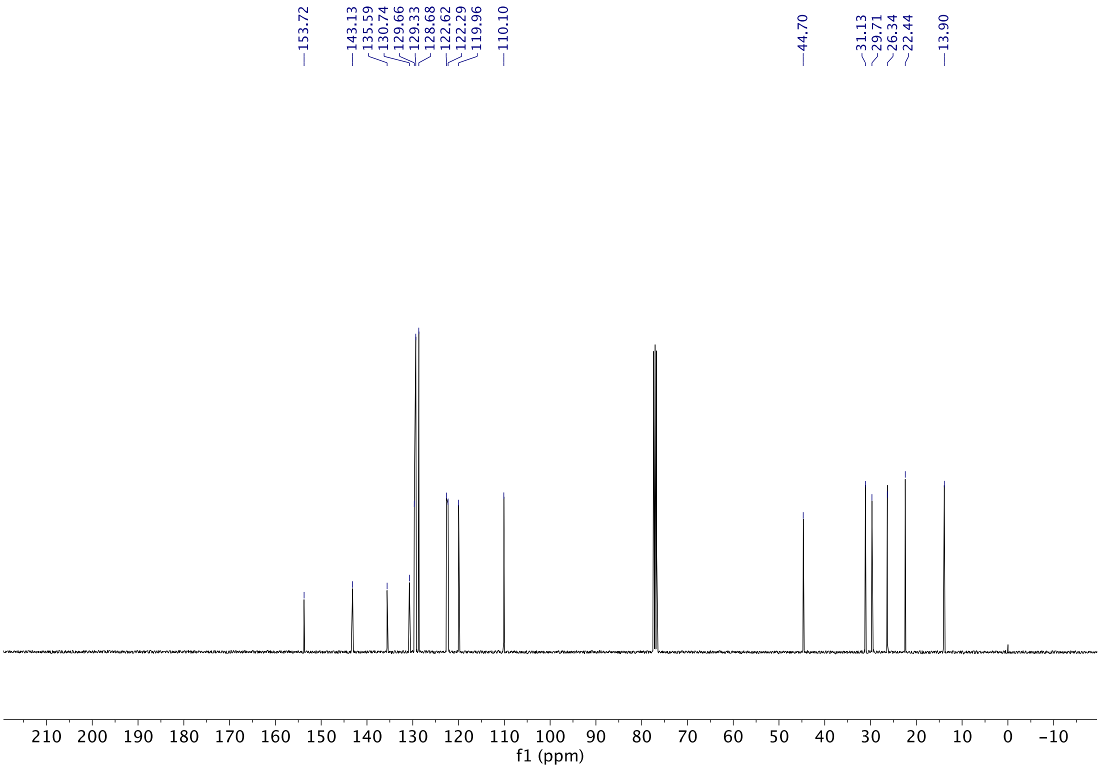

^{1}H and ^{13}C spectra for 1-Hexyl-2-phenethyl-1*H*-benzo[*d*]imidazole (**21**)

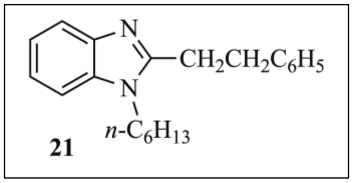

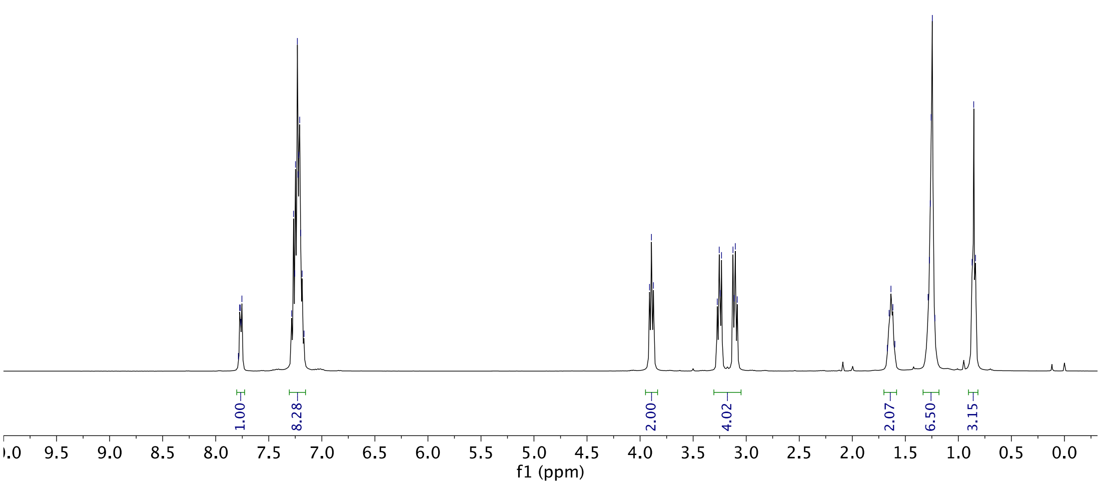

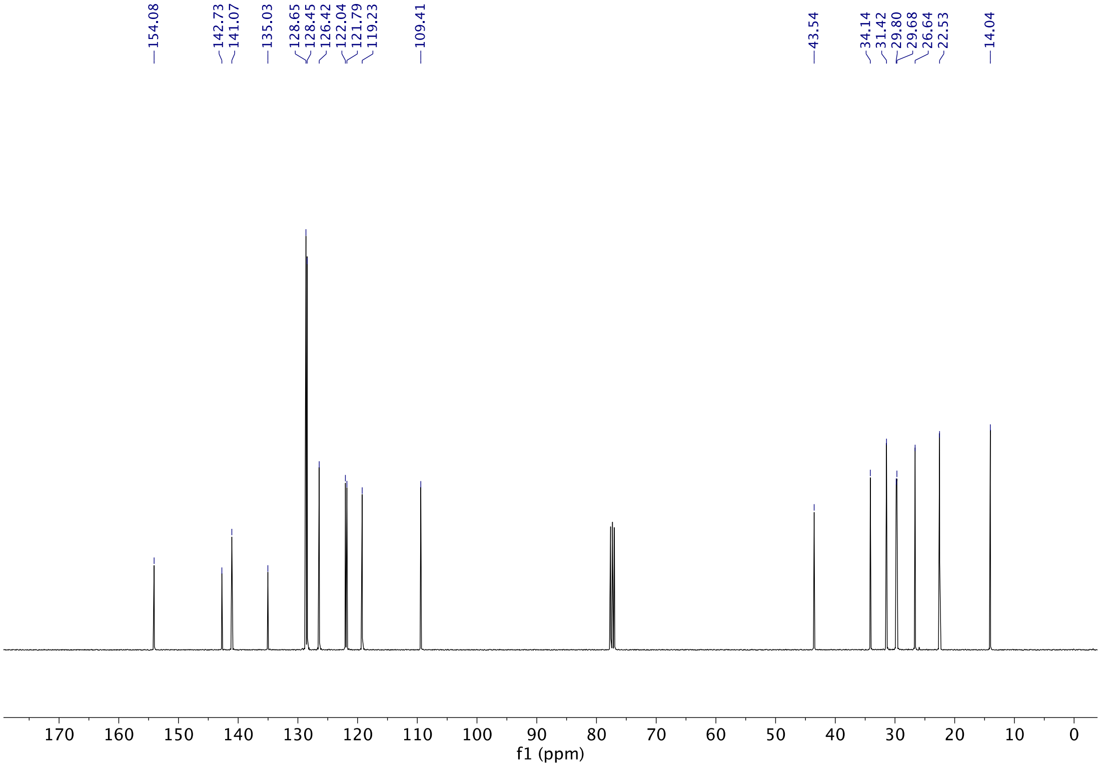

^{1}H and ^{13}C spectra for 2-(3-Chlorophenyl)-1-hexyl-1*H*-benzo[*d*]imidazole (**22**)

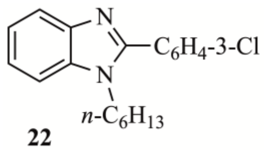

22

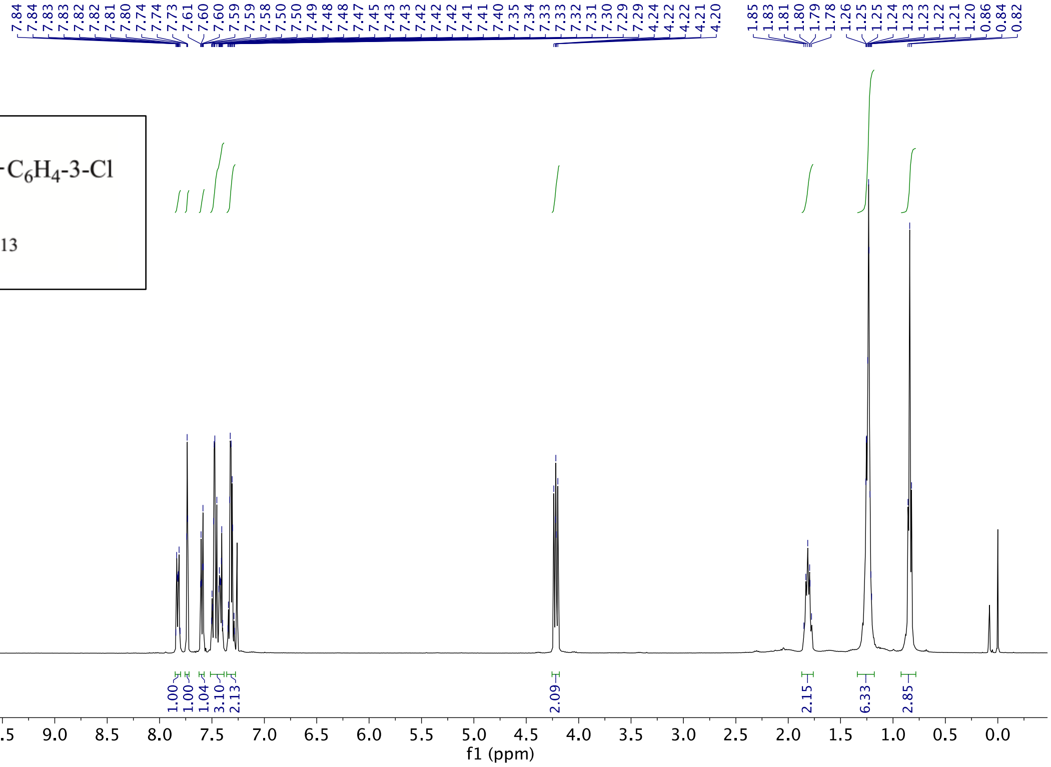

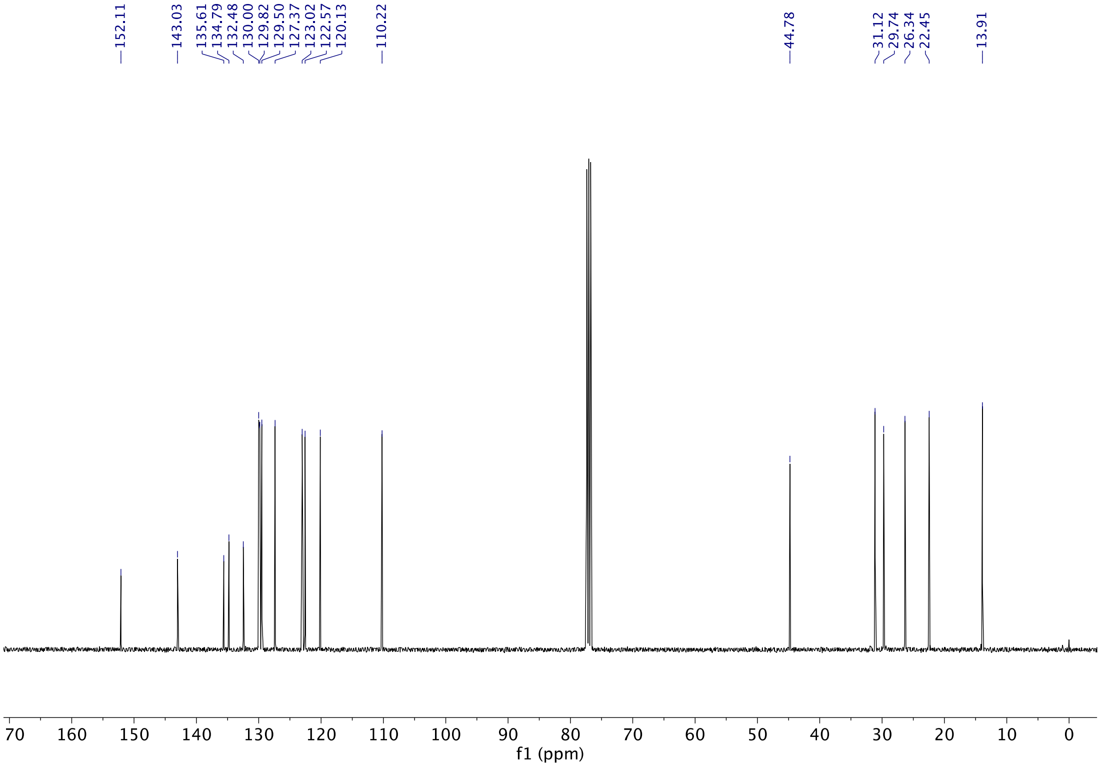

^{1}H and ^{13}C spectra for *N*-(3-Isopropoxypropyl)-2-nitroaniline (**7d**)

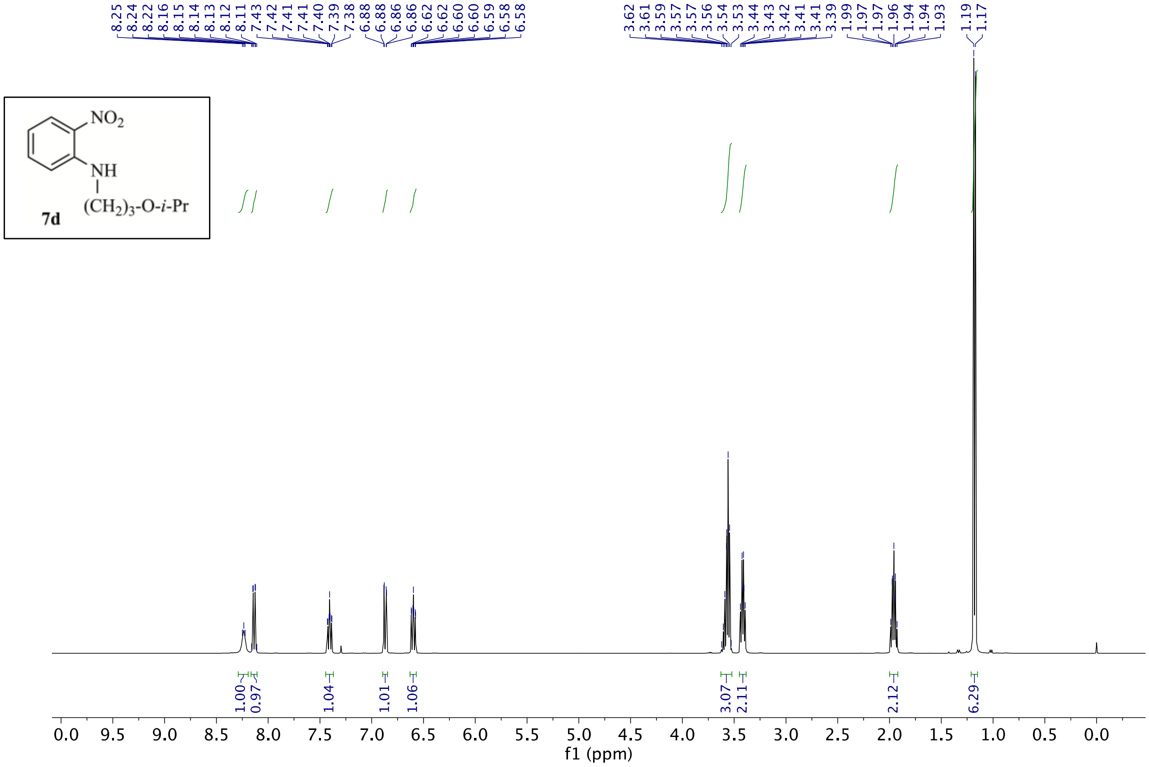

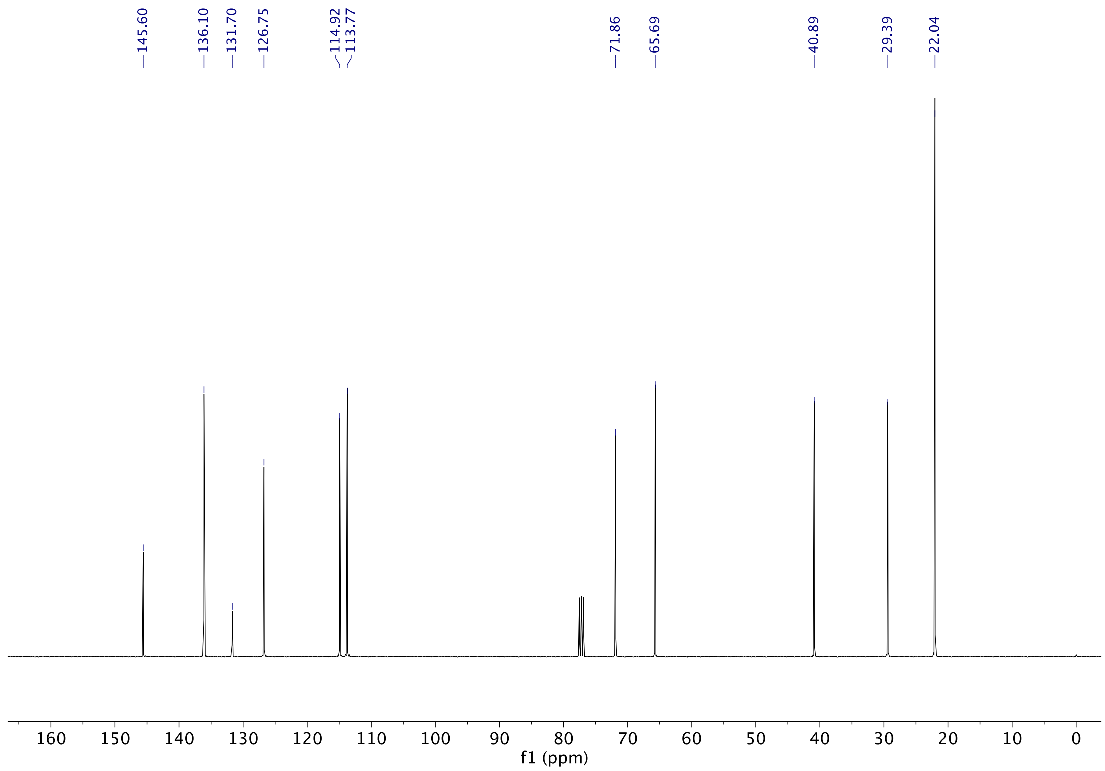

^{1}H and ^{13}C spectra for 1-(3-Isopropoxypropyl)-2-methyl-1*H*-benzo[*d*]imidazole (**23**)

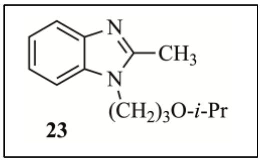

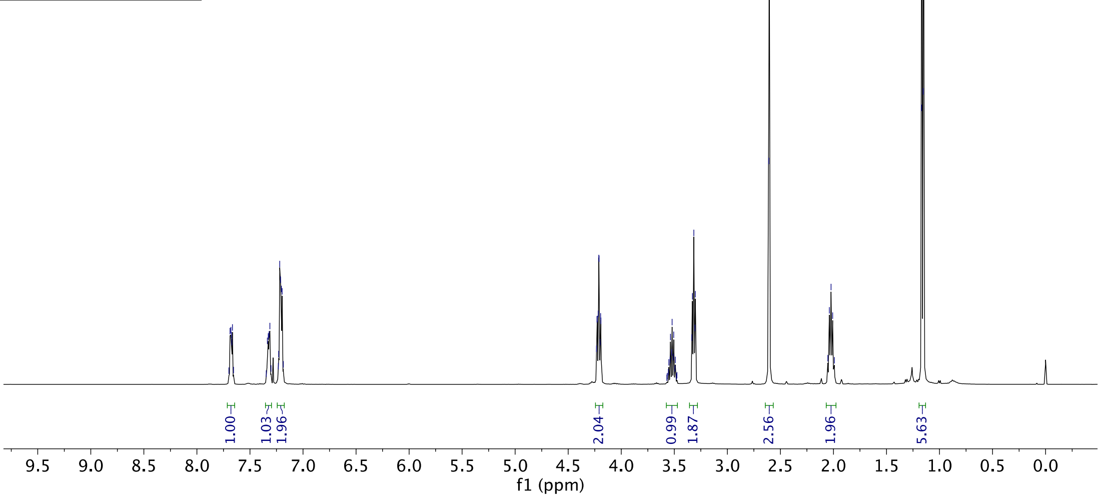

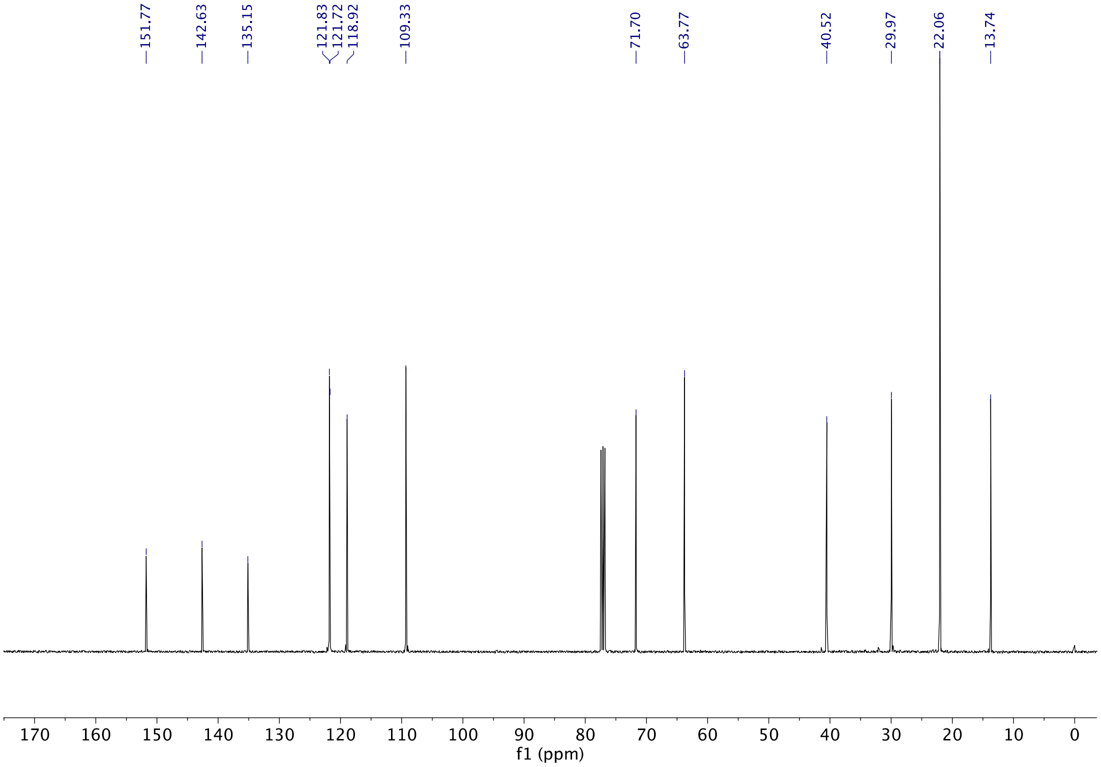

^{1}H and ^{13}C spectra for 1-(3-Isopropoxypropyl)-2-phenyl-1*H*-benzo[*d*]imidazole (**24**)

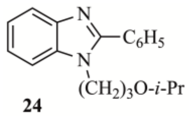

24

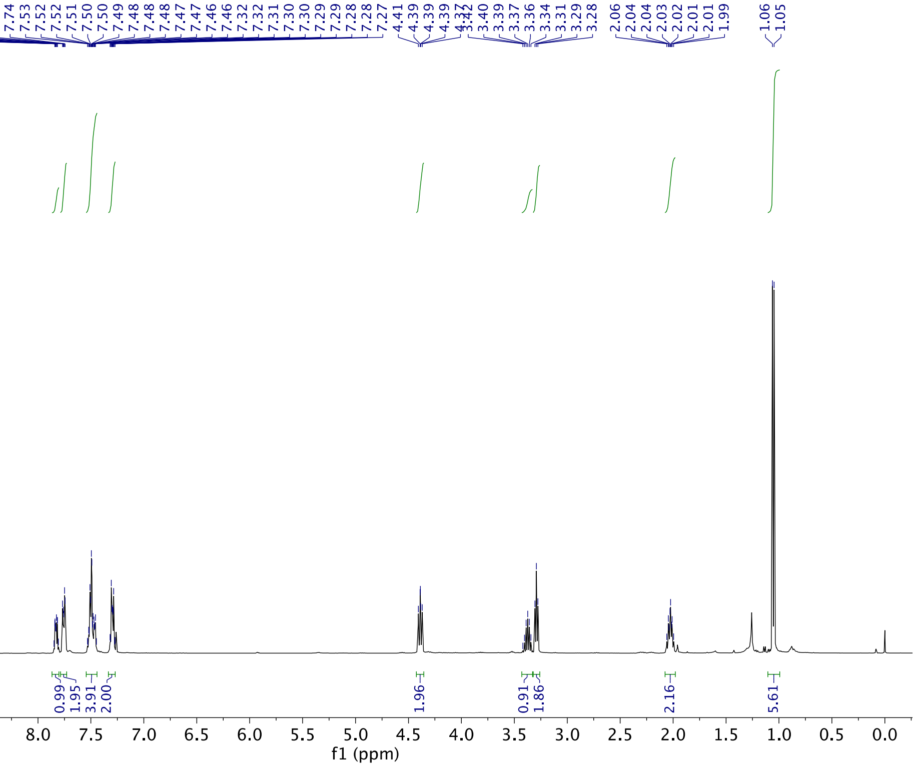

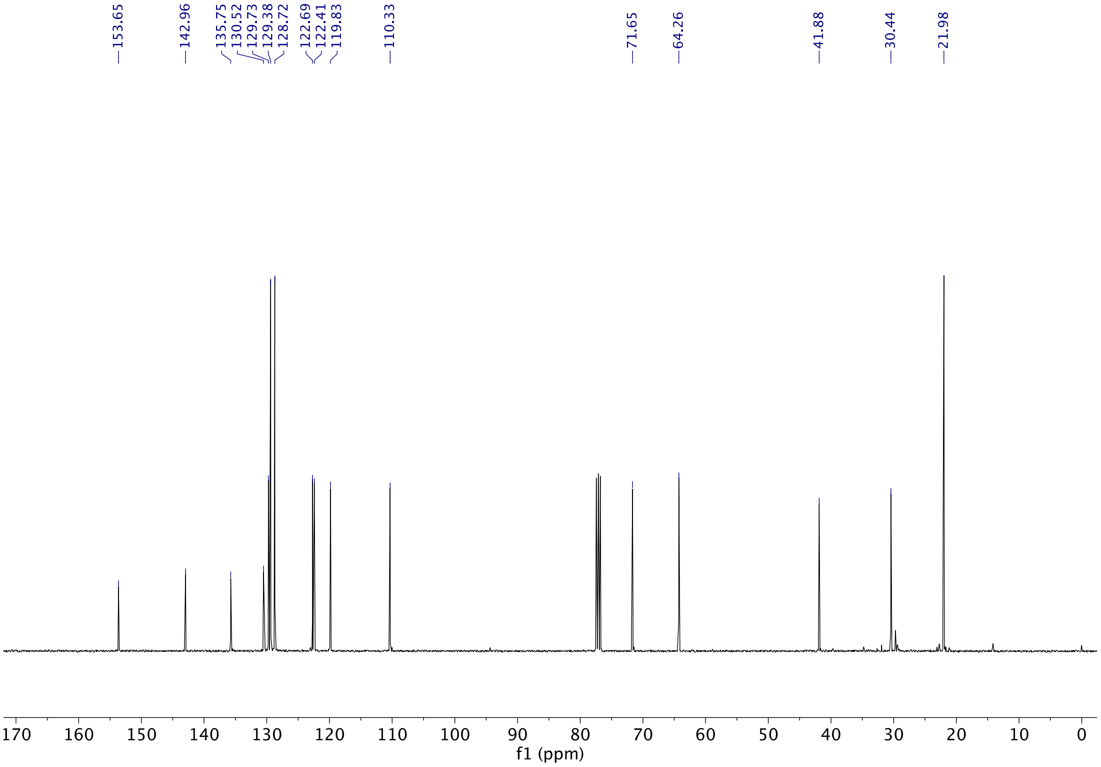

^{1}H and ^{13}C spectra for 1-(2-Fluorophenyl)-1-(3-isopropoxypropyl)-1*H*-benzo[*d*]imidazole (**25**)

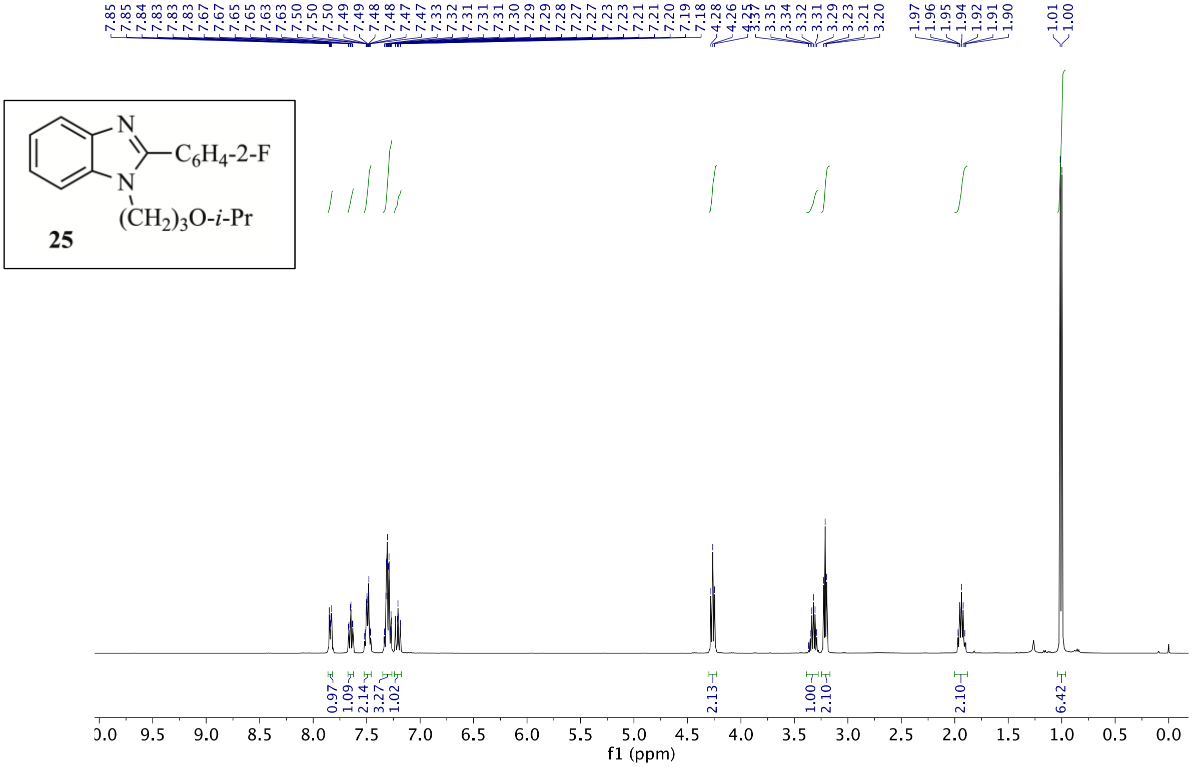

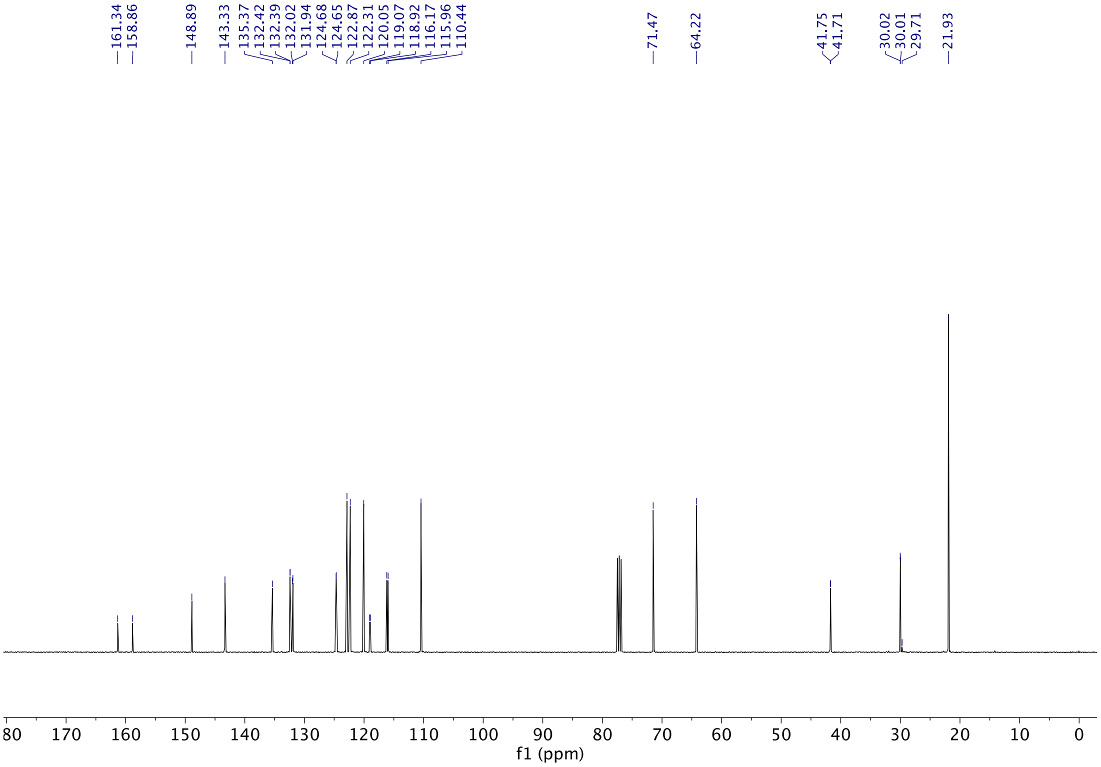

^{19}F spectrum for 1-(2-Fluorophenyl)-1-(3-isopropoxypropyl)-1*H*-benzo[*d*]imidazole (**25**)

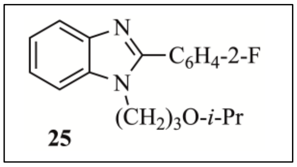

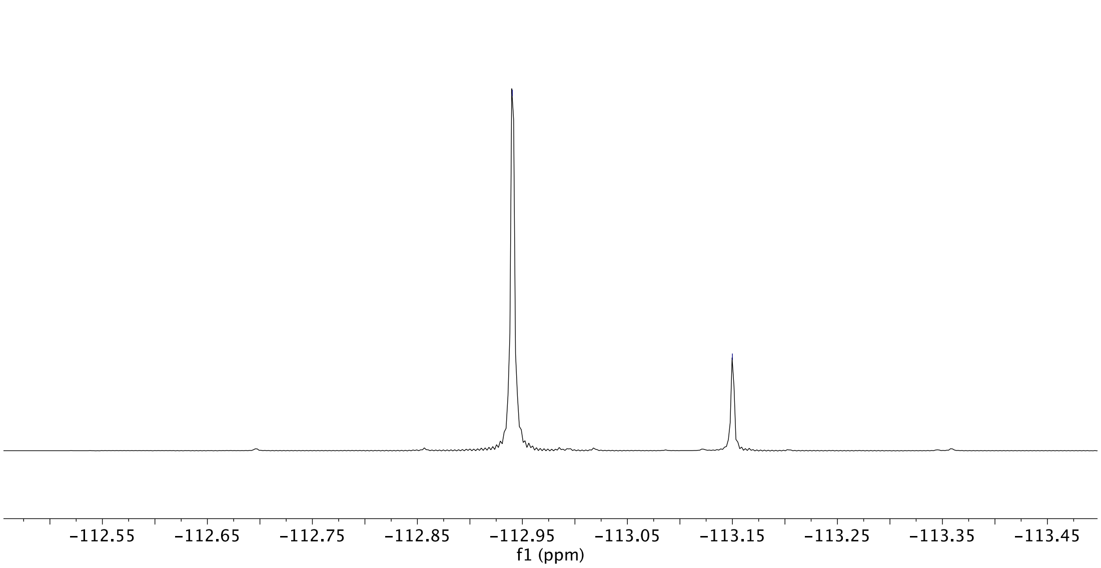

^{1}H and ^{13}C spectra for
2-(4-Fluoro-3-methylphenyl)-1-(3-isopropoxypropyl)-1*H*-benzo[*d*]imidazole (**26**)

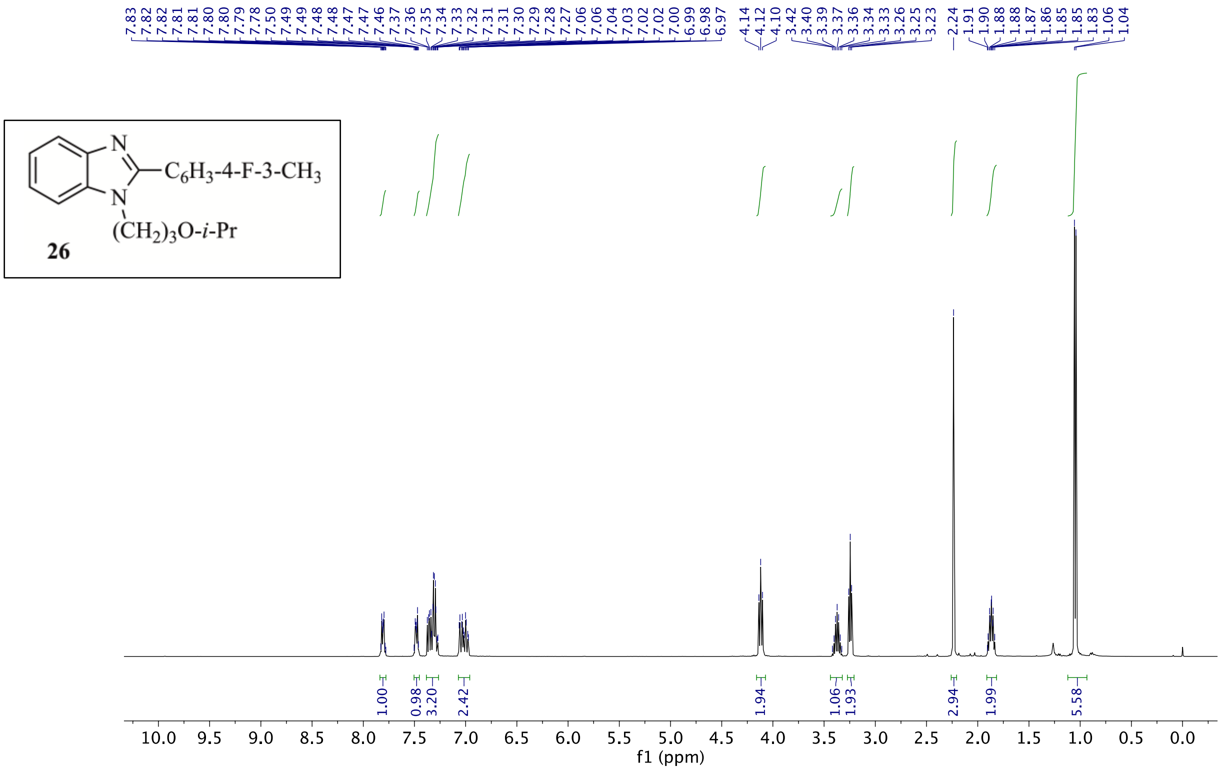

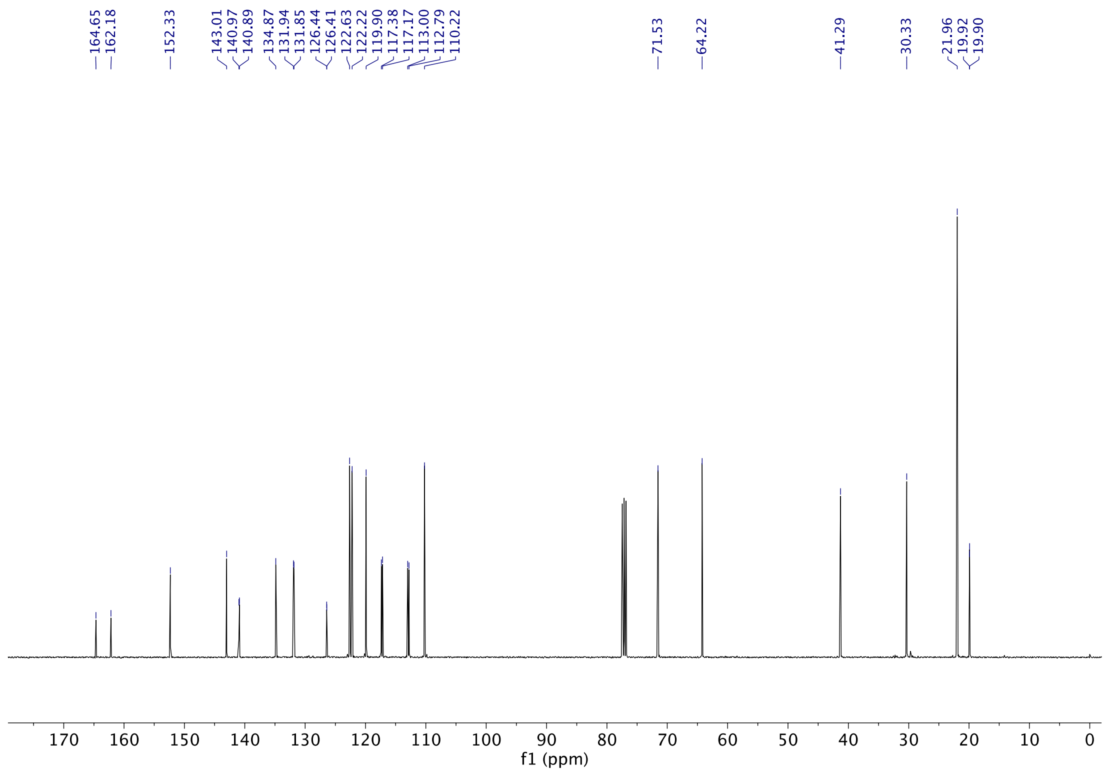

^{19}F spectrum for

2-(4-Fluoro-3-methylphenyl)-1-(3-isopropoxypropyl)-1*H*-benzo[*d*]imidazole (**26**)

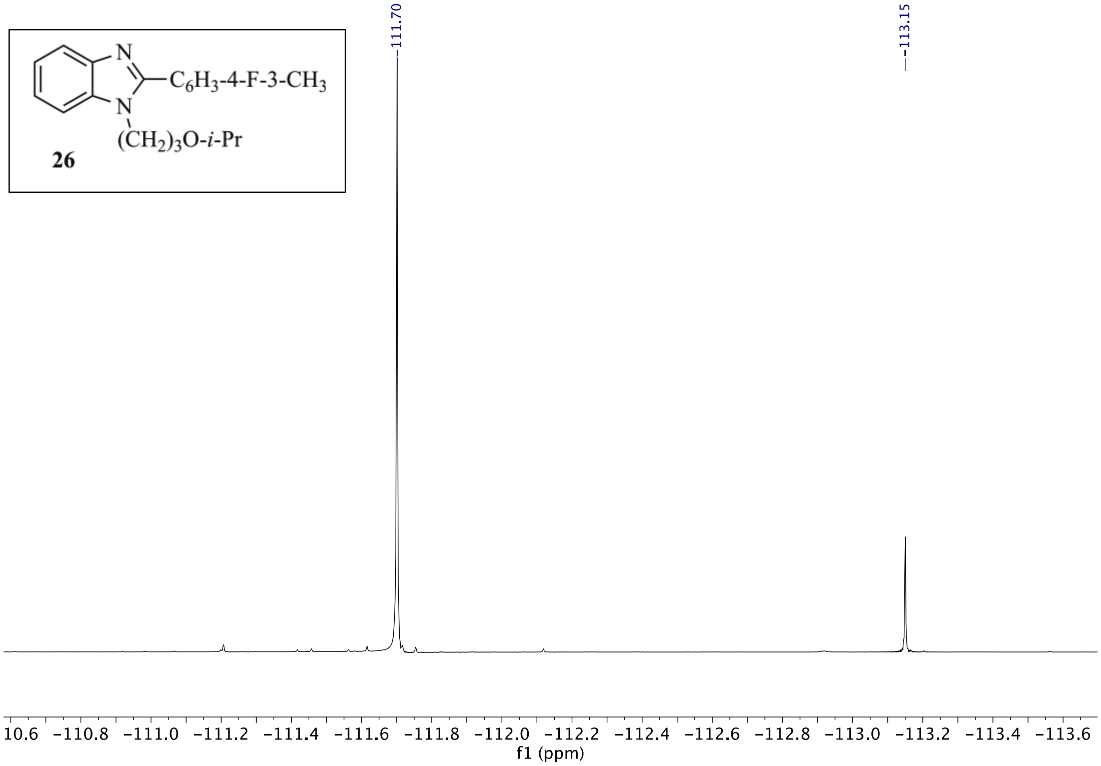

^{1}H and ^{13}C spectra for *N*-Benzyl-2-nitroaniline (**7e**)

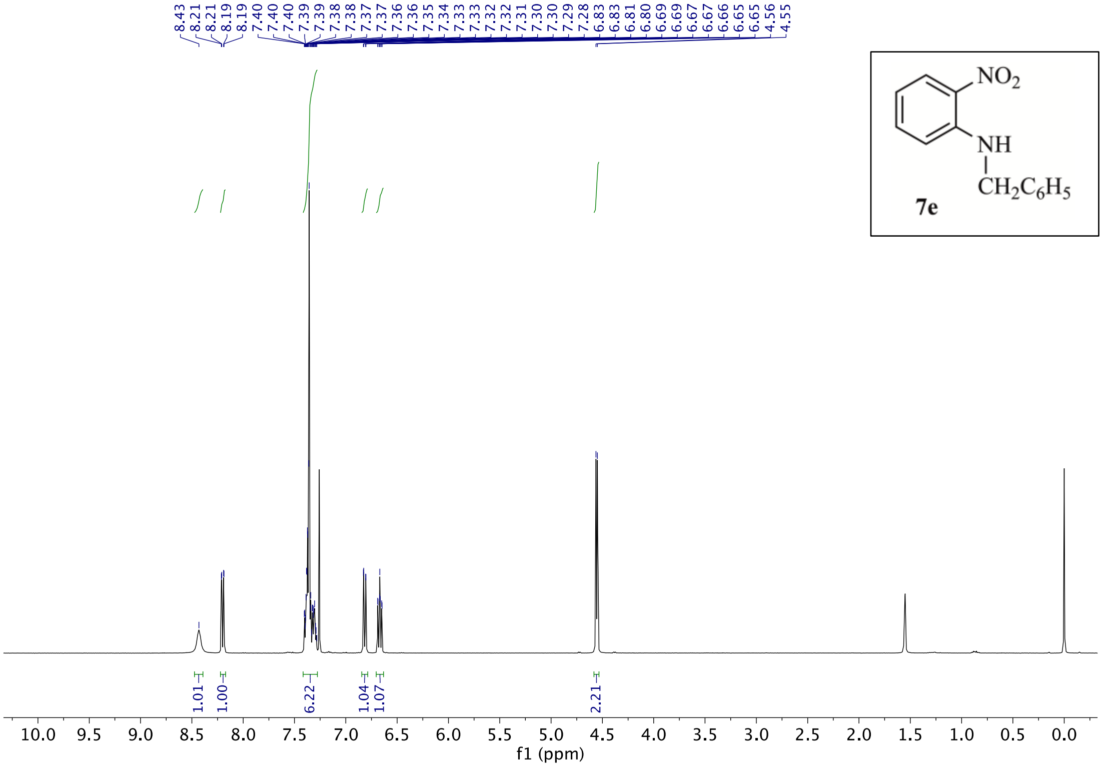

7e

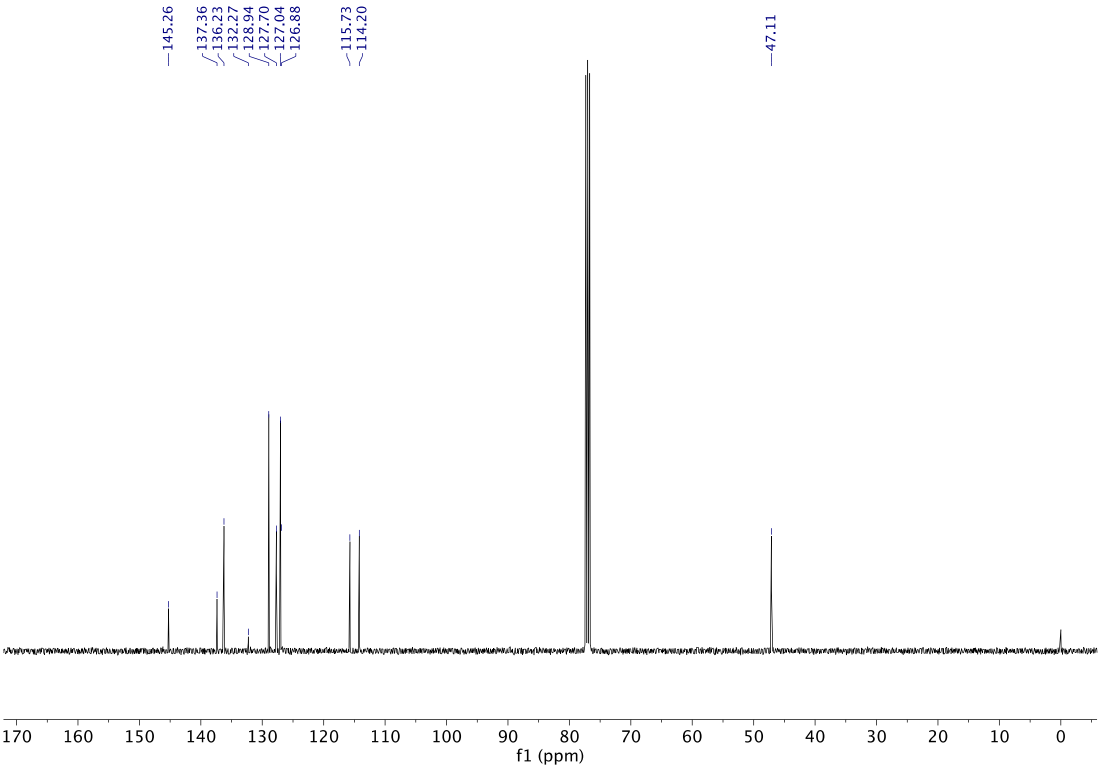

^{1}H and ^{13}C spectra for 1-Benzyl-2-methyl-1*H*-benzo[*d*]imidazole (**27**)

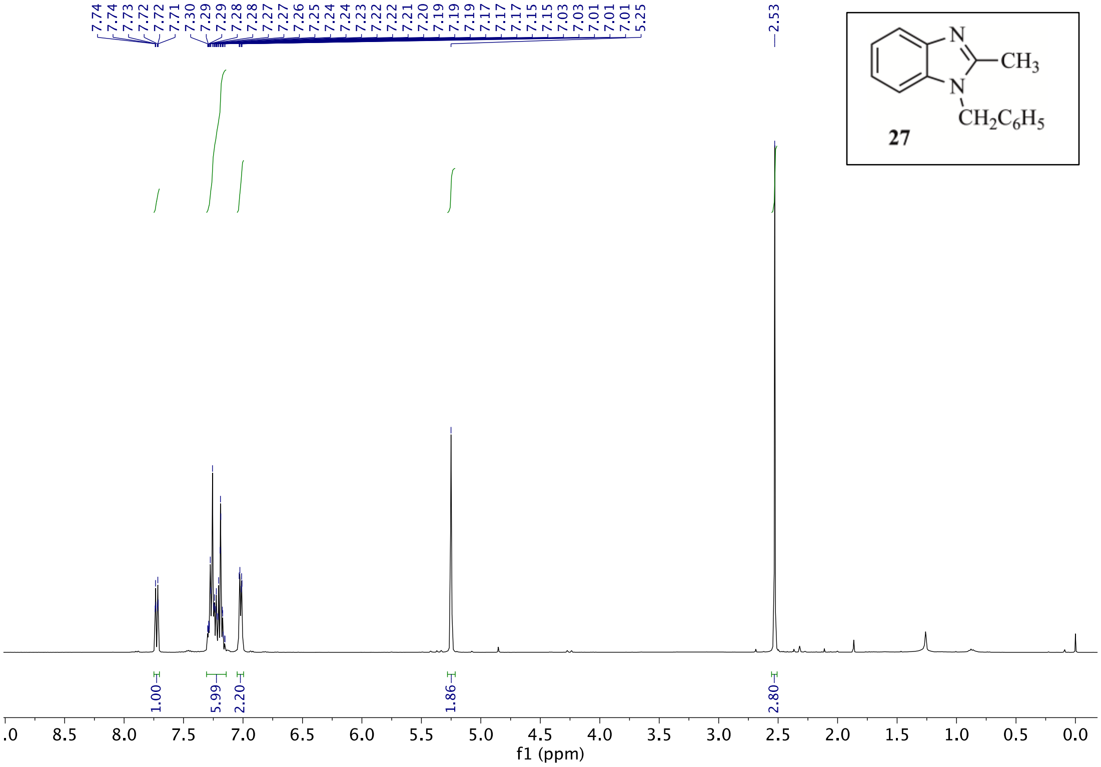

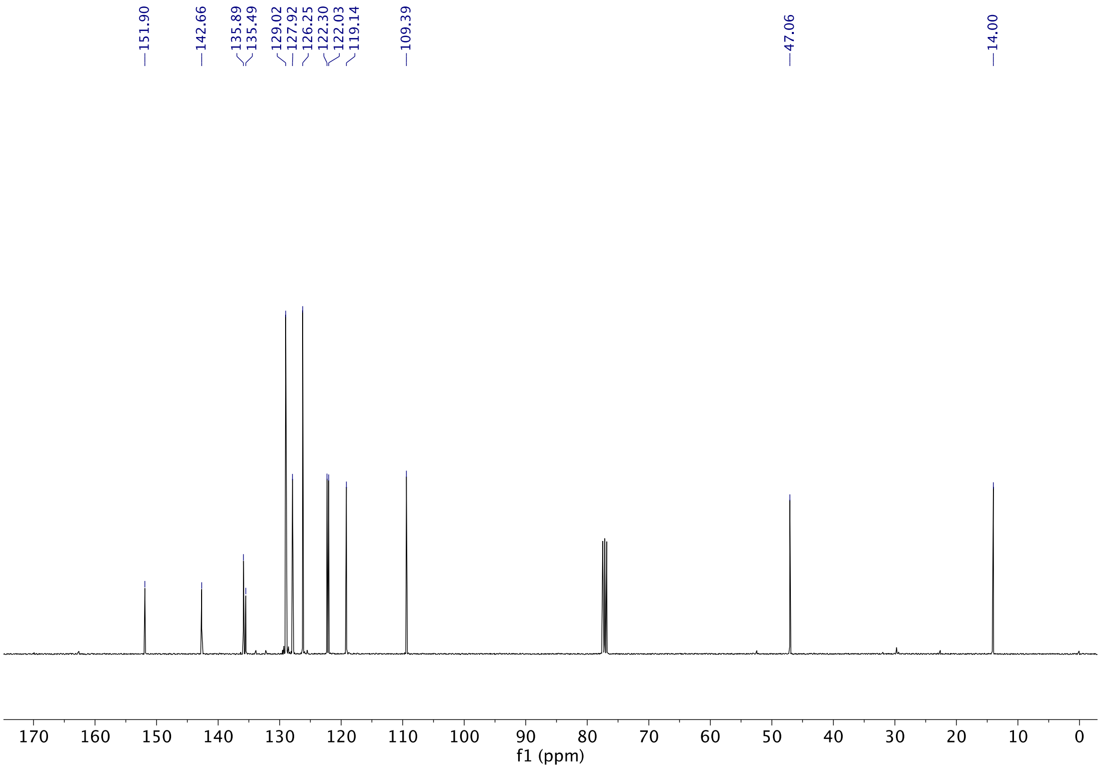

^{1}H and ^{13}C spectra for 1-Benzyl-2-phenyl-1*H*-benzo[*d*]imidazole (**28**)

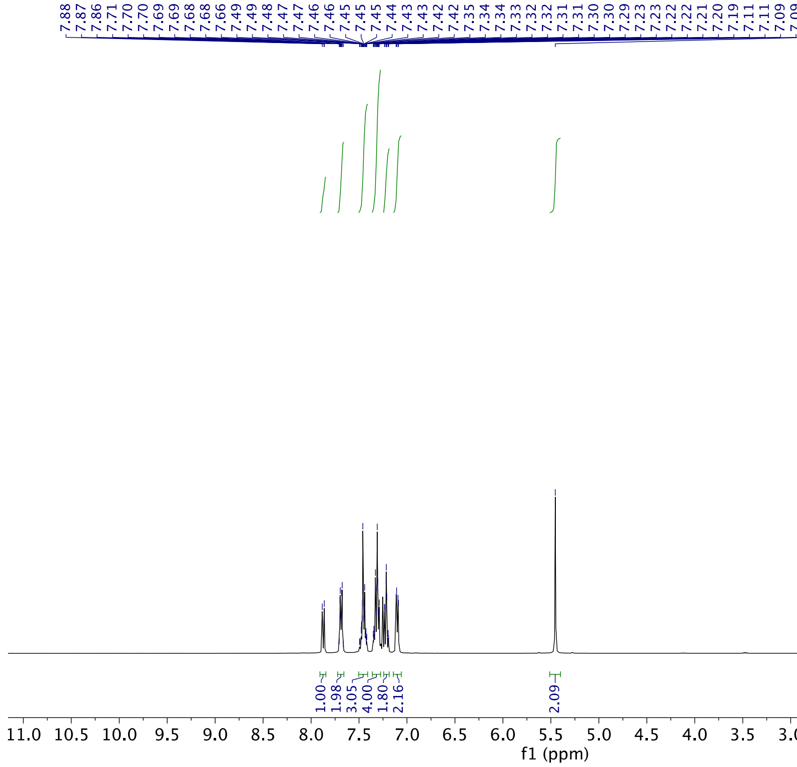

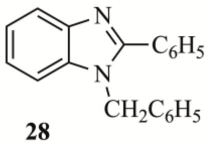

28

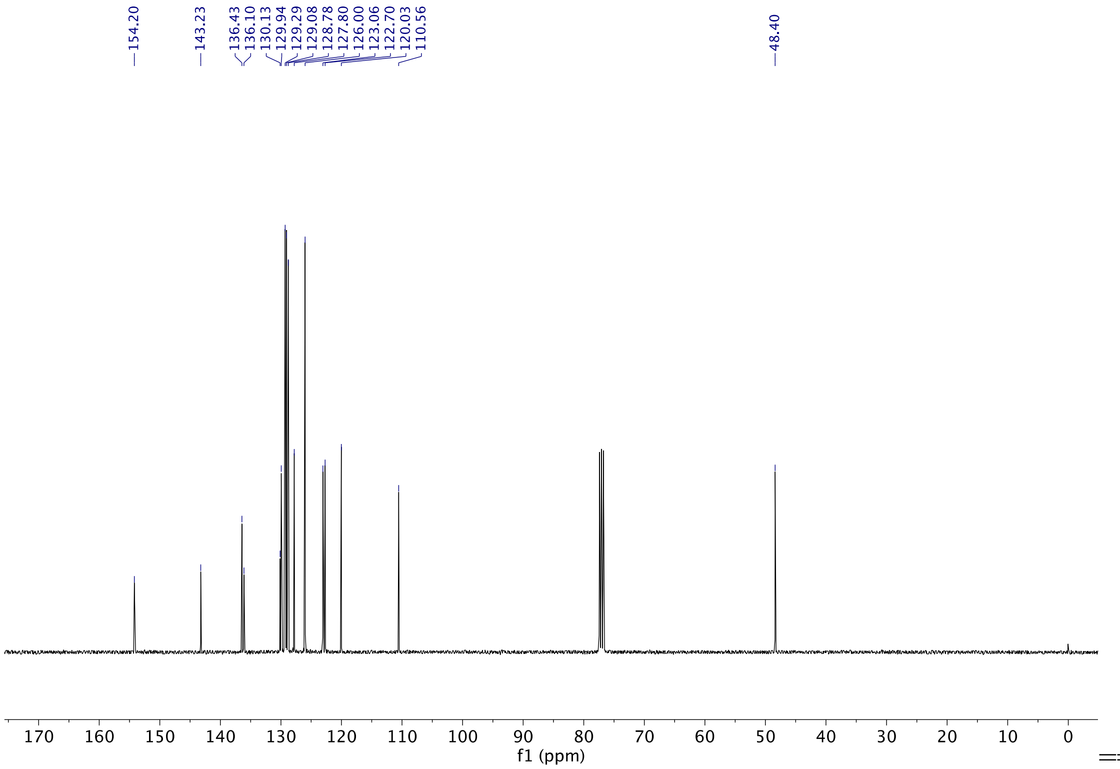

^{1}H and ^{13}C spectra for 1-Benzyl-2-(4-methoxyphenyl)-1*H*-benzo[*d*]imidazole (**29**)

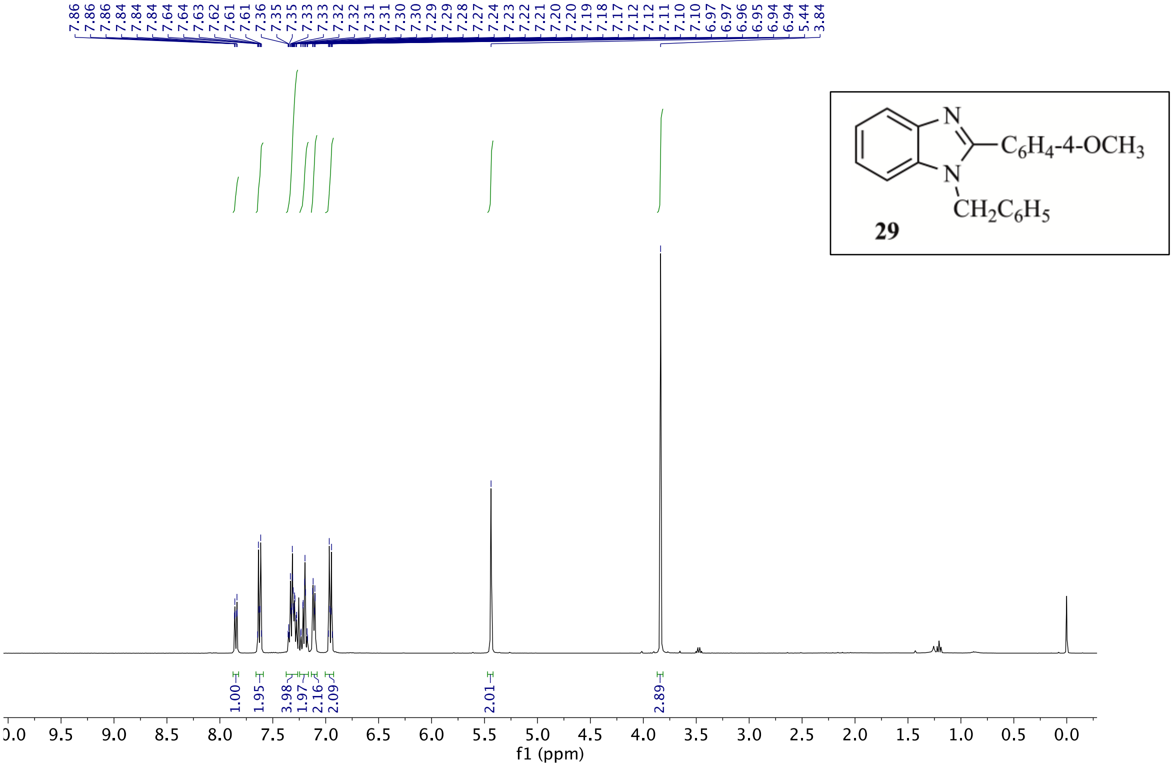

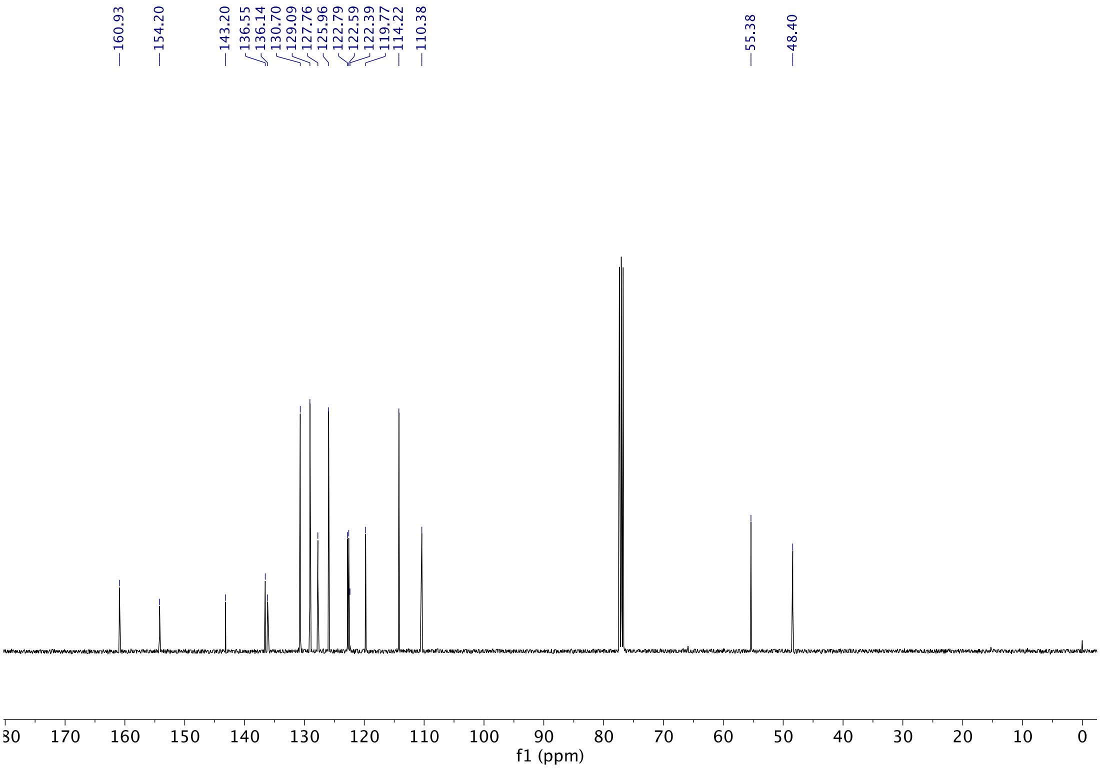

^{1}H and ^{13}C spectra for 1-Benzyl-2-(4-(trifluoromethyl)phenyl)-1*H*-benzo[*d*]imidazole (**30**)

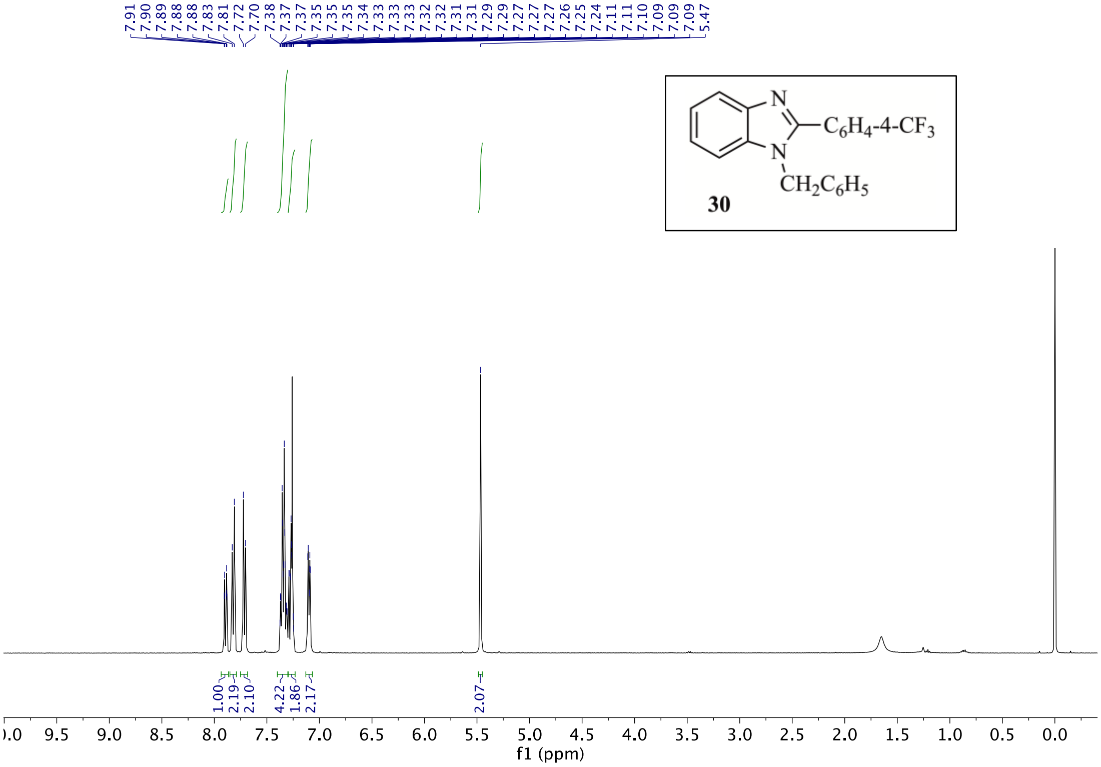

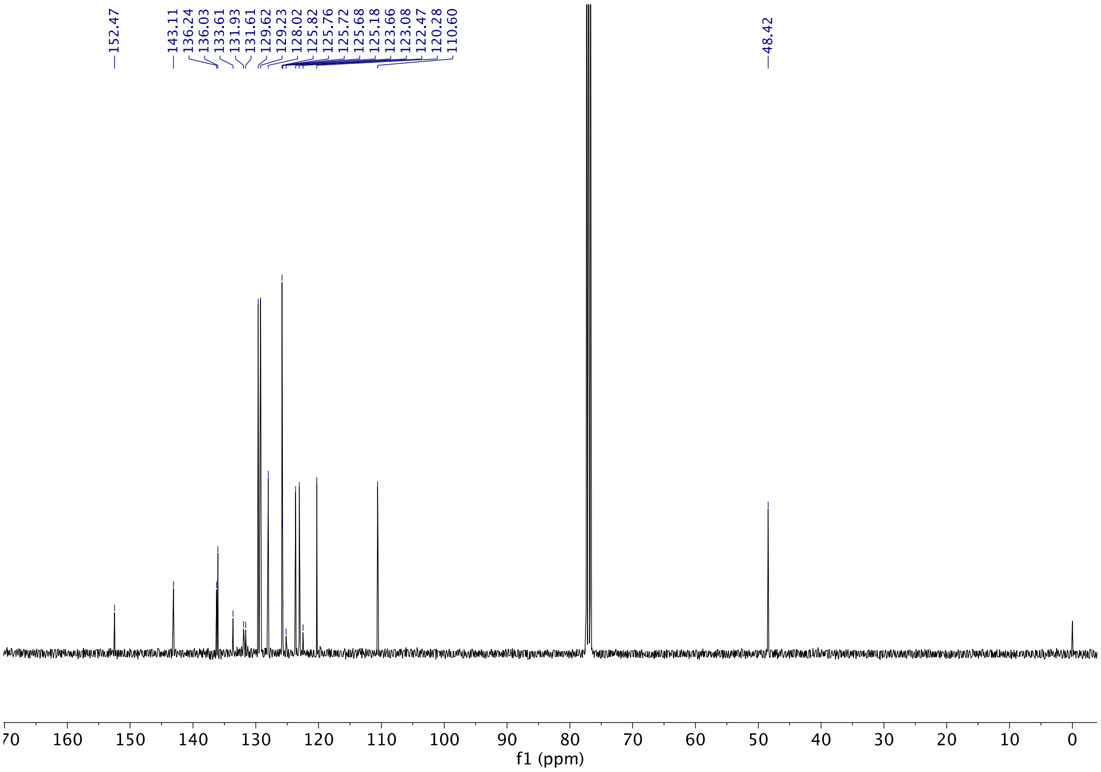

^{19}F spectrum for 1-Benzyl-2-(4-(trifluoromethyl)phenyl)-1*H*-benzo[*d*]imidazole (**30**)

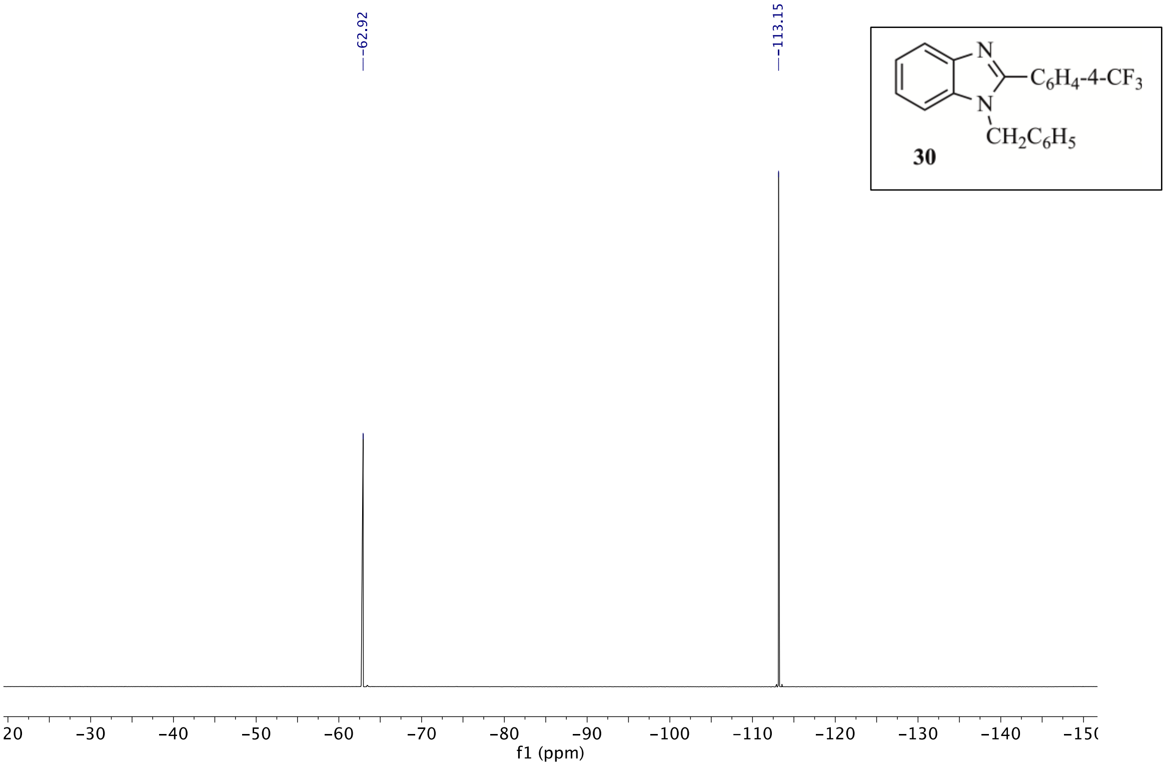

30

^{1}H and ^{13}C spectra for 2-Nitro-*N*-phenethylaniline (**7f**)

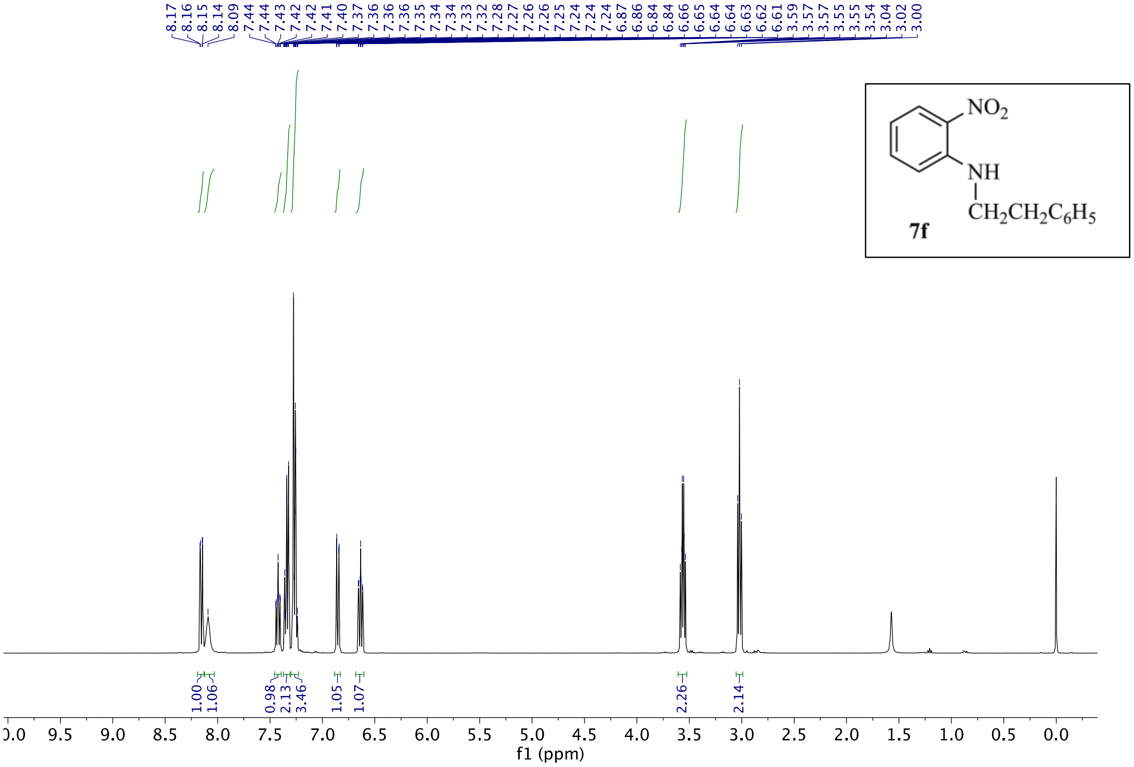

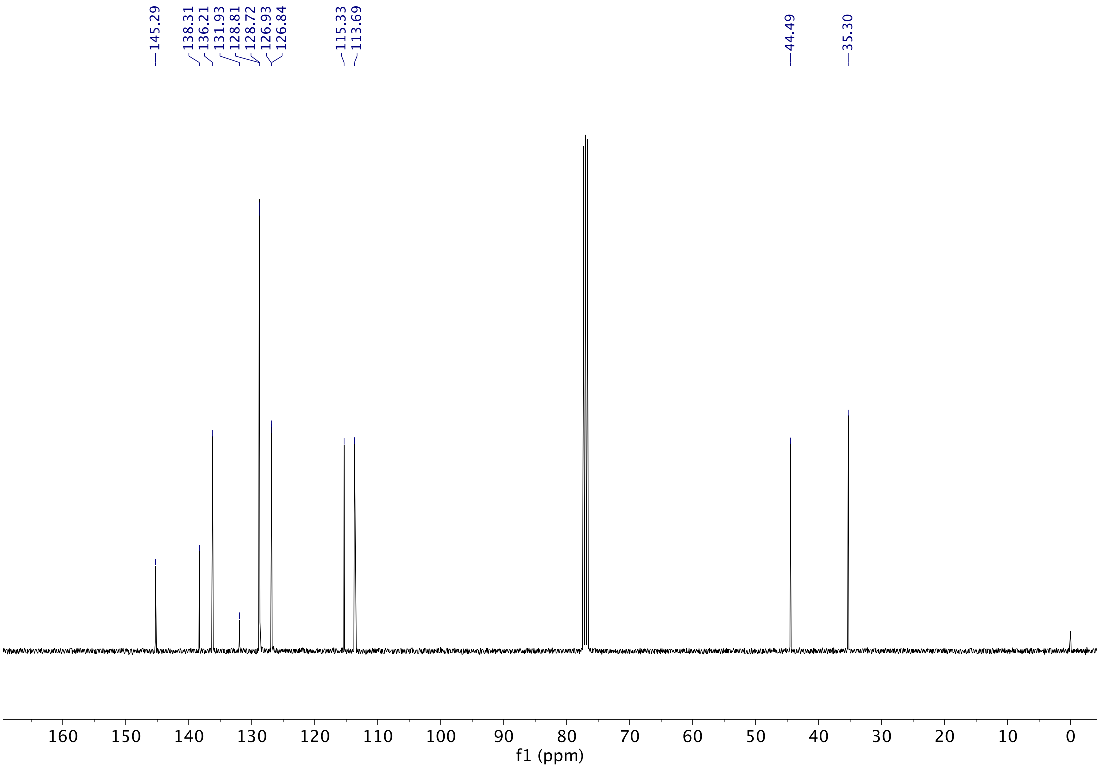

¹H and ¹³C spectra for 2-Methyl-1-phenethyl-1H-benzo[d]imidazole (**31**)

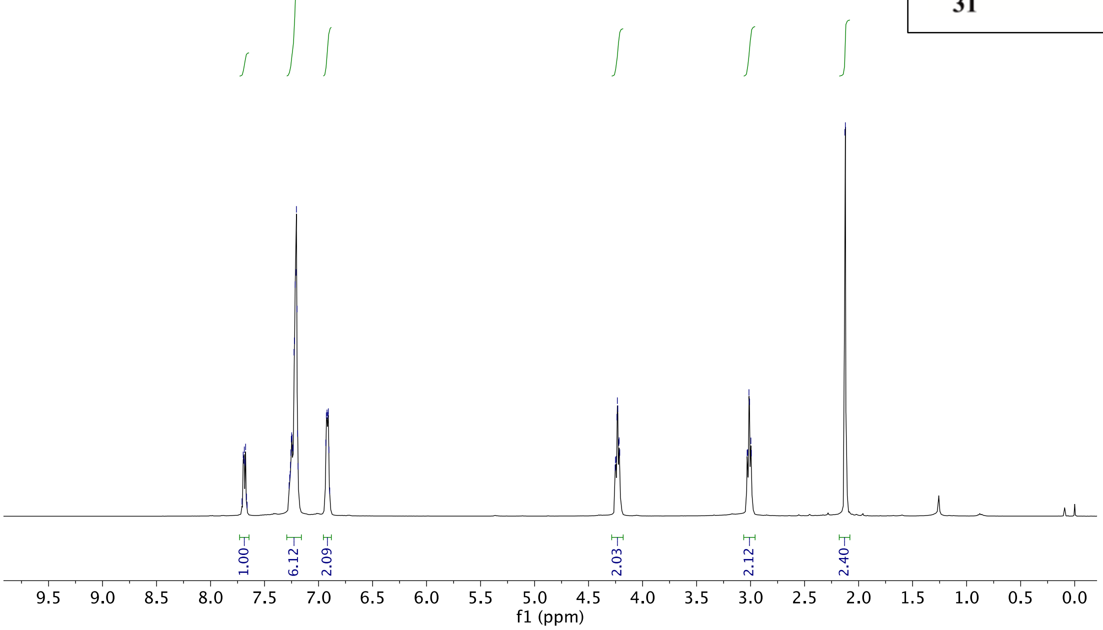

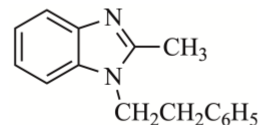

31

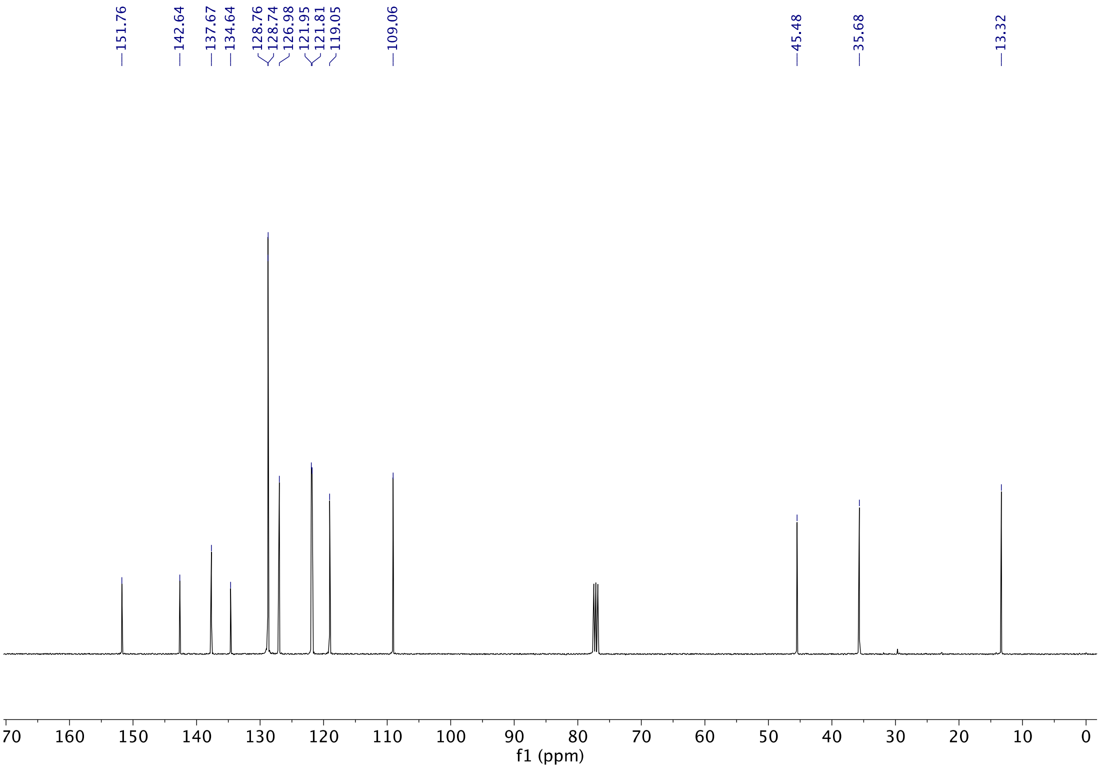

^{1}H and ^{13}C spectra for 2-Pentyl-1-phenethyl-1*H*-benzo[*d*]imidazole (**32**)

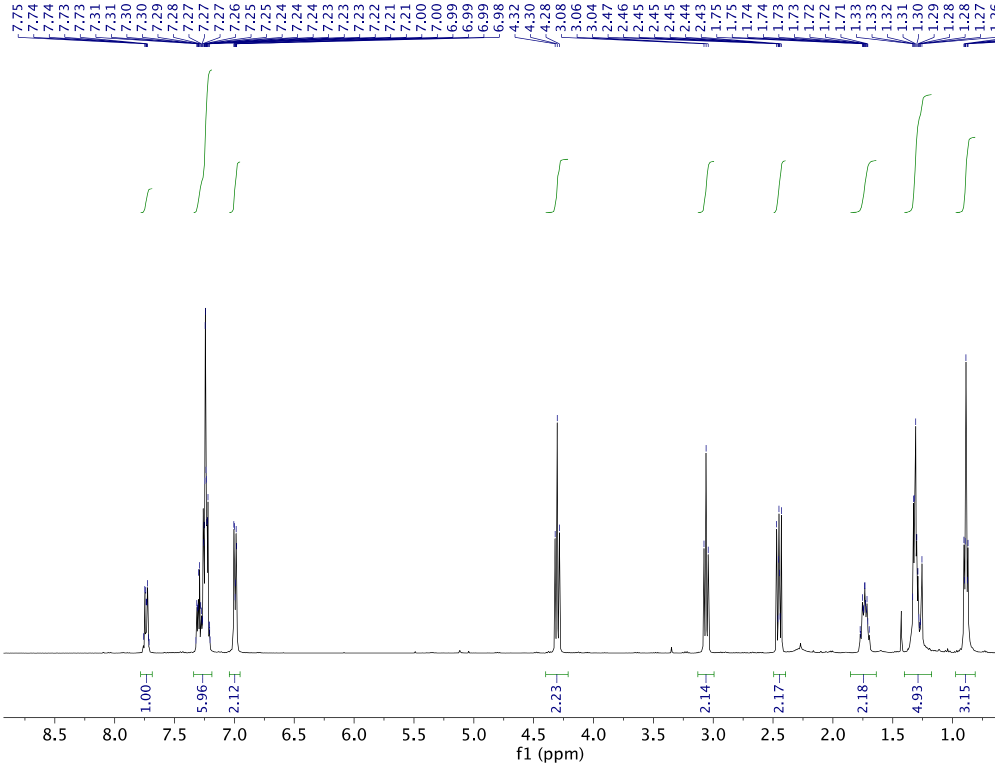

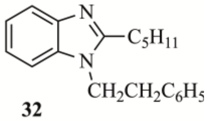

32

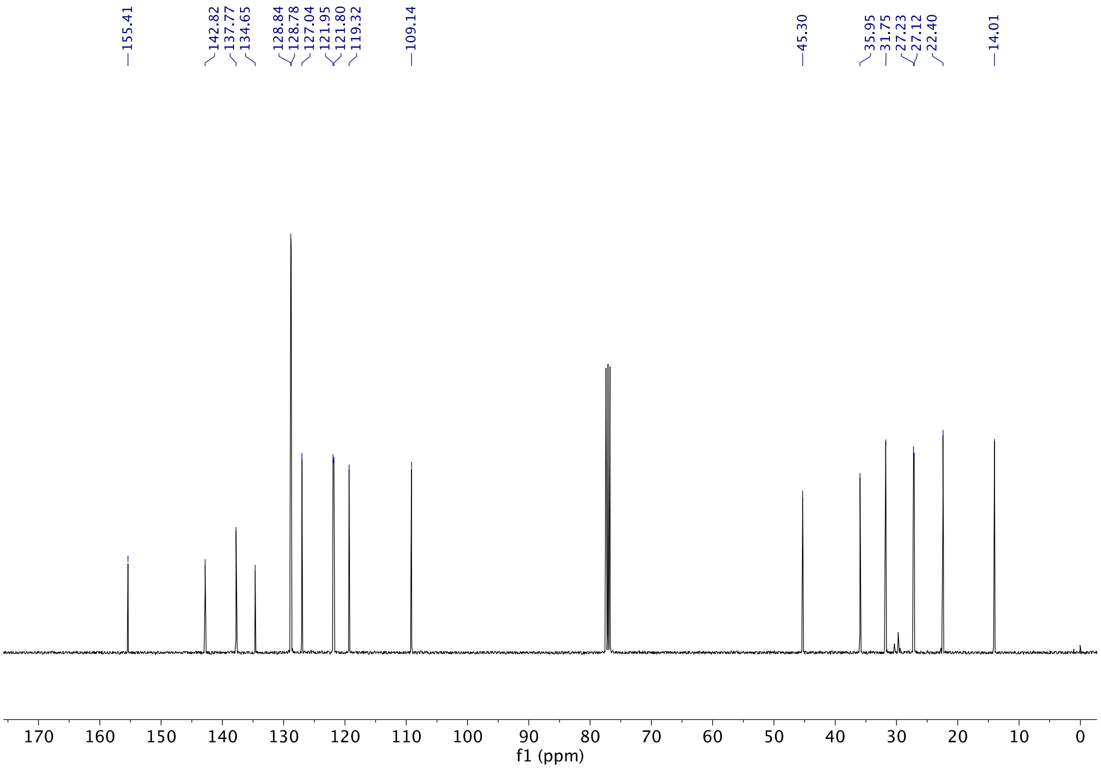

^{1}H and ^{13}C spectra for 2-Isopropyl-1-phenethyl-1*H*-benzo[*d*]imidazole (**33**)

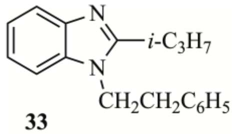

33

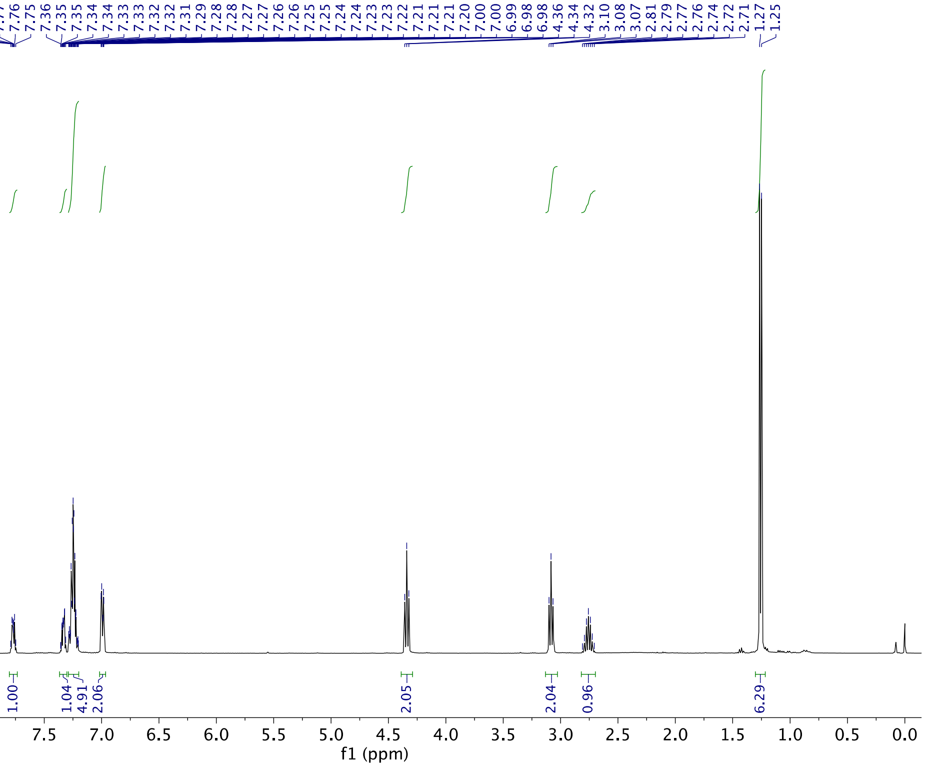

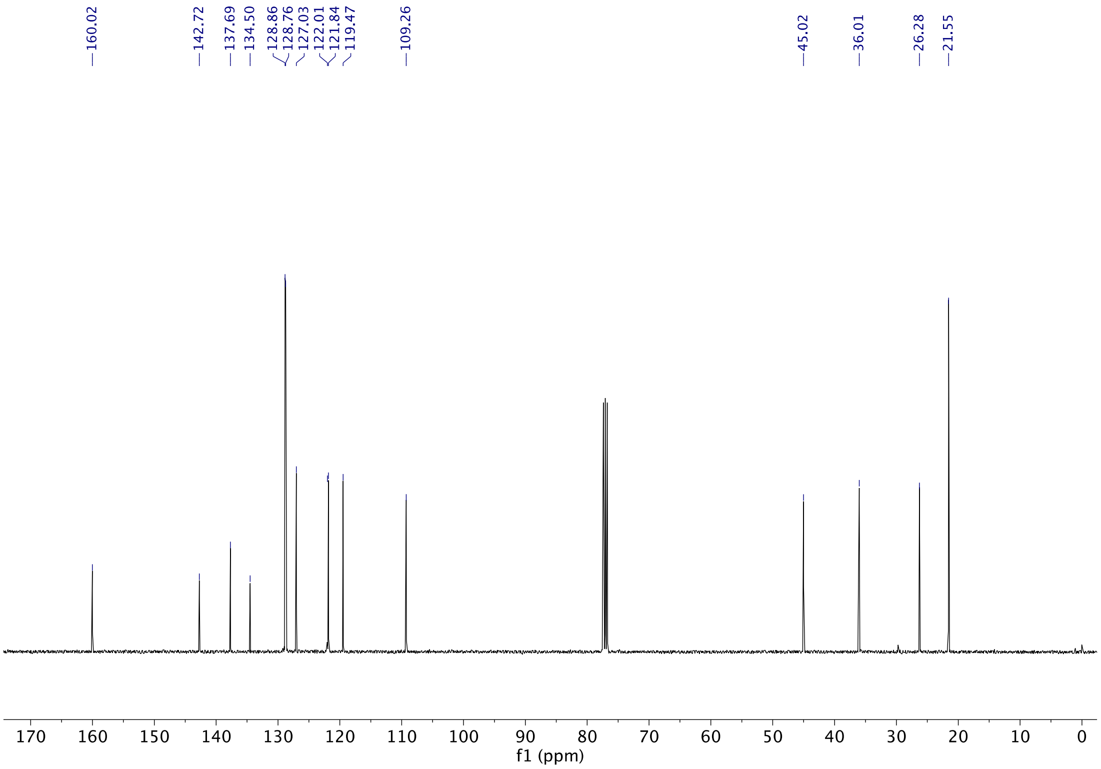

^1H and ^{13}C spectra for 2-(4-Chlorophenyl)-1-phenethyl-1*H*-benzo[*d*]imidazole (**34**)

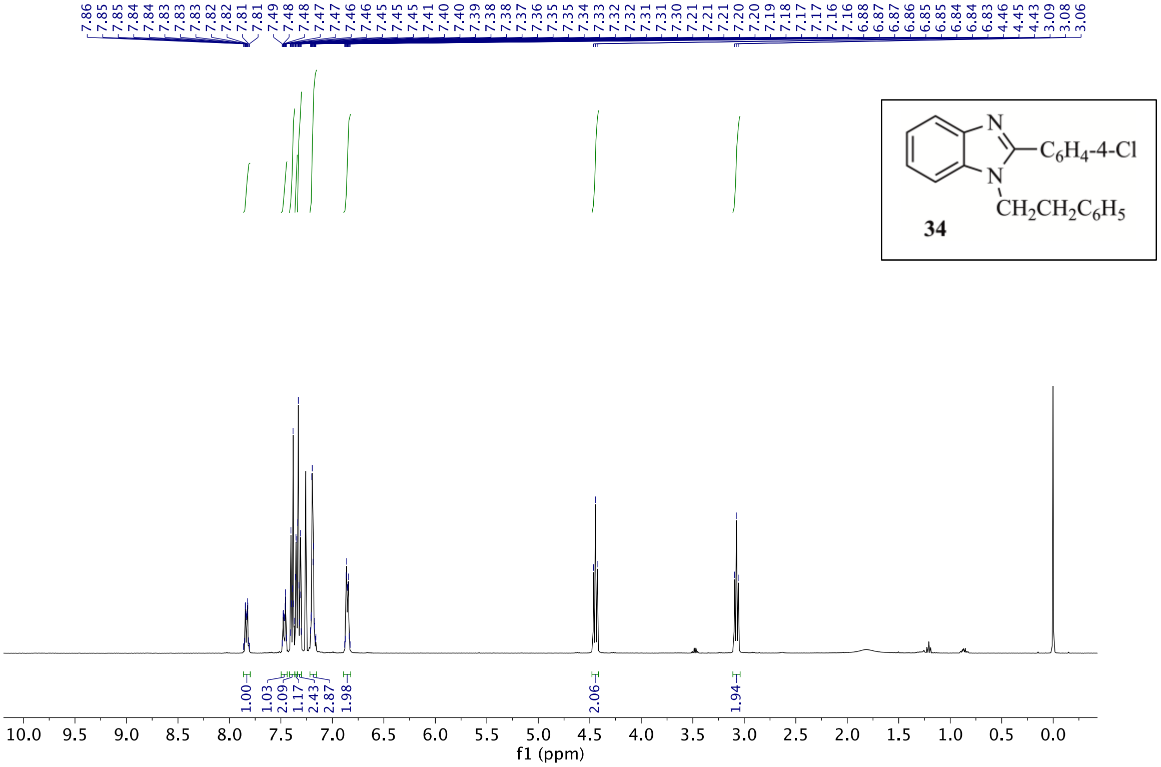

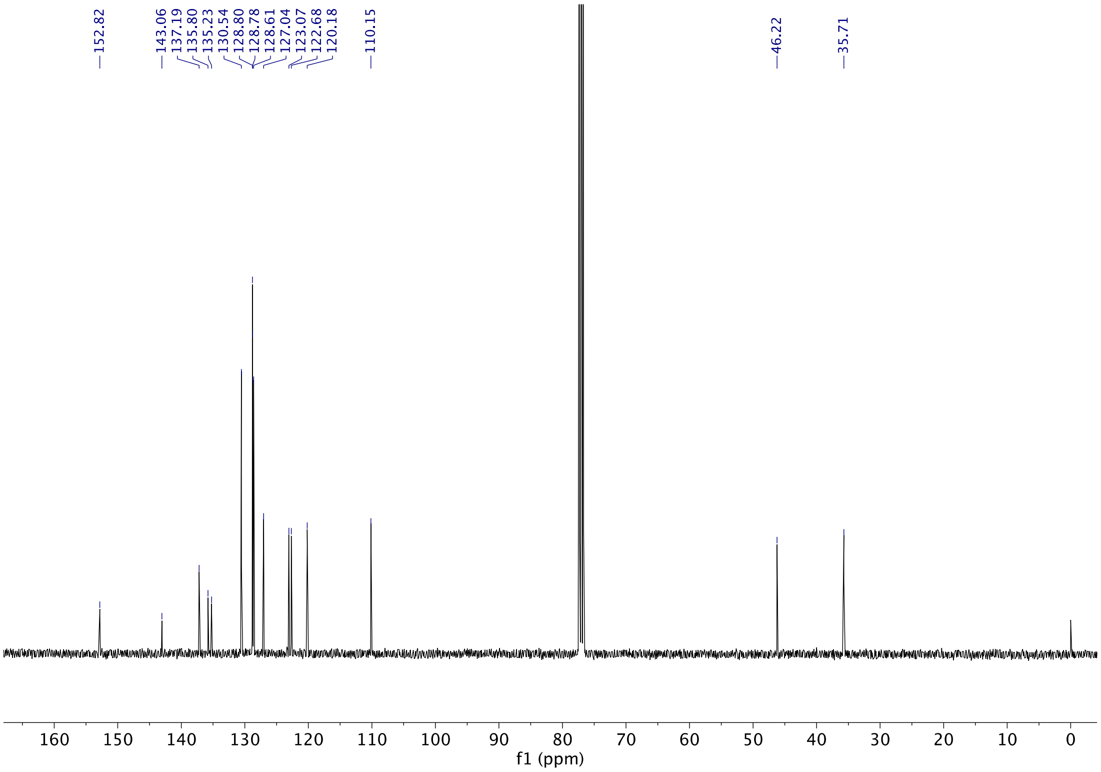

^{1}H and ^{13}C spectra for *N*-(4-Methoxybenzyl)-2-nitroaniline (**7g**)

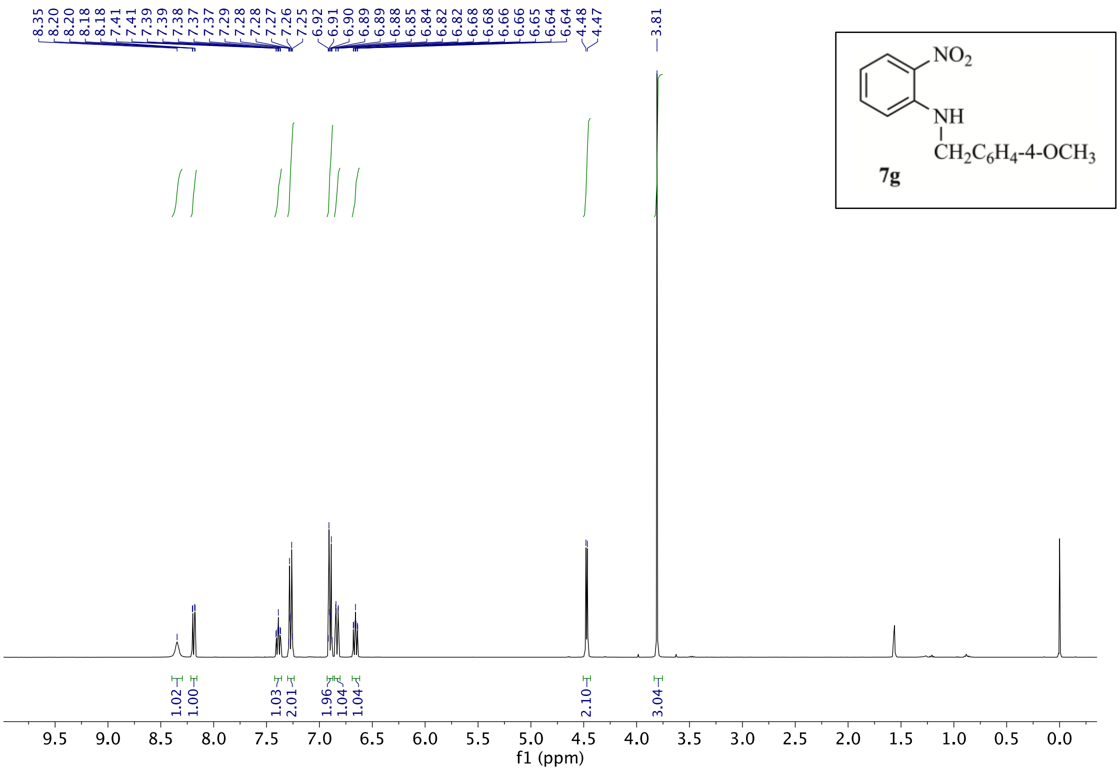

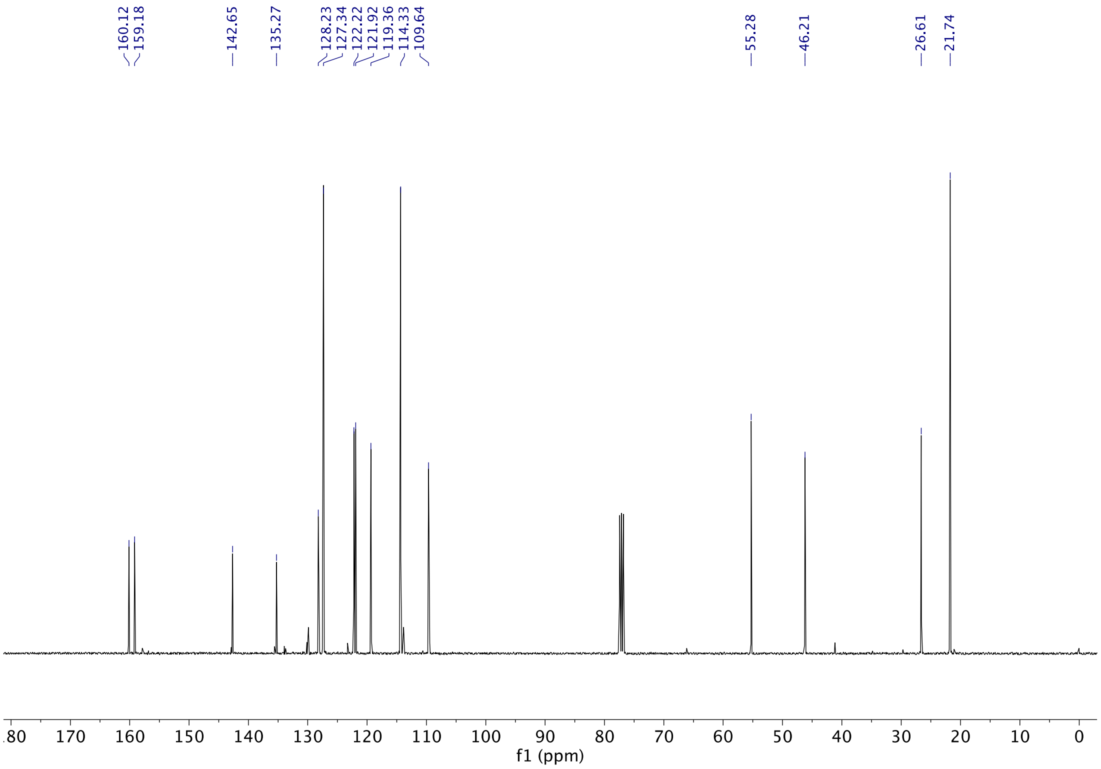

^{1}H and ^{13}C spectra for 2-Isopropyl-1-(4-methoxybenzyl)-1*H*-benzo[*d*]imidazole (**35**)

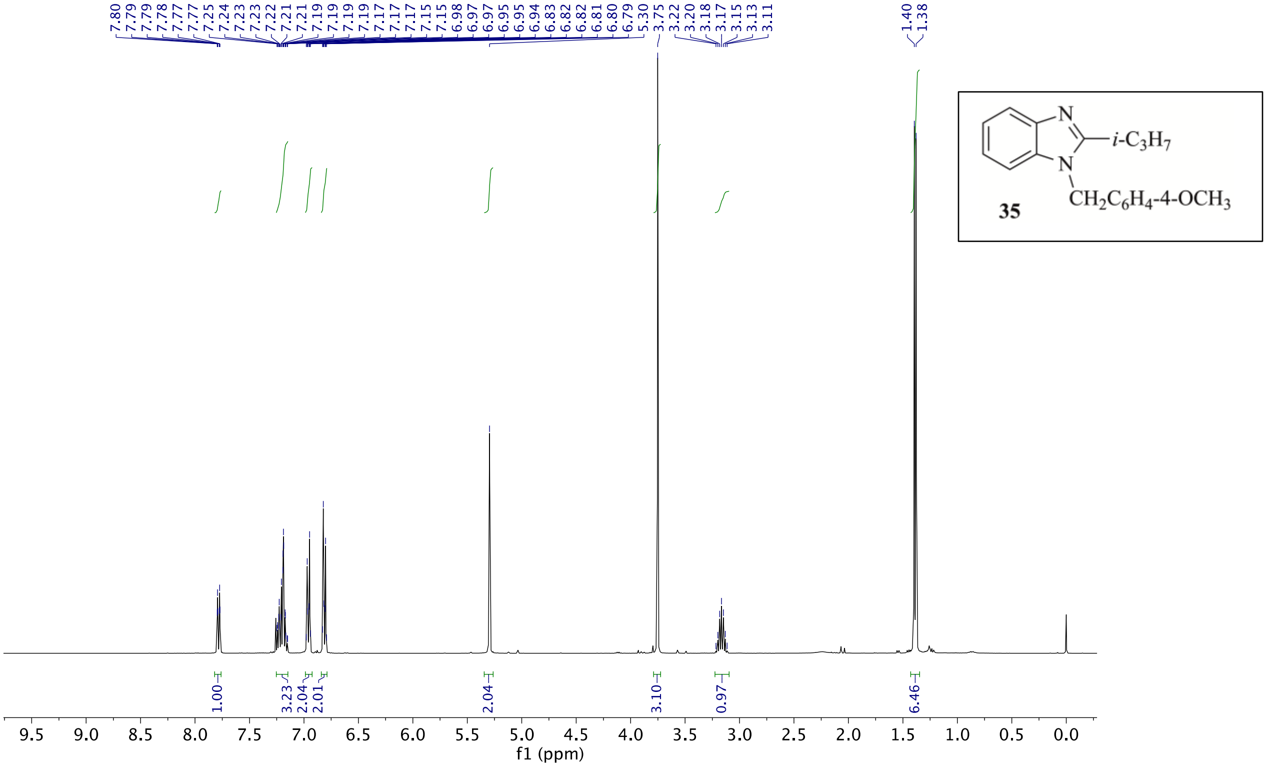

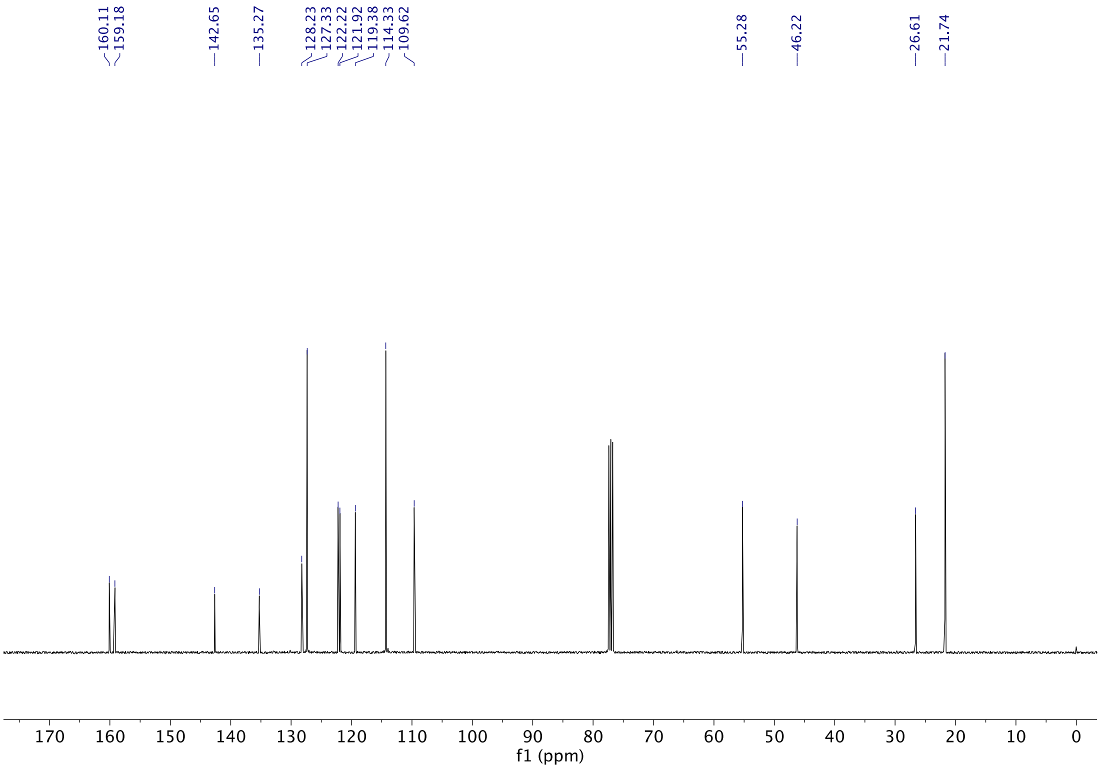

^{1}H and ^{13}C spectra for 1-(4-Methoxybenzyl)-2-phenyl-1*H*-benzo[*d*]imidazole (**36**)

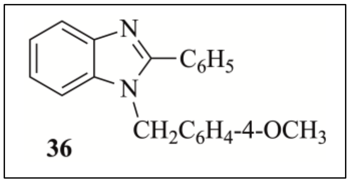

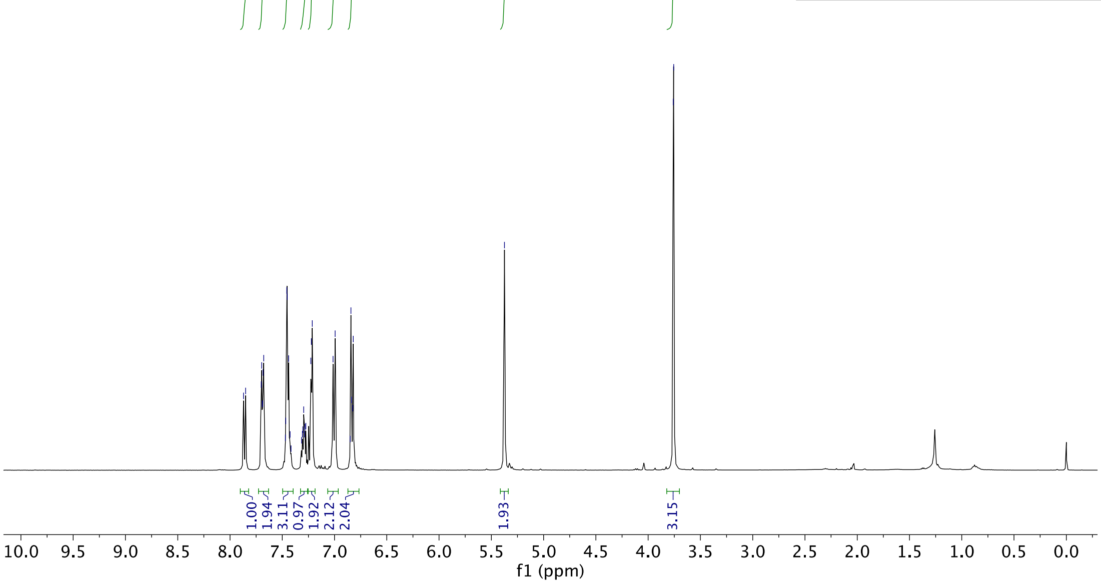

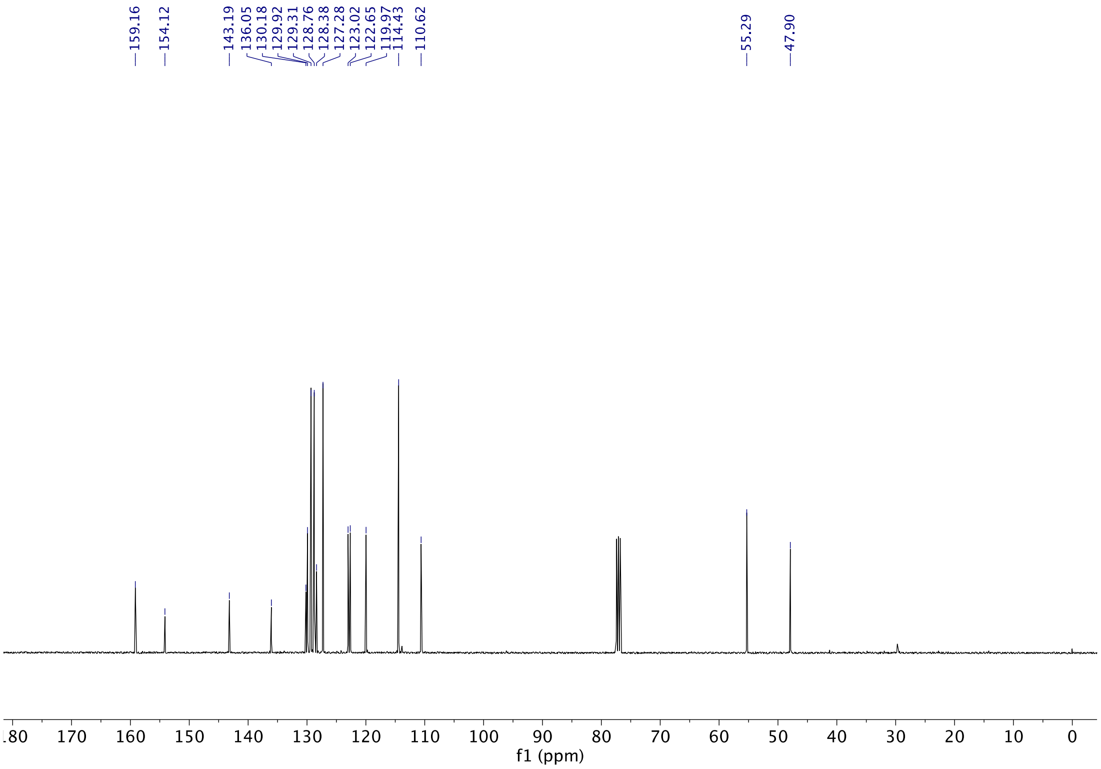

^{1}H and ^{13}C spectra for 2-(2-Fluorophenyl)-1-(4-methoxyphenyl)-1*H*-benzo[*d*]imidazole (**37**)

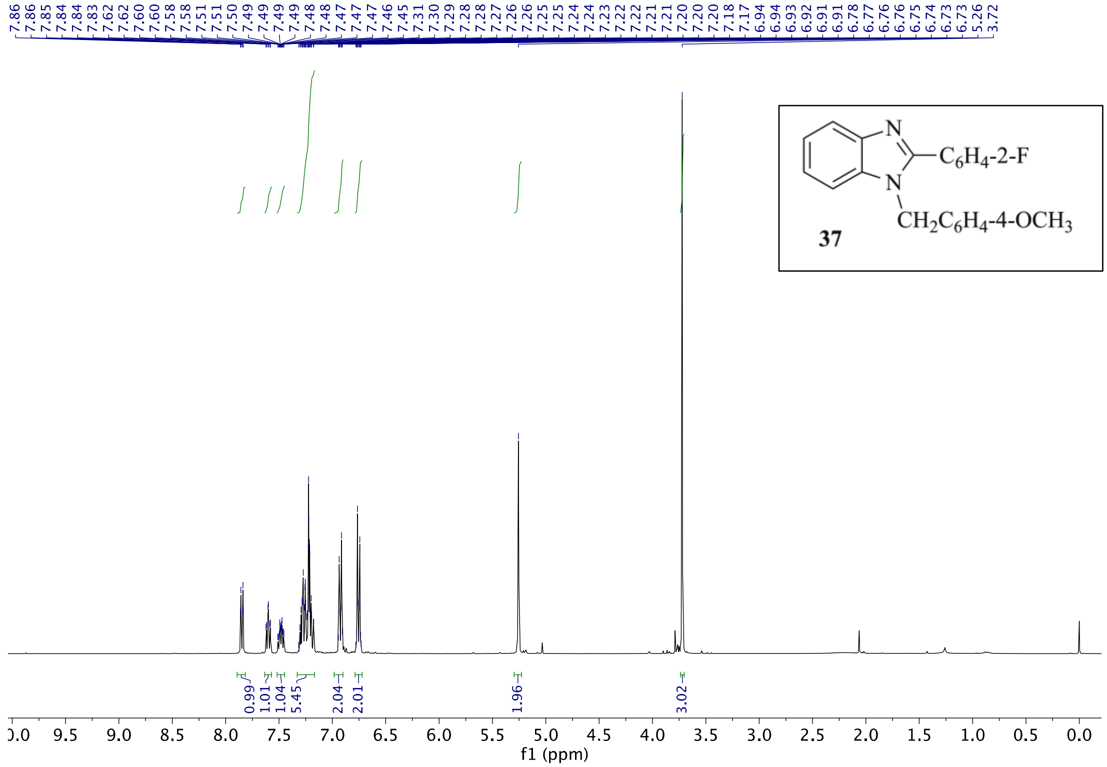

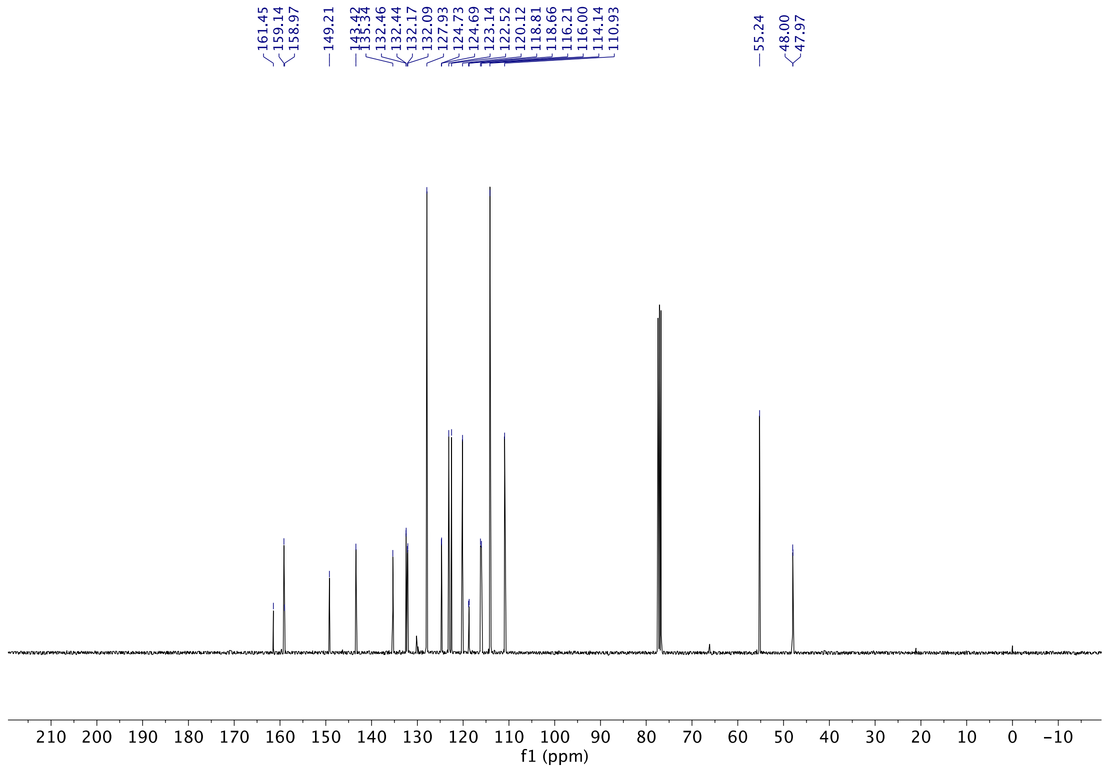

^{19}F spectrum for 2-(2-Fluorophenyl)-1-(4-methoxyphenyl)-1*H*-benzo[*d*]imidazole (**37**)

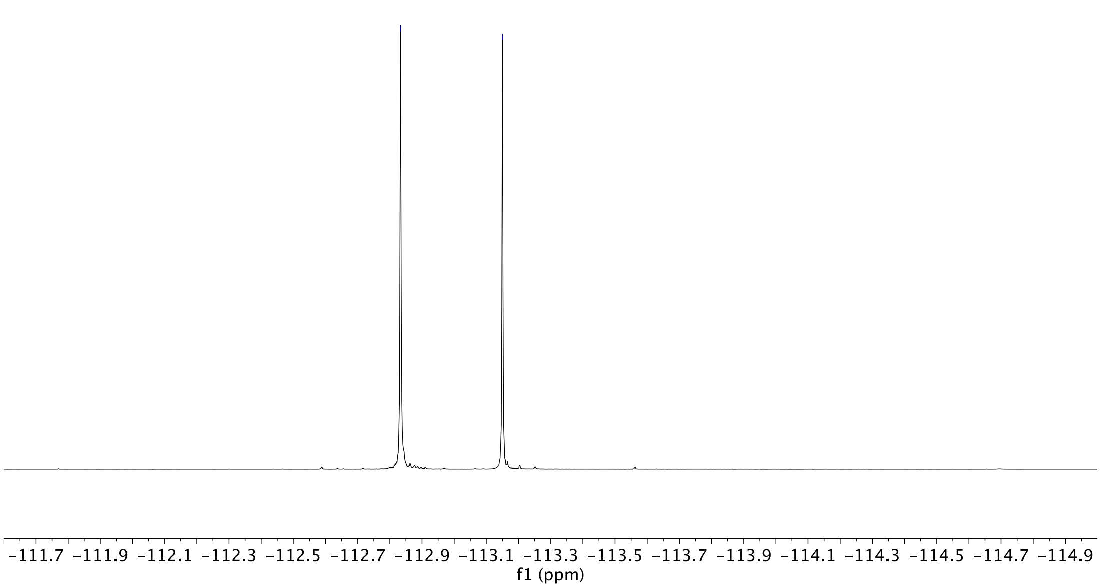

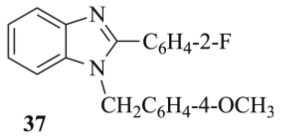

37

^{1}H and ^{13}C spectra for

2-(4-Fluoro-3-methylphenyl)-1-(4-methoxybenzyl)-1*H*-benzo[*d*]imidazole (**38**)

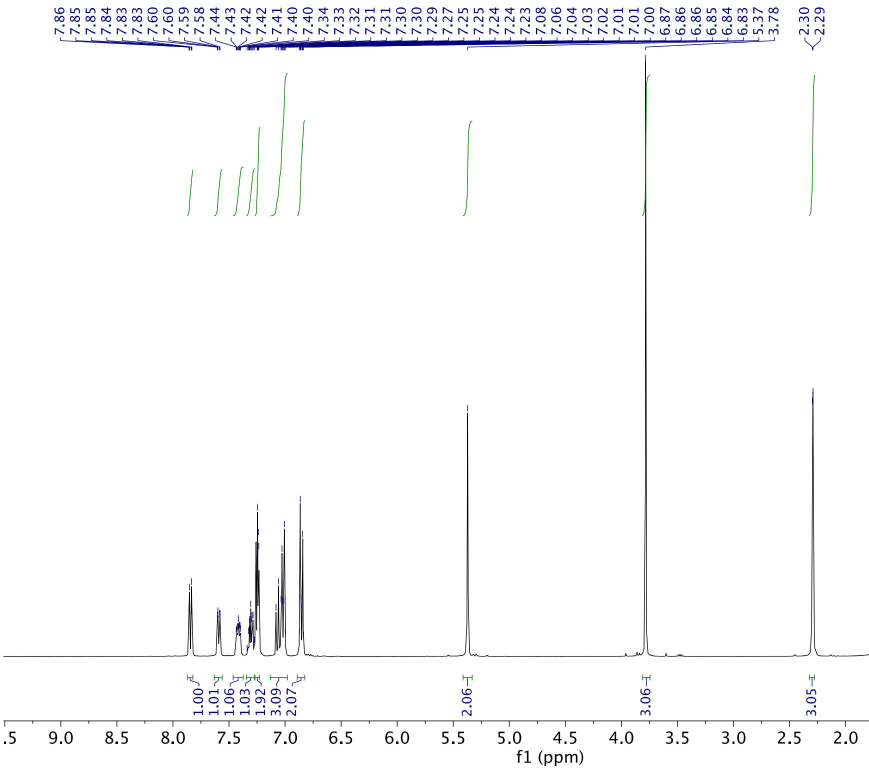

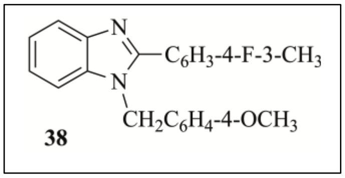

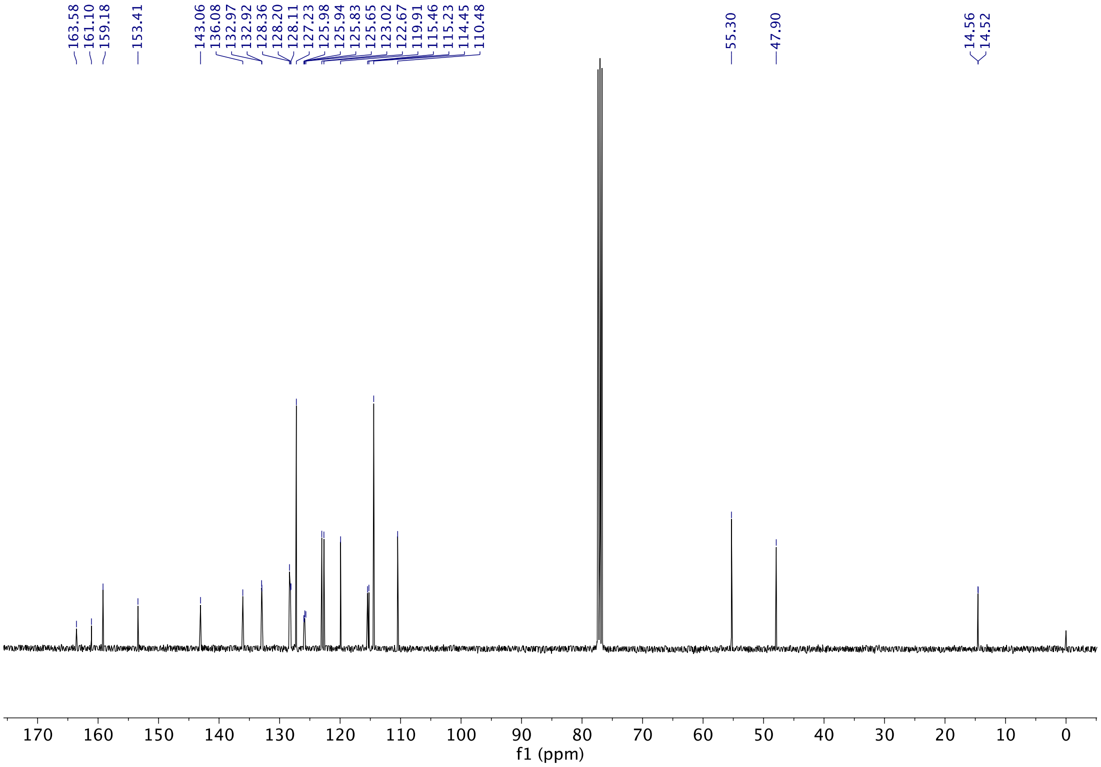

^{19}F spectrum for 2-(4-Fluoro-3-methylphenyl)-1-(4-methoxybenzyl)-1*H*-benzo[*d*]imidazole (**38**)

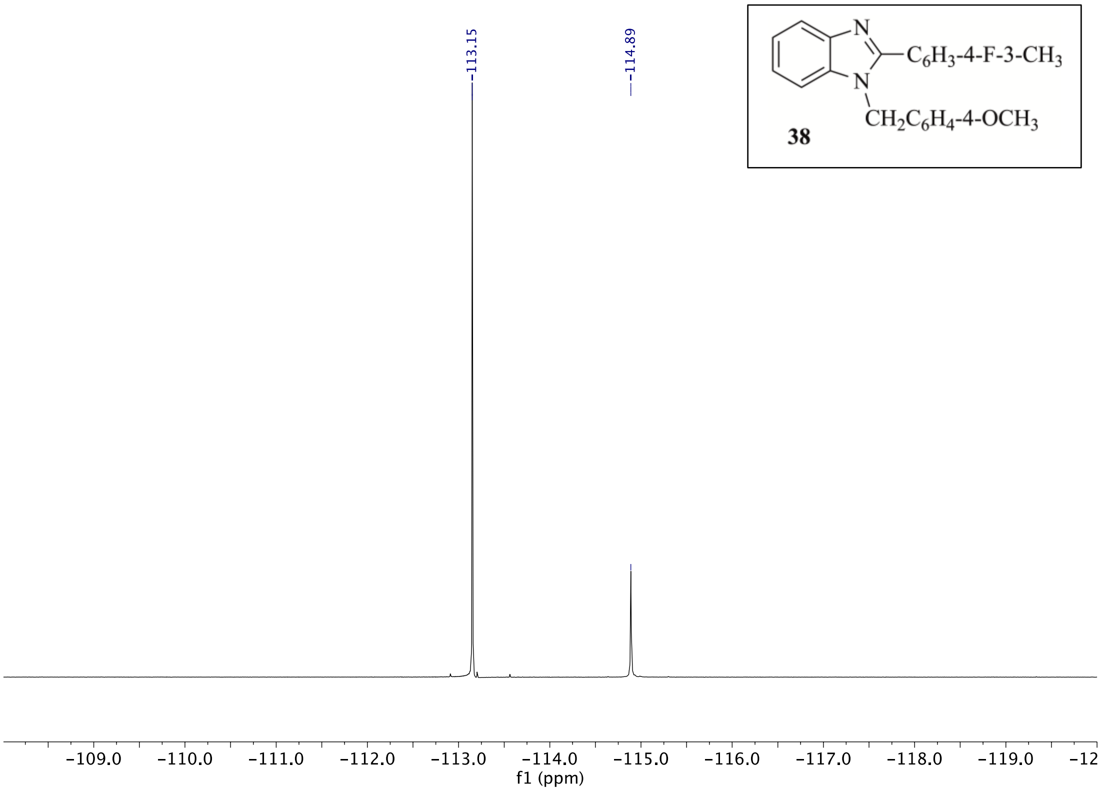

¹H and ¹³C spectra for 2-Nitro-*N*-(4-(trifluoromethyl)benzyl)aniline (**7h**)

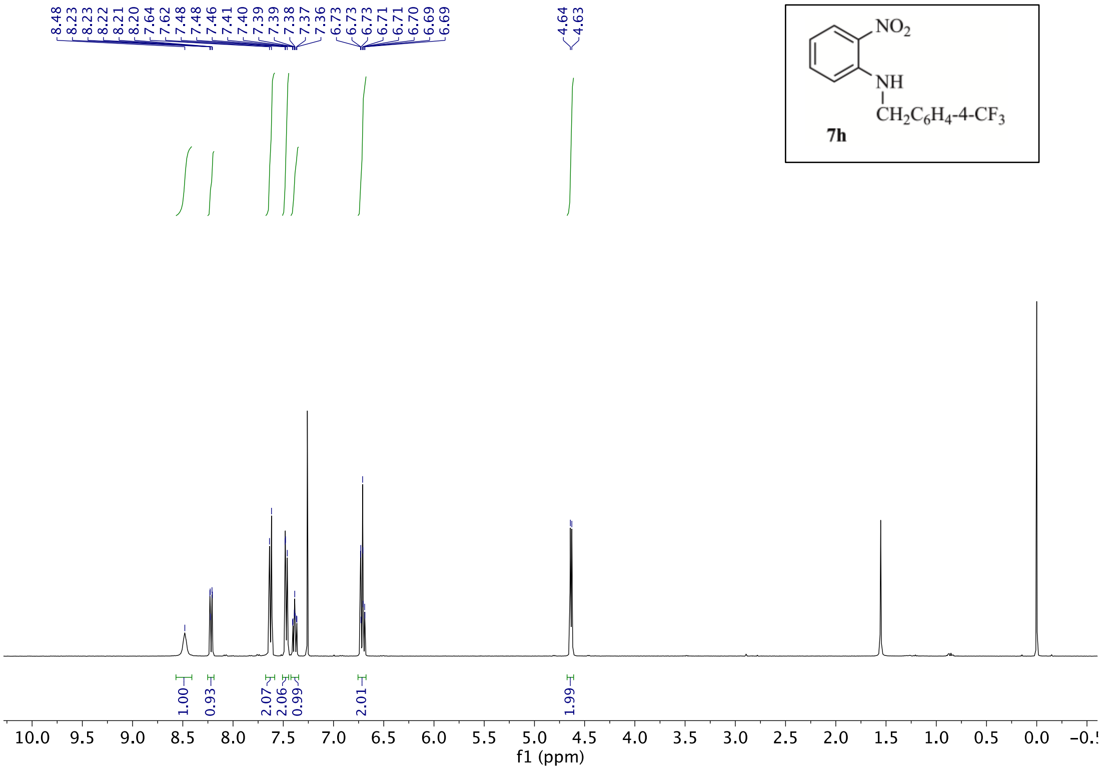

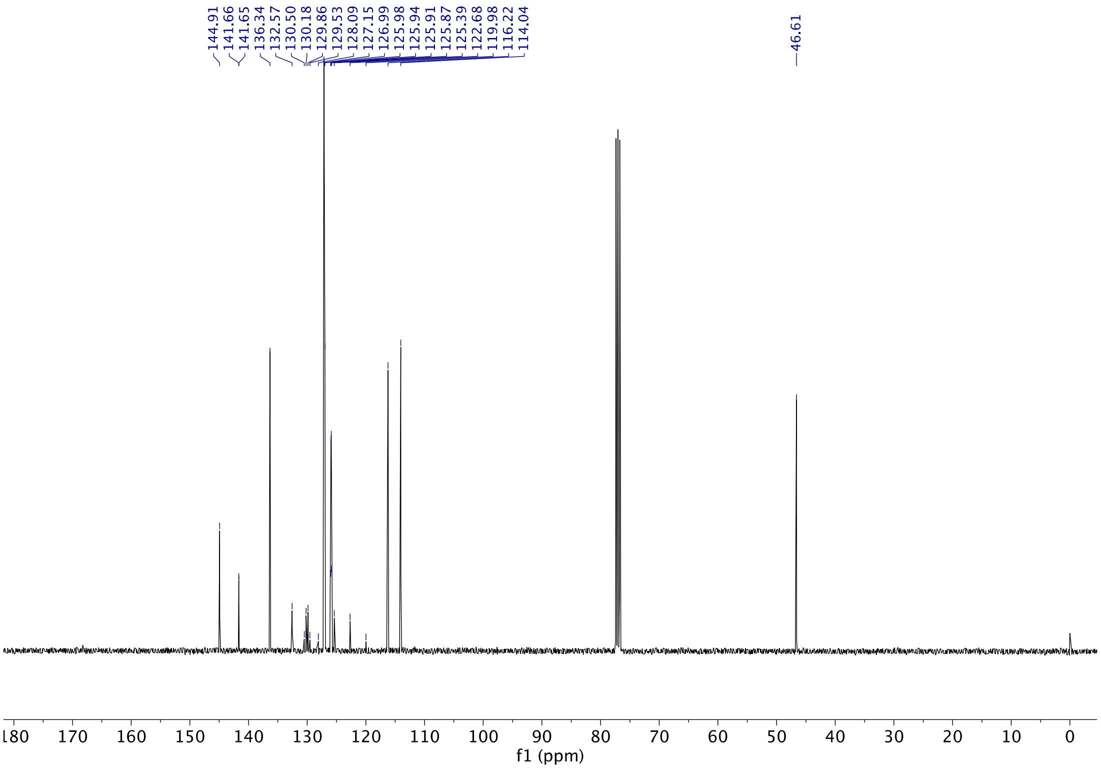

^{19}F spectrum for 2-Nitro-*N*-(4-(trifluoromethyl)benzyl)aniline (**7h**)

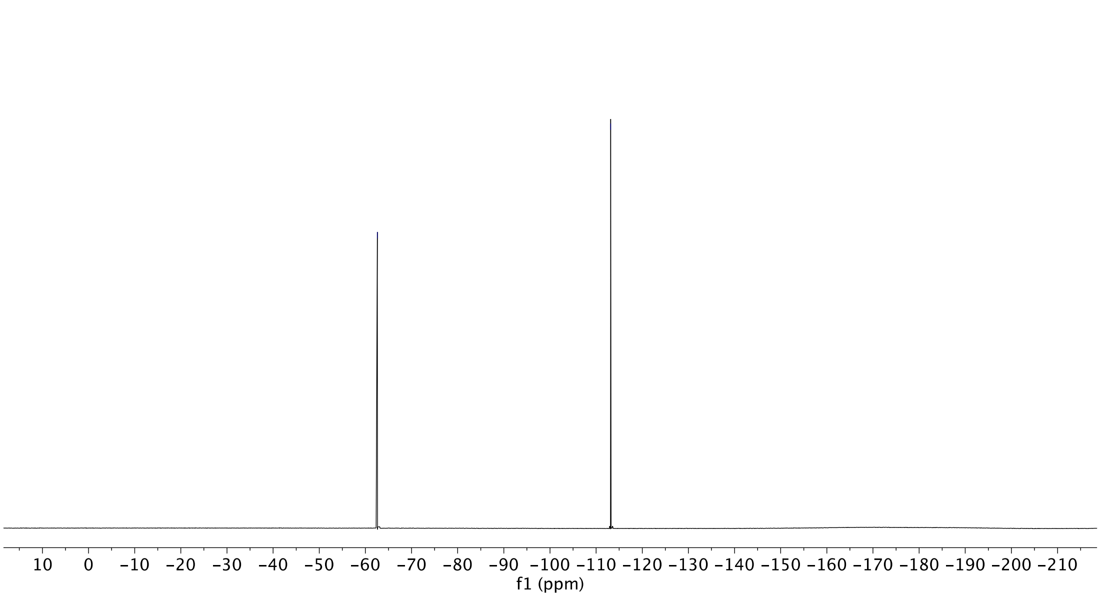

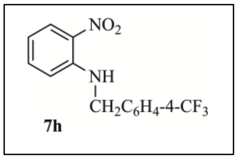

^{1}H and ^{13}C spectra for 2-Isopropyl-1-(4-(trifluoromethyl)benzyl)-1*H*-benzo[*d*]imidazole (**39**)

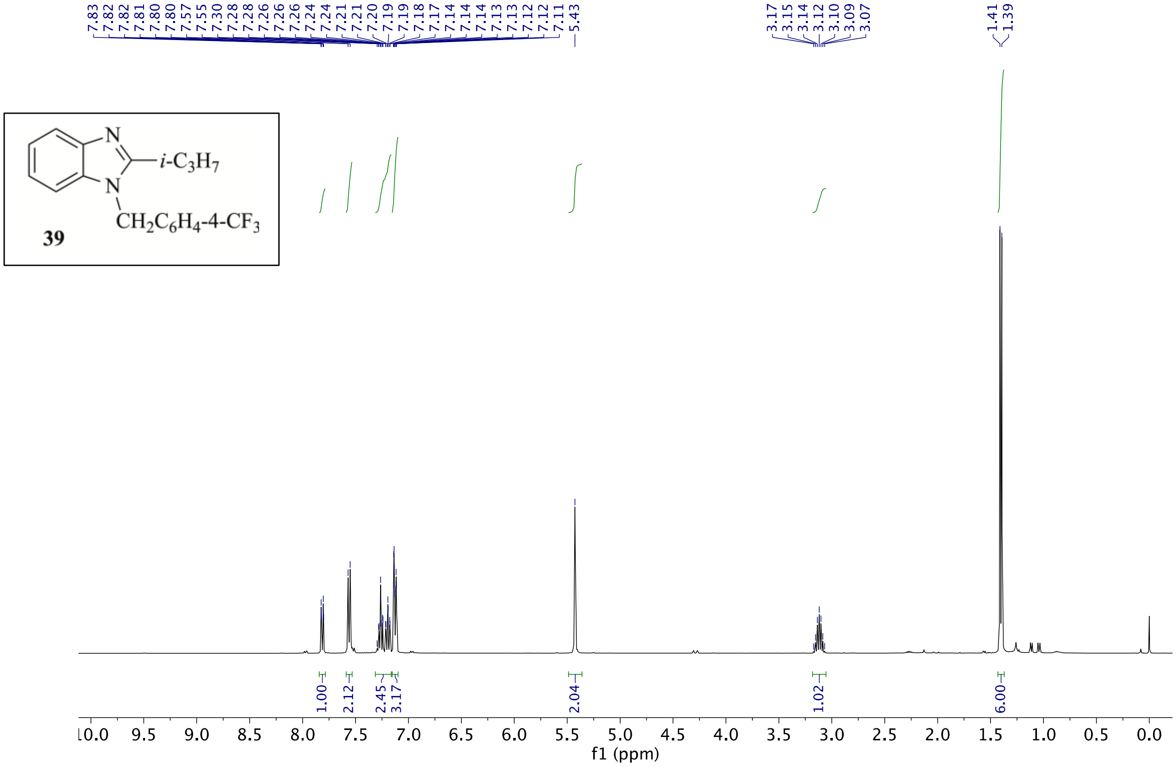

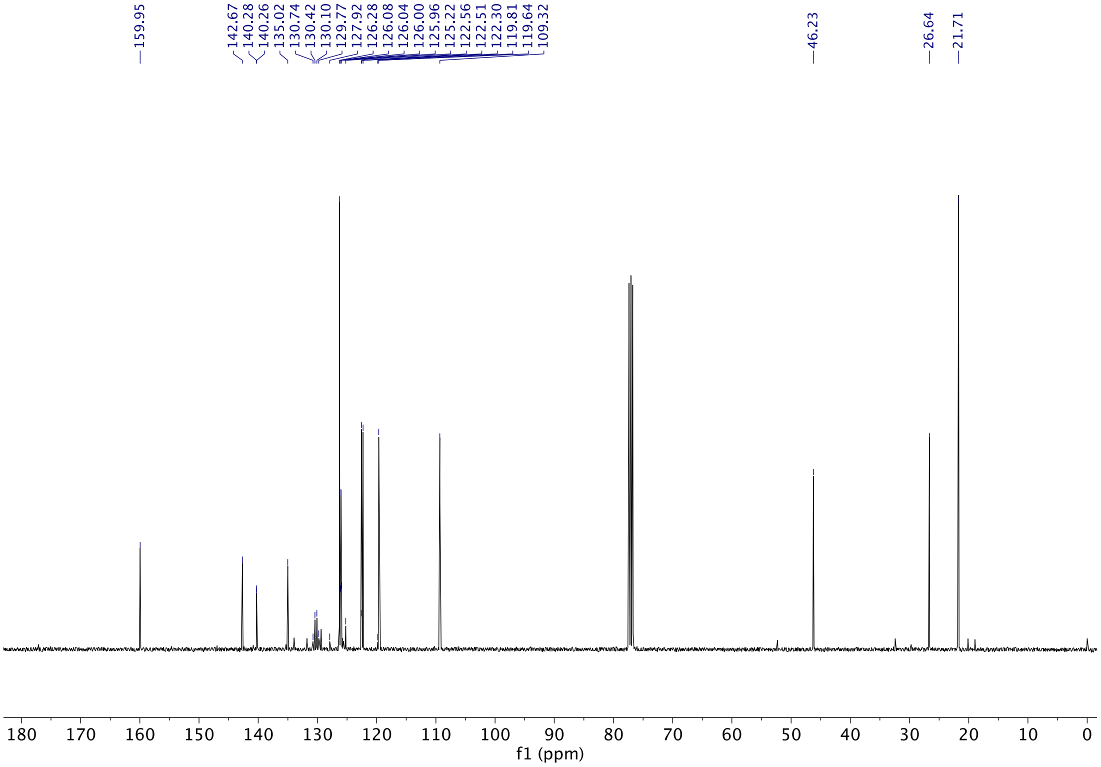

^{19}F spectrum for 2-Isopropyl-1-(4-(trifluoromethyl)benzyl)-1*H*-benzo[*d*]imidazole (**39**)

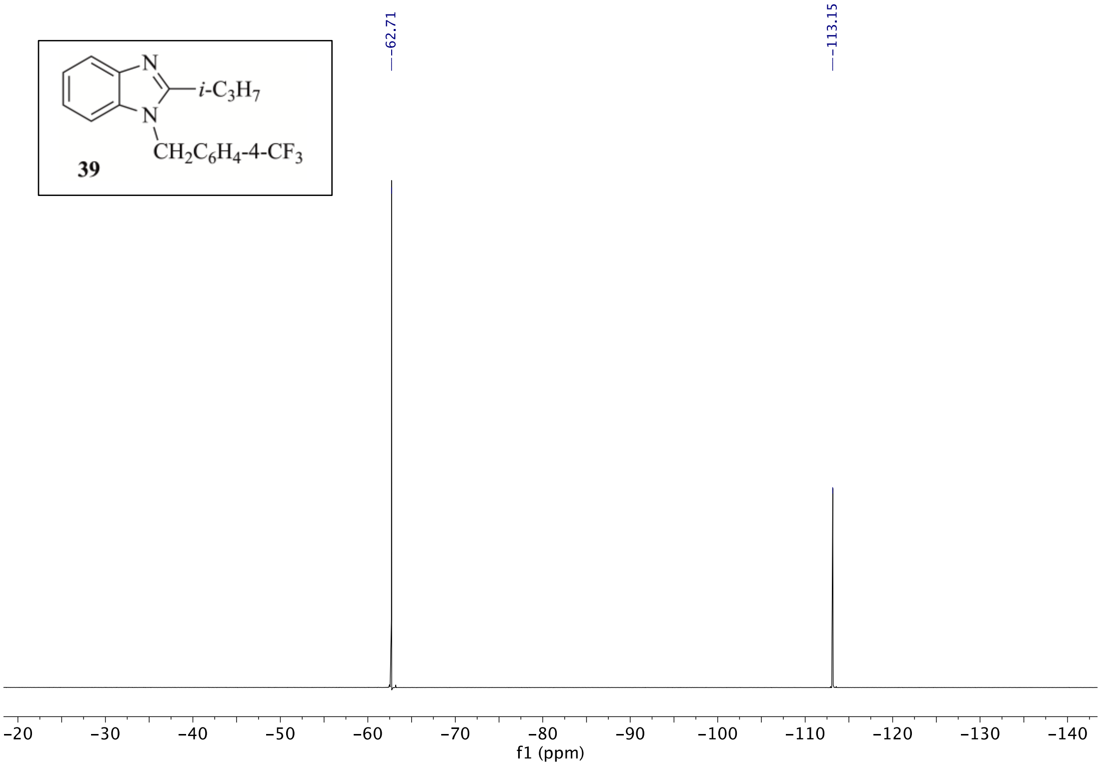

^{1}H and ^{13}C spectra for

2-(4-Methoxyphenyl)-1-(4-trifluoromethyl)benzyl)-1*H*-benzo[*d*]imidazole (**40**)

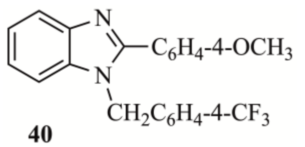

40

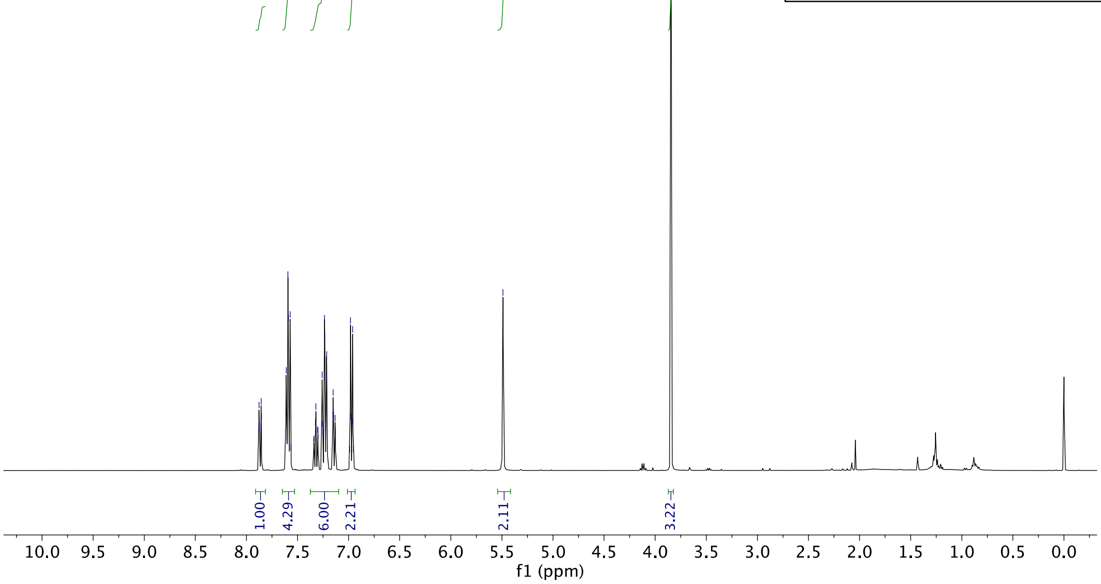

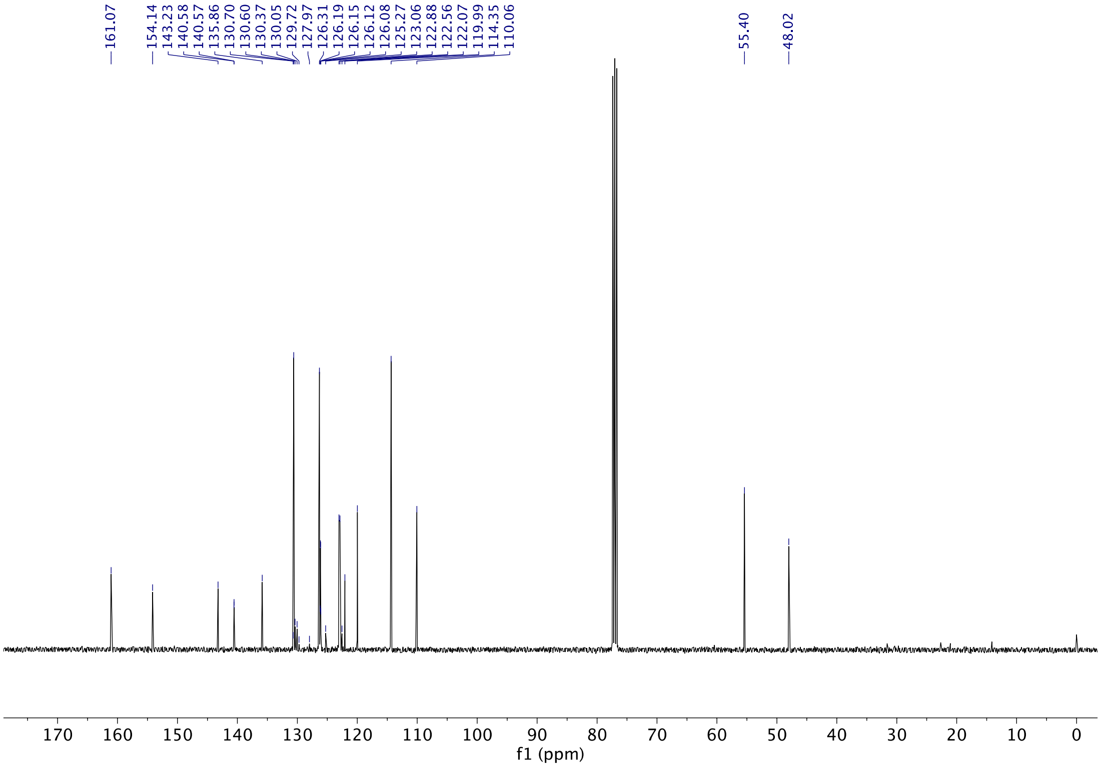

^{19}F spectrum for 2-(4-Methoxyphenyl)-1-(4-trifluoromethyl)benzyl)-1*H*-benzo[*d*]imidazole (**40**)

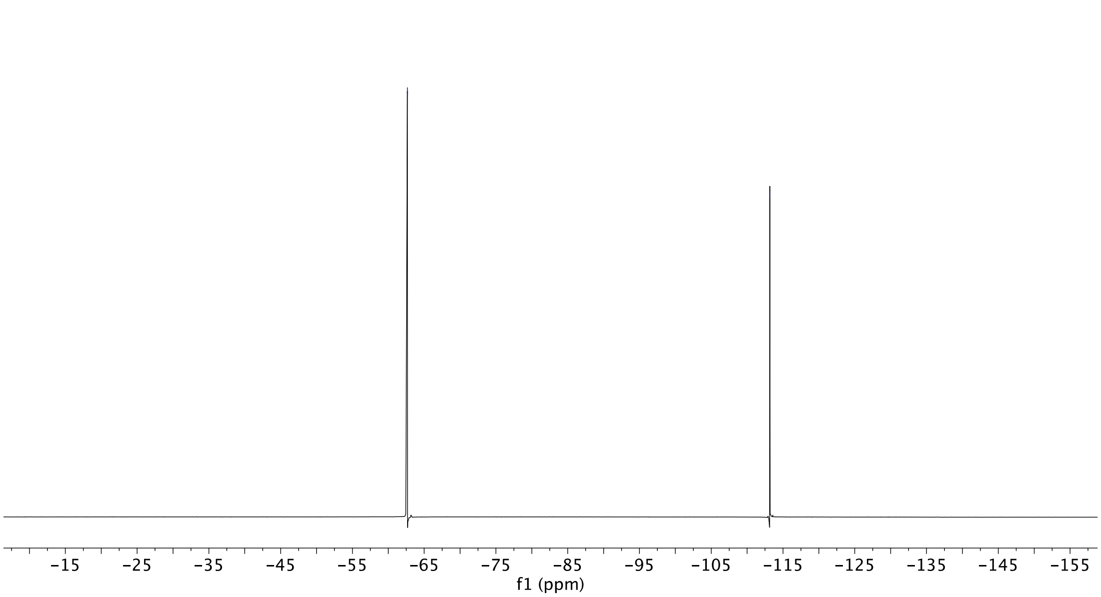

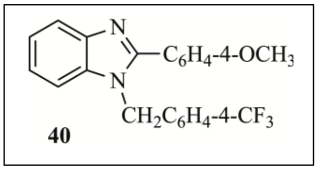

^{1}H and ^{13}C spectra for

2-(3-Chlorophenyl)-1-(4-(trifluoromethyl)benzyl)-1*H*-benzo[*d*]imidazole (**41**)

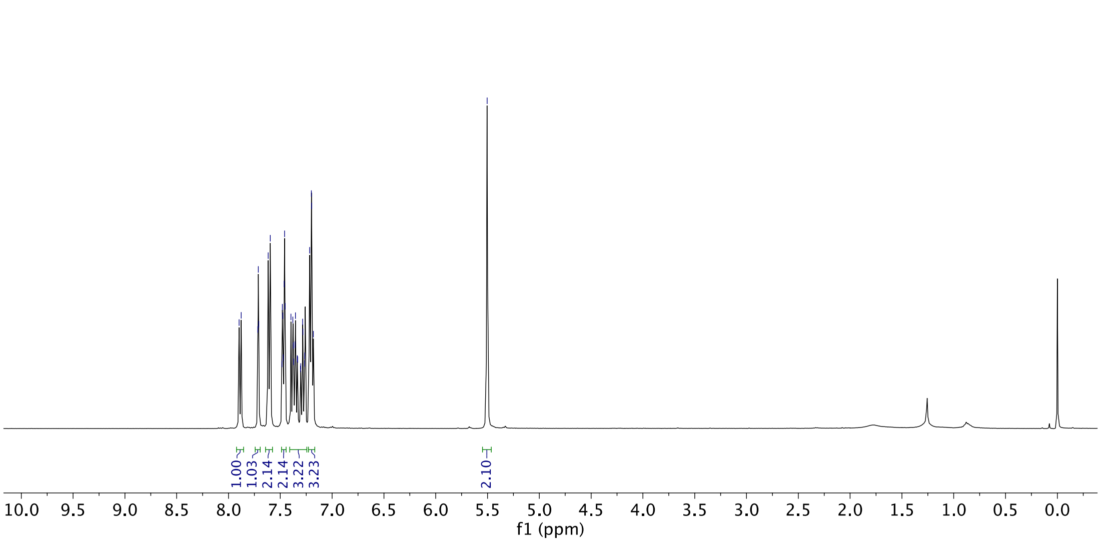

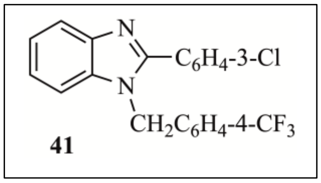

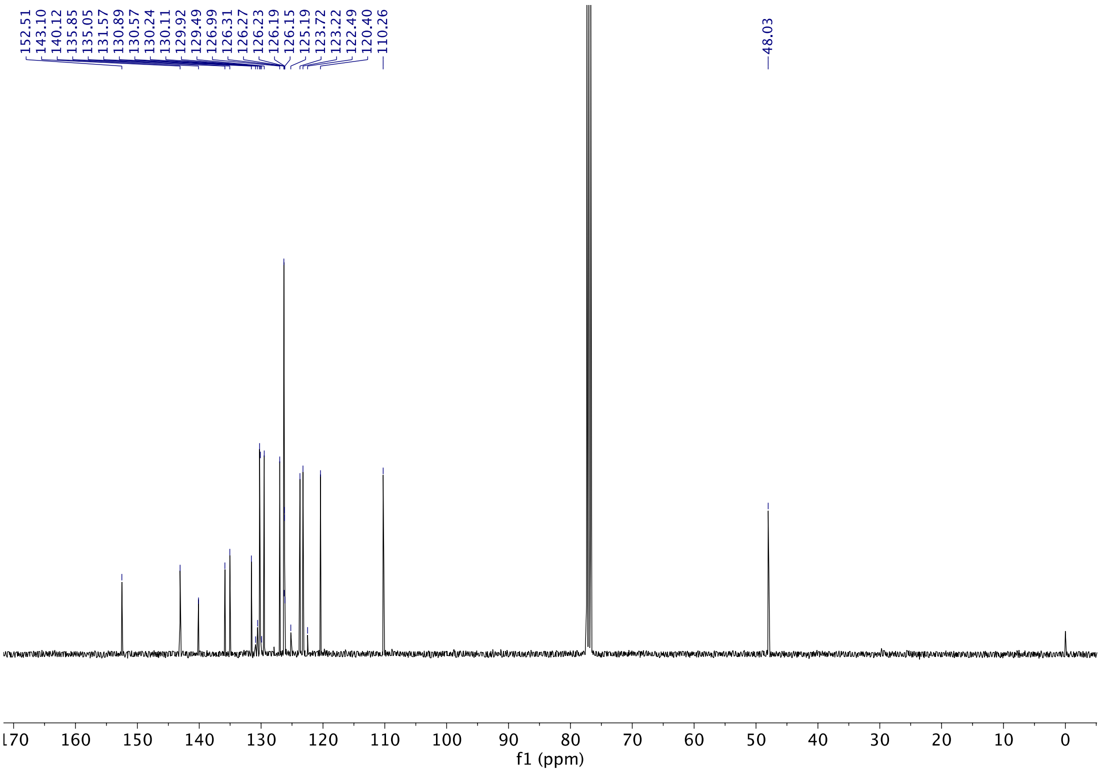

^{19}F spectrum for 2-(3-Chlorophenyl)-1-(4-(trifluoromethyl)benzyl)-1*H*-benzo[*d*]imidazole (**41**)

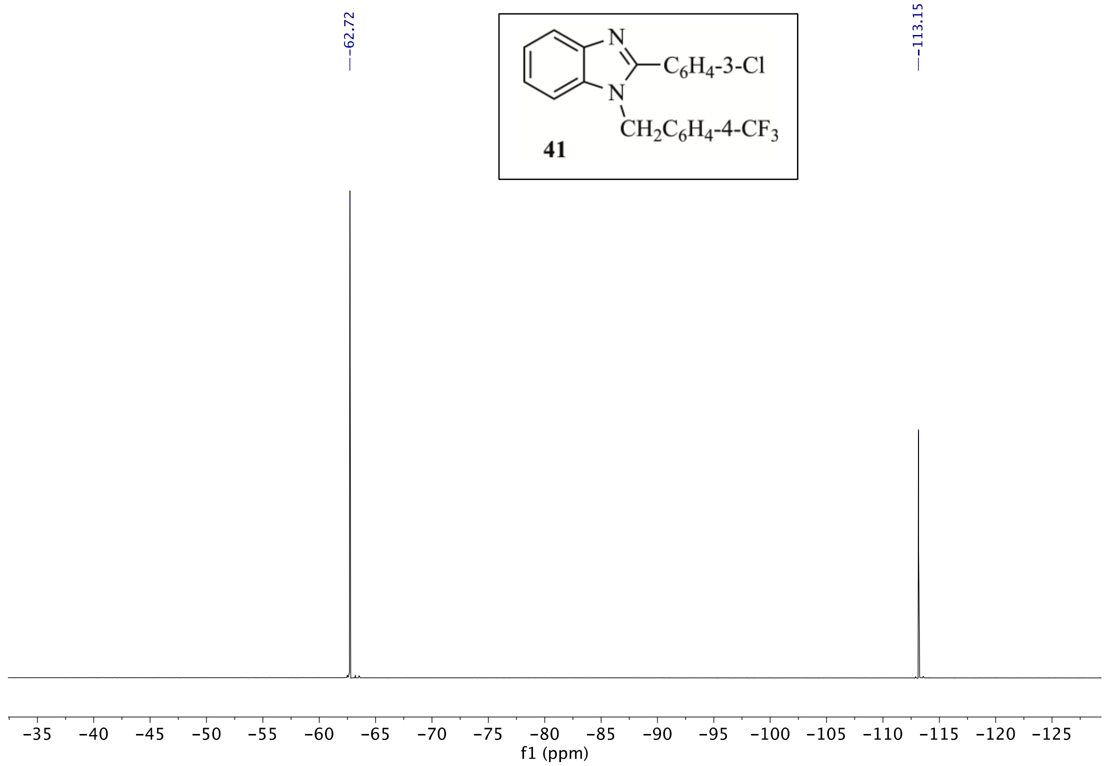

^{1}H and ^{13}C spectra for 2-(2-Fluorophenyl)-1-(4-(trifluoromethyl)benzyl)-1*H*-benzo[*d*]imidazole (**42**)

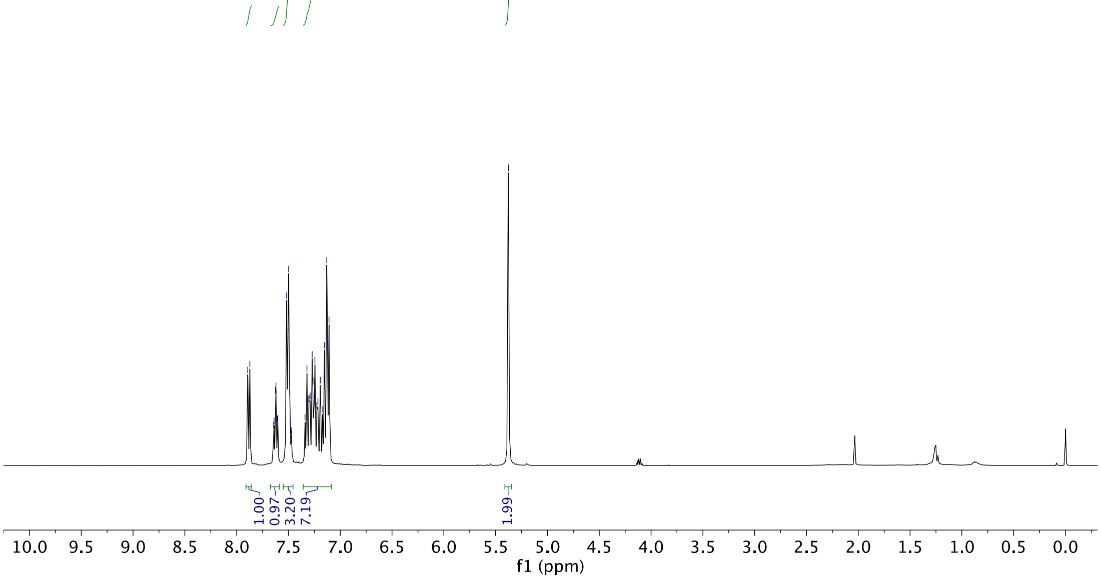

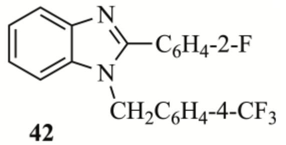

42

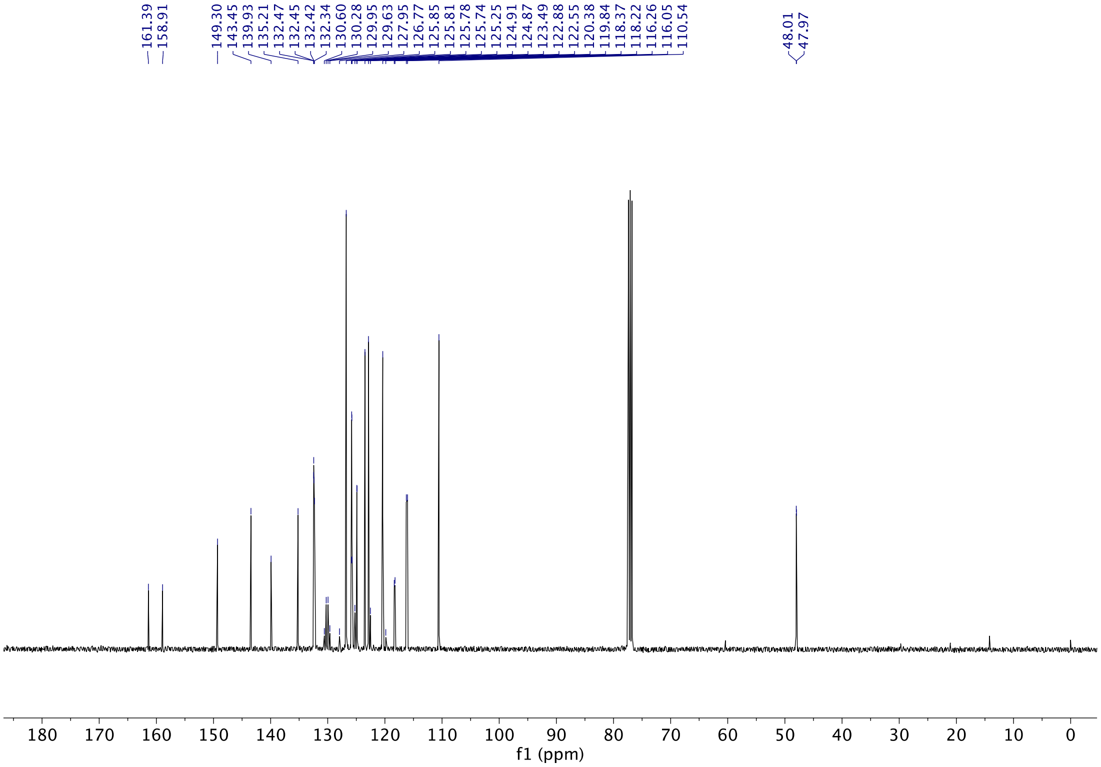

^{19}F spectrum for 2-(2-Fluorophenyl)-1-(4-(trifluoromethyl)benzyl)-1*H*-benzo[*d*]imidazole (**42**)

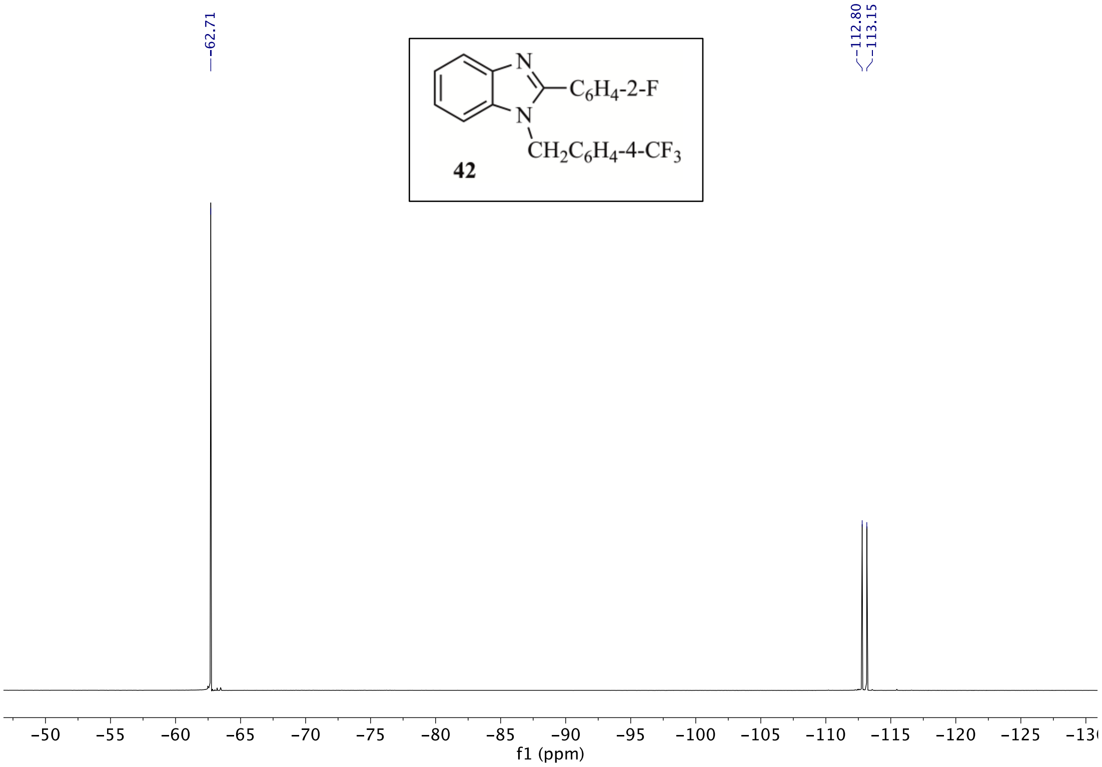

^{1}H and ^{13}C spectra for 2-Nitro-*N*-phenylaniline (**7i**)

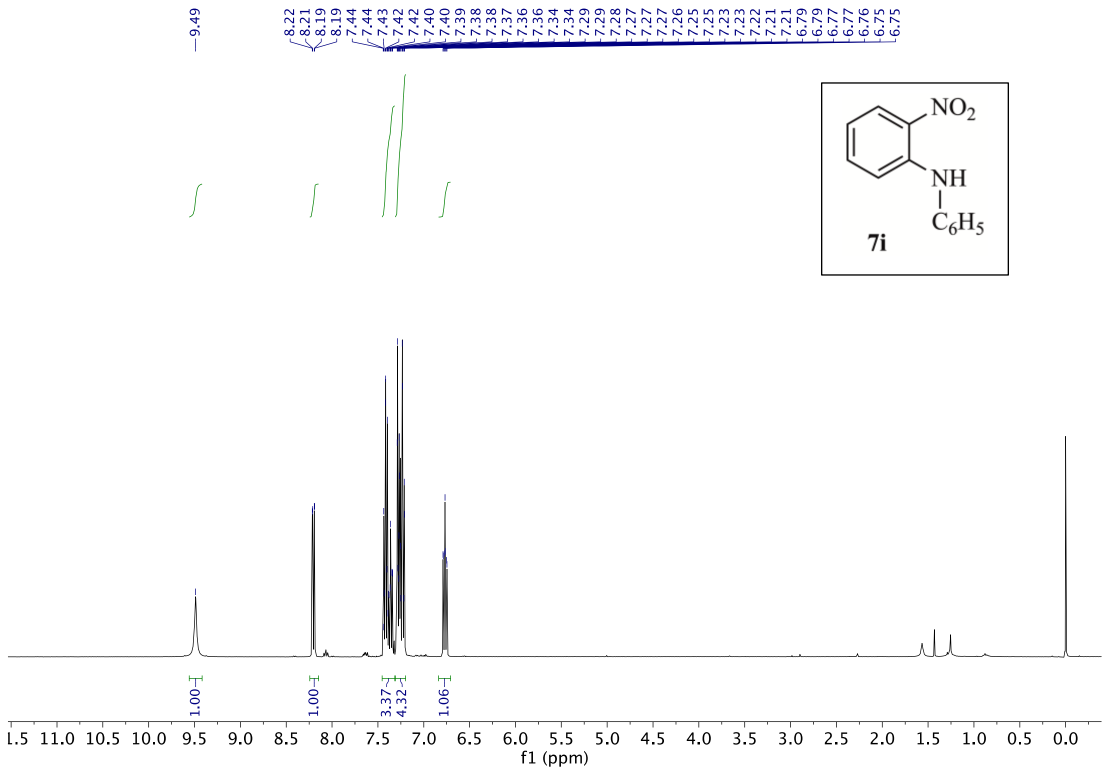

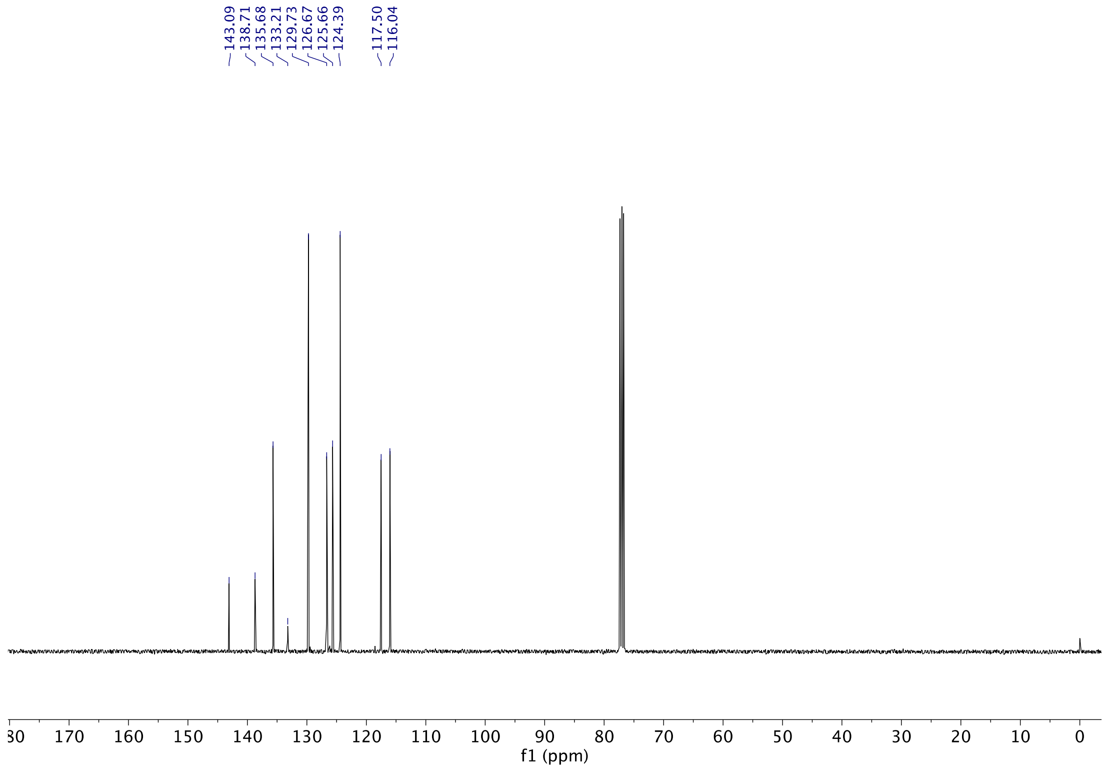

^{1}H and ^{13}C spectra for 2-Methyl-1-phenyl-1*H*-benzo[*d*]imidazole (**43**)

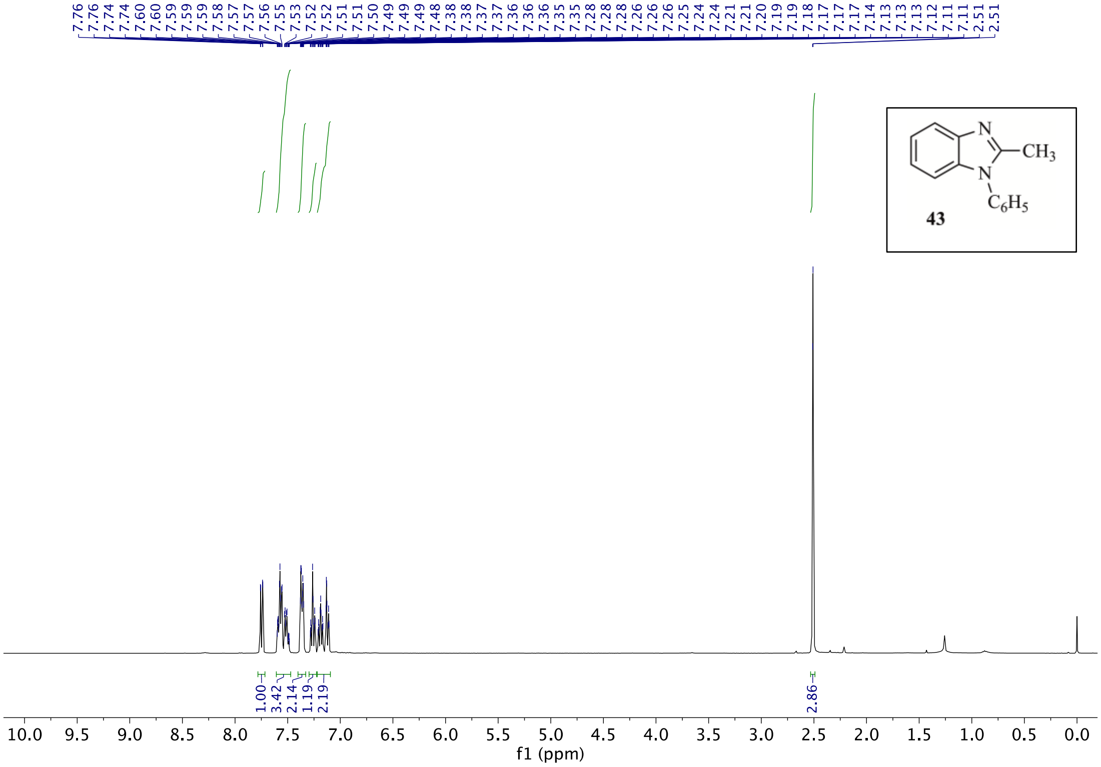

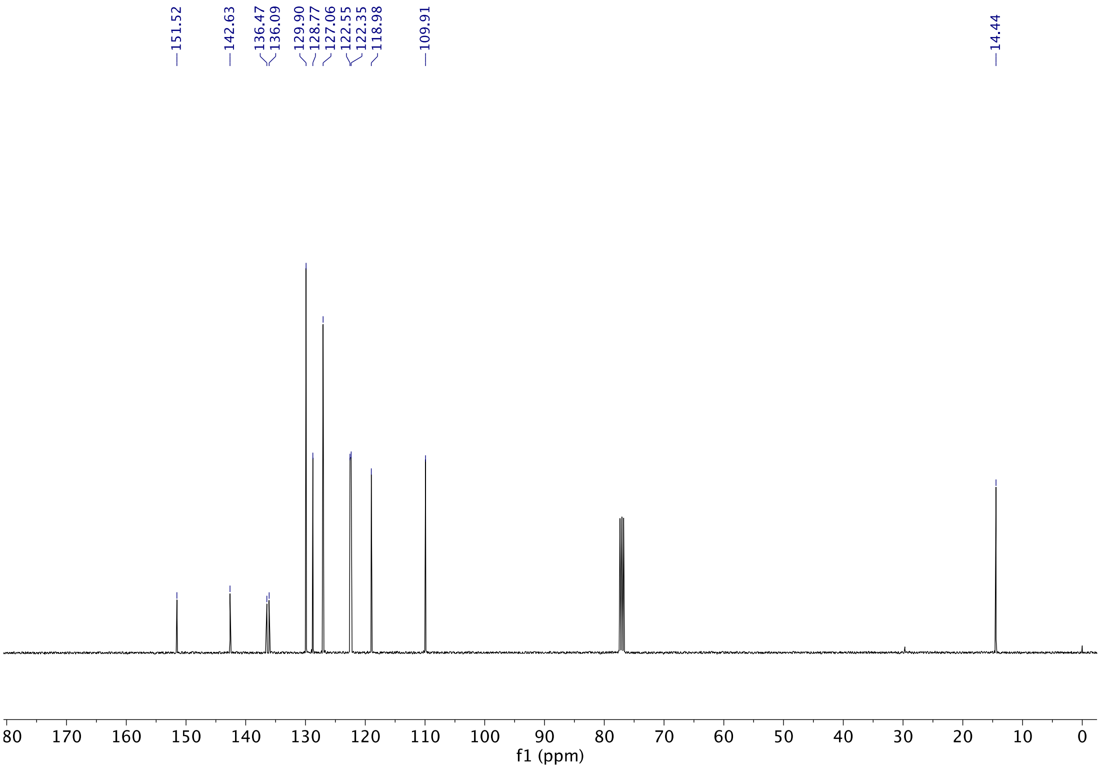

^{1}H and ^{13}C spectra for 1-Phenyl-2-(4-(trifluoromethyl)phenyl)-1*H*-benzo[*d*]imidazole (**44**)

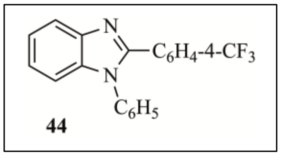

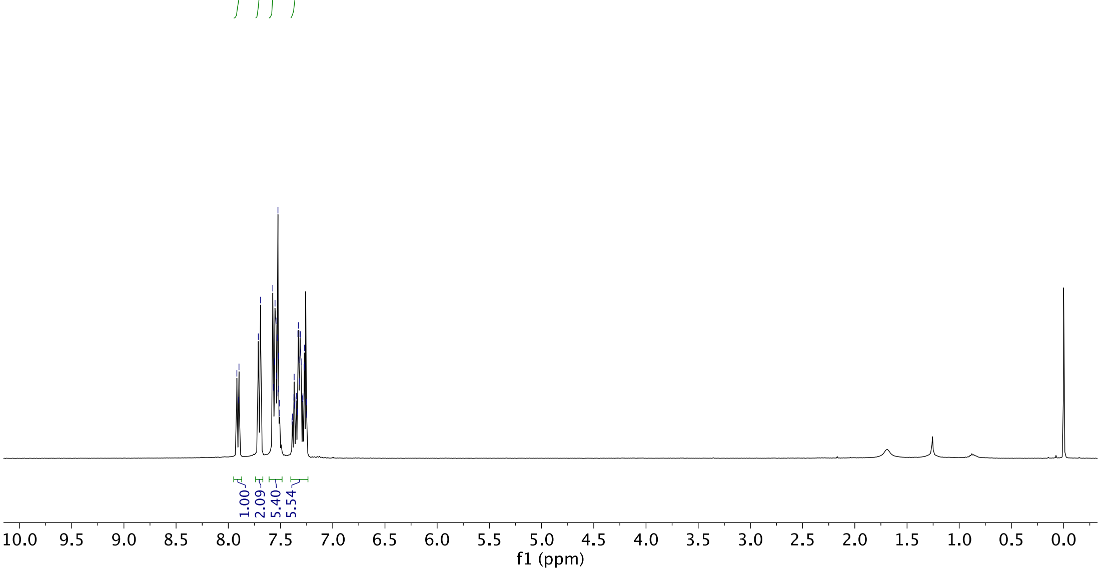

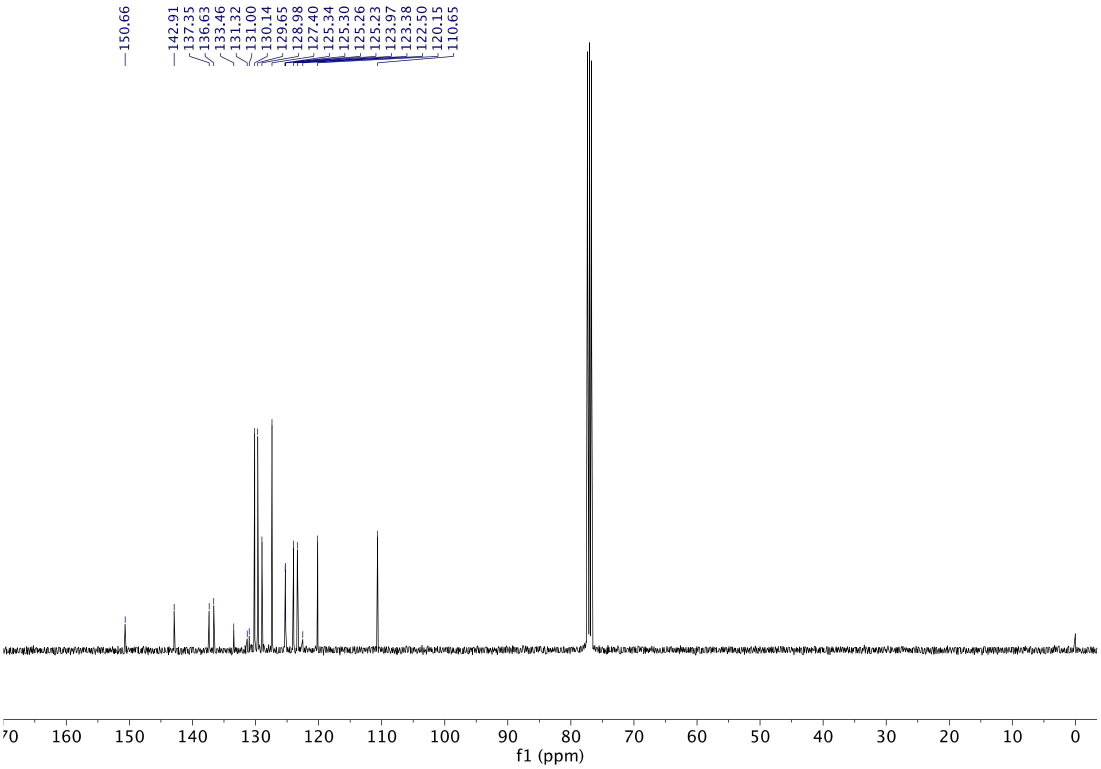

^{19}F spectrum for 1-Phenyl-2-(4-(trifluoromethyl)phenyl)-1*H*-benzo[*d*]imidazole (**44**)

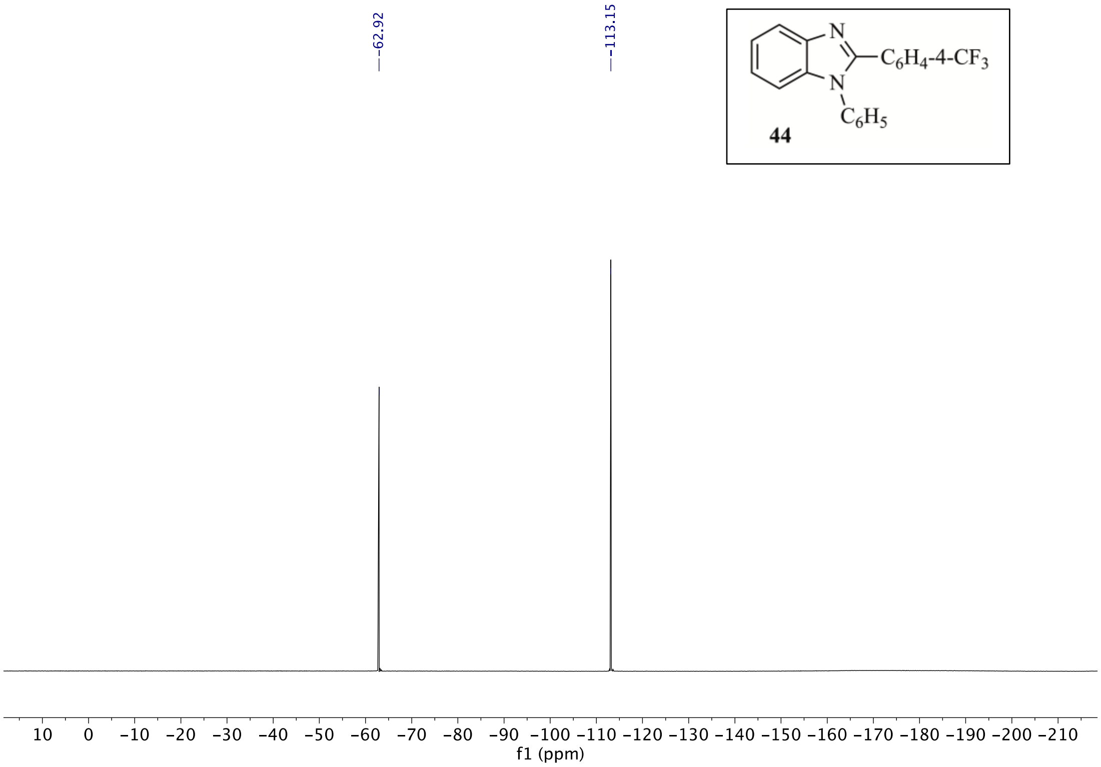

^{1}H and ^{13}C spectra for *N*-(4-Methoxyphenyl)-2-nitroaniline (**7j**)

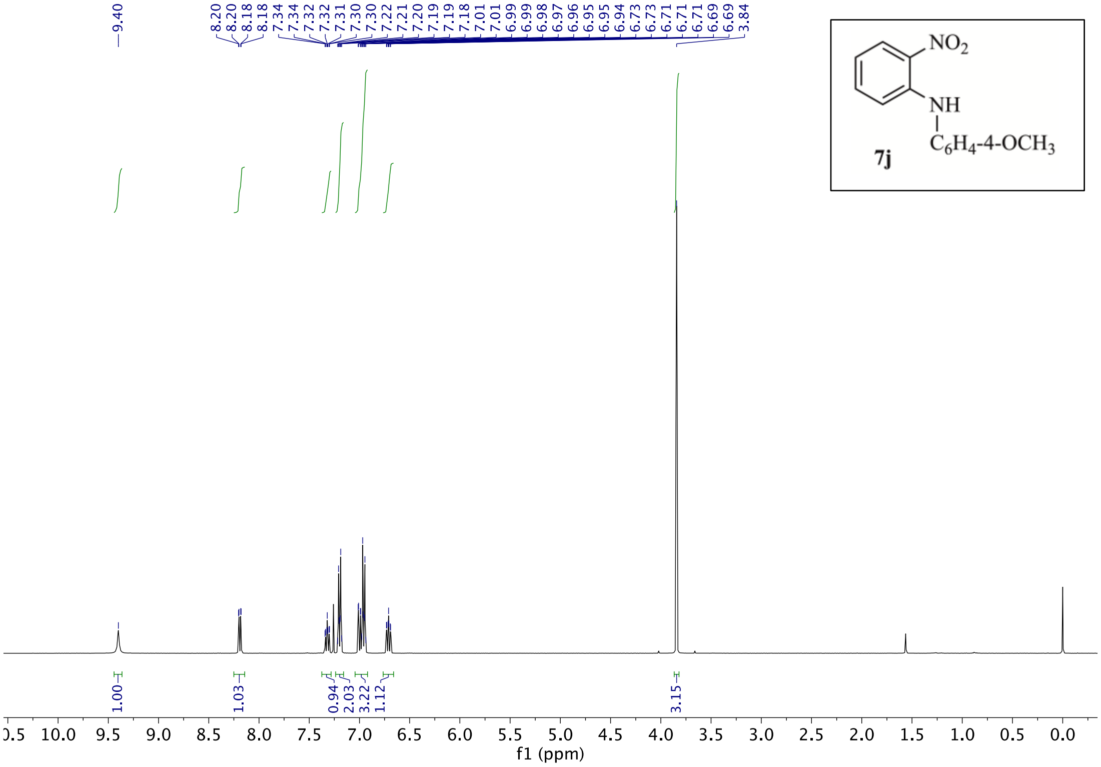

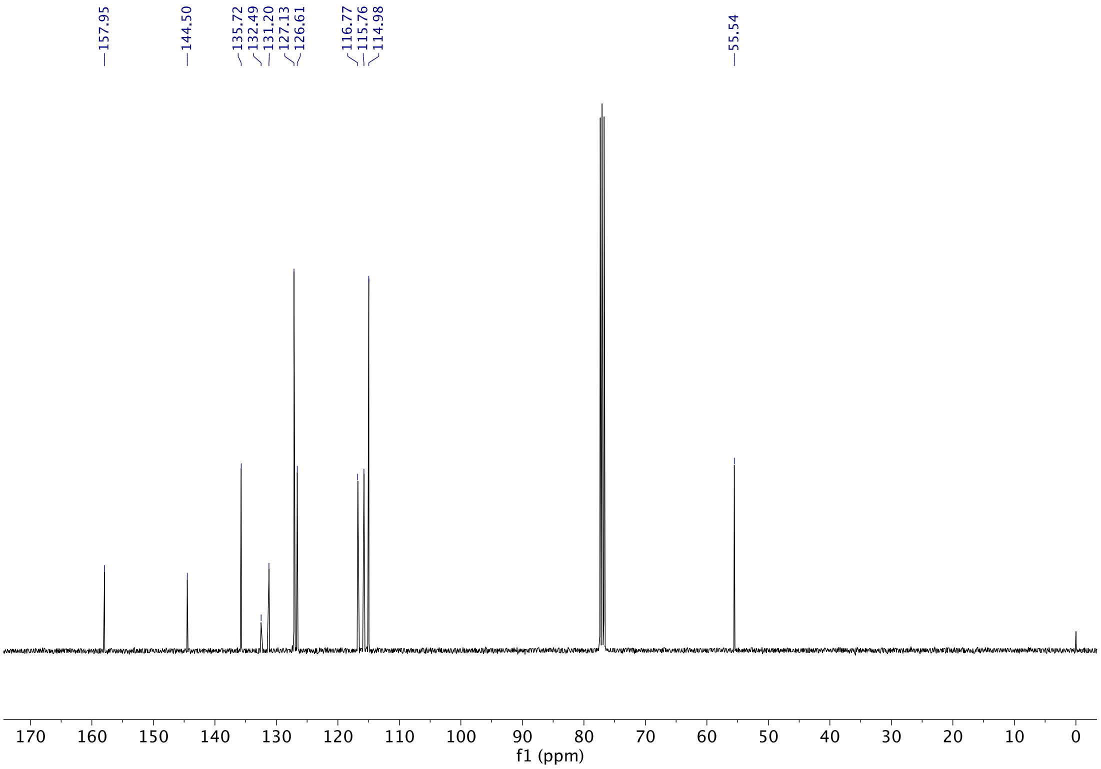

^{1}H and ^{13}C spectra for 1-(4-Methoxyphenyl)-2-methyl-1*H*-benzo[*d*]imidazole (**45**)

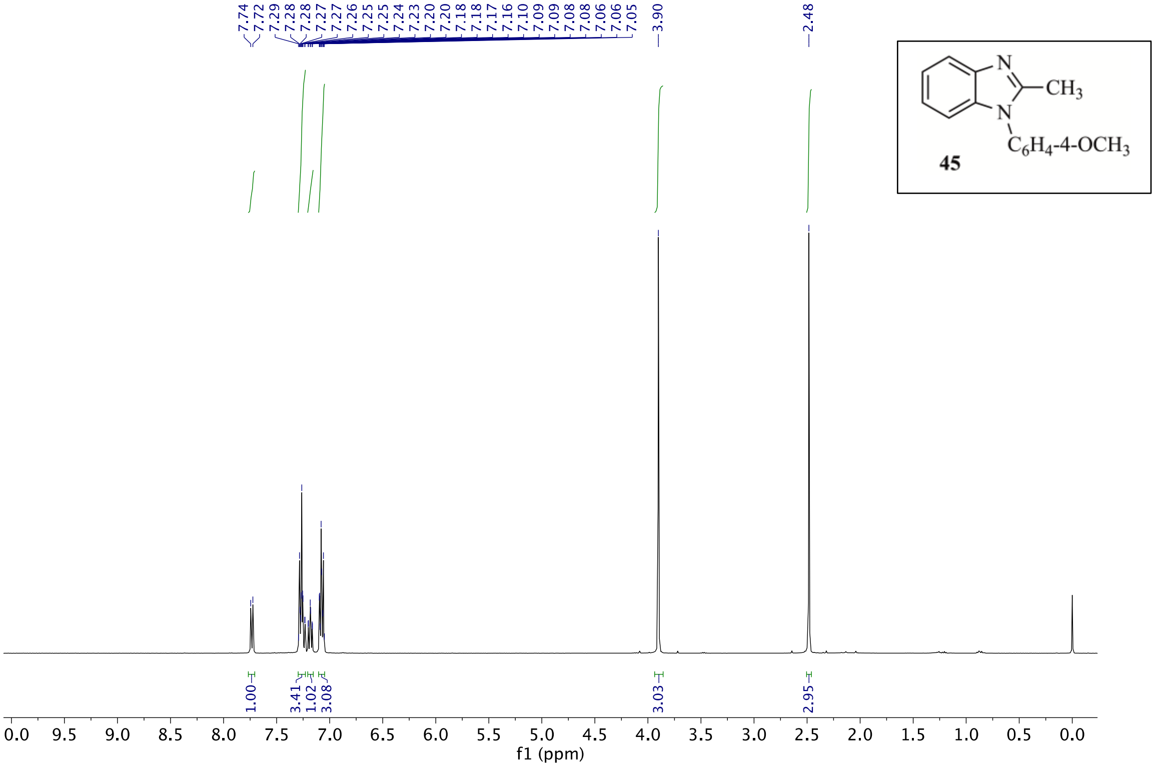

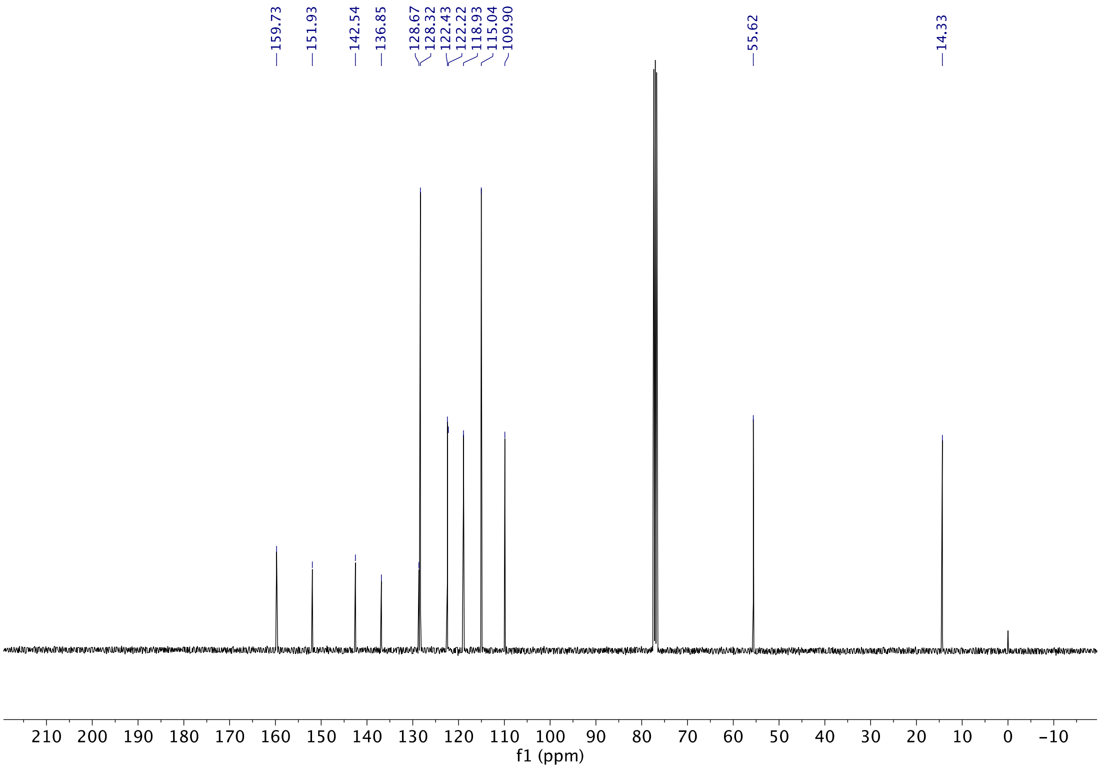

^{1}H and ^{13}C spectra for 1-(4-Methoxyphenyl)-2-phenyl-1*H*-benzo[*d*]imidazole (**46**)

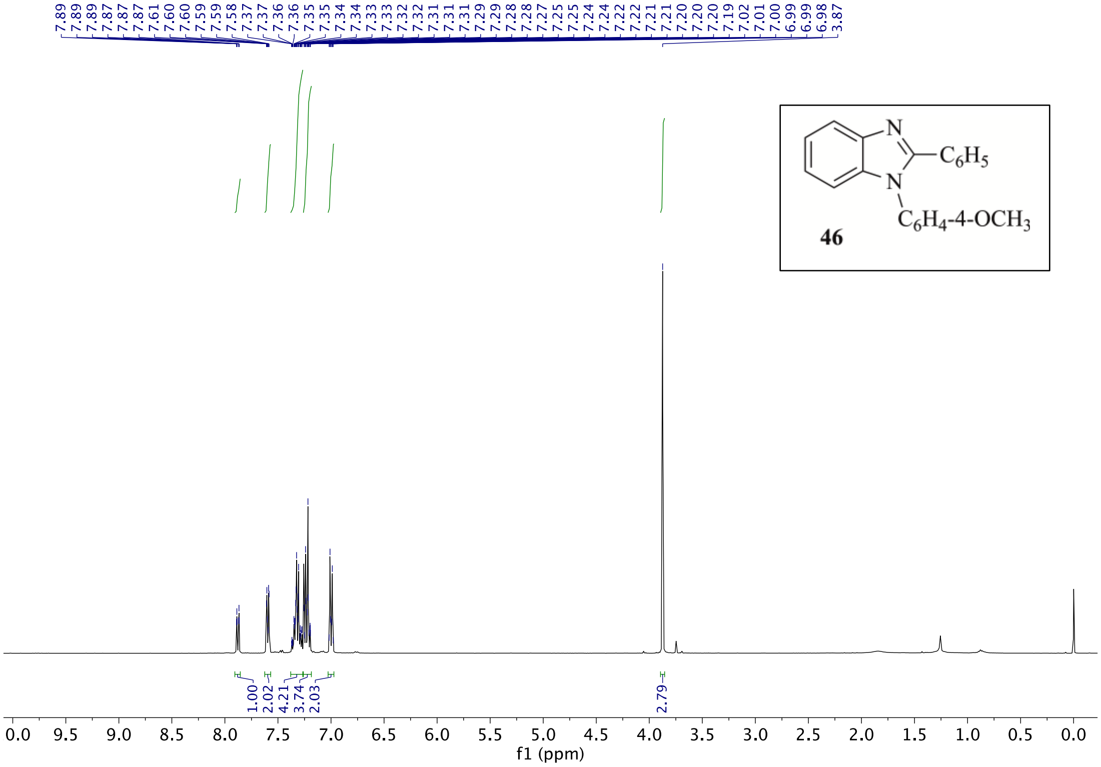

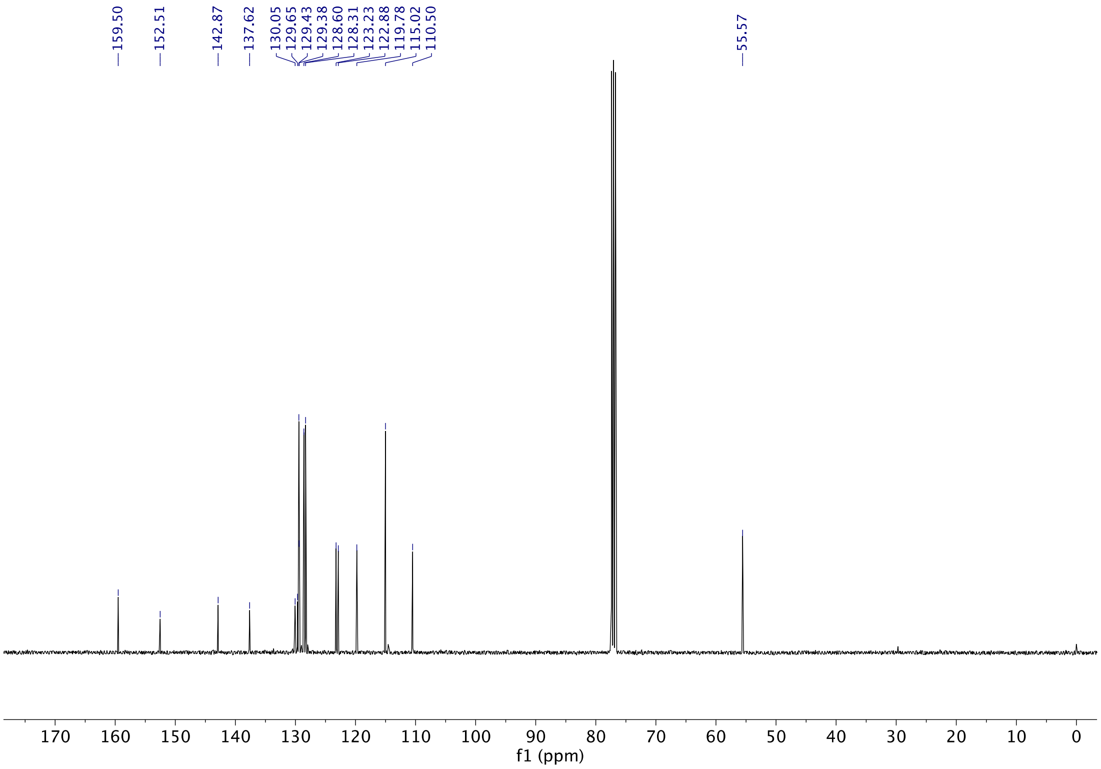

Supplement: Supplementary file 1 [file molecules-31-02150-s001.zip › molecules-4366729-supplementary.pdf]
